# Supplementary material for: Sequential bilateral accelerated theta burst stimulation in adolescents with suicidal ideation associated with major depressive disorder: Protocol for a randomized controlled trial
Source: PLoS One. 2023 Apr 13;18(4):e0280010. doi: 10.1371/journal.pone.0280010 (PMC10101506; doi:10.1371/journal.pone.0280010)
Supplement: S1 Checklist — (PDF) [file pone.0280010.s001.pdf]

SPIRIT
STANDARD PROTOCOL ITEMS: RECOMMENDATIONS FOR INTERVENTIONAL TRIALS

Appendix 1. SPIRIT 2013 Checklist: Recommended items to address in a clinical trial protocol and related documents*

Section/item	Item No	Description	Addressed on page number
**Administrative information**			
Title	1	Descriptive title identifying the study design, population, interventions, and, if applicable, trial acronym	__1___________
Trial registration	2a	Trial identifier and registry name. If not yet registered, name of intended registry	__4___________
	2b	All items from the World Health Organization Trial Registration Data Set	__Admin information below__________
Protocol version	3	Date and version identifier	__Admin info____
Funding	4	Sources and types of financial, material, and other support	__35, Admin info____
Roles and responsibilities	5a	Names, affiliations, and roles of protocol contributors	_____1-2, 36____
	5b	Name and contact information for the trial sponsor	_Admin info____
	5c	Role of study sponsor and funders, if any, in study design; collection, management, analysis, and interpretation of data; writing of the report; and the decision to submit the report for publication, including whether they will have ultimate authority over any of these activities	_Admin info__

	5d	Composition, roles, and responsibilities of the coordinating centre, steering committee, endpoint adjudication committee, data management team, and other individuals or groups overseeing the trial, if applicable (see Item 21a for data monitoring committee)	__Admin info___
**Introduction**			
Background and rationale	6a	Description of research question and justification for undertaking the trial, including summary of relevant studies (published and unpublished) examining benefits and harms for each intervention	____7_________
	6b	Explanation for choice of comparators	____10_________
Objectives	7	Specific objectives or hypotheses	____10-11_____
Trial design	8	Description of trial design including type of trial (eg, parallel group, crossover, factorial, single group), allocation ratio, and framework (eg, superiority, equivalence, noninferiority, exploratory)	____11_____
**Methods: Participants, interventions, and outcomes**			
Study setting	9	Description of study settings (eg, community clinic, academic hospital) and list of countries where data will be collected. Reference to where list of study sites can be obtained	____11_________
Eligibility criteria	10	Inclusion and exclusion criteria for participants. If applicable, eligibility criteria for study centres and individuals who will perform the interventions (eg, surgeons, psychotherapists)	_____11-12_____
Interventions	11a	Interventions for each group with sufficient detail to allow replication, including how and when they will be administered	_____12-13____
	11b	Criteria for discontinuing or modifying allocated interventions for a given trial participant (eg, drug dose change in response to harms, participant request, or improving/worsening disease)	____13_________
	11c	Strategies to improve adherence to intervention protocols, and any procedures for monitoring adherence (eg, drug tablet return, laboratory tests)	_____13-14_____
	11d	Relevant concomitant care and interventions that are permitted or prohibited during the trial	____14_________

Outcomes	12	Primary, secondary, and other outcomes, including the specific measurement variable (eg, systolic blood pressure), analysis metric (eg, change from baseline, final value, time to event), method of aggregation (eg, median, proportion), and time point for each outcome. Explanation of the clinical relevance of chosen efficacy and harm outcomes is strongly recommended	____14-15______
Participant timeline	13	Time schedule of enrolment, interventions (including any run-ins and washouts), assessments, and visits for participants. A schematic diagram is highly recommended (see Figure)	___15, T1_______
Sample size	14	Estimated number of participants needed to achieve study objectives and how it was determined, including clinical and statistical assumptions supporting any sample size calculations	____17-18_____
Recruitment	15	Strategies for achieving adequate participant enrolment to reach target sample size	____19-20_____

Methods: Assignment of interventions (for controlled trials)

Allocation:

Sequence generation	16a	Method of generating the allocation sequence (eg, computer-generated random numbers), and list of any factors for stratification. To reduce predictability of a random sequence, details of any planned restriction (eg, blocking) should be provided in a separate document that is unavailable to those who enrol participants or assign interventions	__20___________
Allocation concealment mechanism	16b	Mechanism of implementing the allocation sequence (eg, central telephone; sequentially numbered, opaque, sealed envelopes), describing any steps to conceal the sequence until interventions are assigned	___20__________
Implementation	16c	Who will generate the allocation sequence, who will enrol participants, and who will assign participants to interventions	__20___________
Blinding (masking)	17a	Who will be blinded after assignment to interventions (eg, trial participants, care providers, outcome assessors, data analysts), and how	___20-21_______
	17b	If blinded, circumstances under which unblinding is permissible, and procedure for revealing a participant's allocated intervention during the trial	___21__________

Methods: Data collection, management, and analysis

Data collection methods	18a	Plans for assessment and collection of outcome, baseline, and other trial data, including any related processes to promote data quality (eg, duplicate measurements, training of assessors) and a description of study instruments (eg, questionnaires, laboratory tests) along with their reliability and validity, if known. Reference to where data collection forms can be found, if not in the protocol	___21, T2______
	18b	Plans to promote participant retention and complete follow-up, including list of any outcome data to be collected for participants who discontinue or deviate from intervention protocols	___21-22_______
Data management	19	Plans for data entry, coding, security, and storage, including any related processes to promote data quality (eg, double data entry; range checks for data values). Reference to where details of data management procedures can be found, if not in the protocol	____22-23_____
Statistical methods	20a	Statistical methods for analysing primary and secondary outcomes. Reference to where other details of the statistical analysis plan can be found, if not in the protocol	_23-27_________
	20b	Methods for any additional analyses (eg, subgroup and adjusted analyses)	__25_______
	20c	Definition of analysis population relating to protocol non-adherence (eg, as randomised analysis), and any statistical methods to handle missing data (eg, multiple imputation)	__26-27________

Methods: Monitoring

Data monitoring	21a	Composition of data monitoring committee (DMC); summary of its role and reporting structure; statement of whether it is independent from the sponsor and competing interests; and reference to where further details about its charter can be found, if not in the protocol. Alternatively, an explanation of why a DMC is not needed	_____27-28_____
	21b	Description of any interim analyses and stopping guidelines, including who will have access to these interim results and make the final decision to terminate the trial	______28-29__
Harms	22	Plans for collecting, assessing, reporting, and managing solicited and spontaneously reported adverse events and other unintended effects of trial interventions or trial conduct	___29-31_______
Auditing	23	Frequency and procedures for auditing trial conduct, if any, and whether the process will be independent from investigators and the sponsor	____31_________

Ethics and dissemination

Research ethics approval	24	Plans for seeking research ethics committee/institutional review board (REC/IRB) approval	_34 (Approved)__
Protocol amendments	25	Plans for communicating important protocol modifications (eg, changes to eligibility criteria, outcomes, analyses) to relevant parties (eg, investigators, REC/IRBs, trial participants, trial registries, journals, regulators)	____33_________
Consent or assent	26a	Who will obtain informed consent or assent from potential trial participants or authorised surrogates, and how (see Item 32)	______31_______
	26b	Additional consent provisions for collection and use of participant data and biological specimens in ancillary studies, if applicable	_____N/A________
Confidentiality	27	How personal information about potential and enrolled participants will be collected, shared, and maintained in order to protect confidentiality before, during, and after the trial	_____31-33____
Declaration of interests	28	Financial and other competing interests for principal investigators for the overall trial and each study site	_____35________
Access to data	29	Statement of who will have access to the final trial dataset, and disclosure of contractual agreements that limit such access for investigators	_____35________
Ancillary and post-trial care	30	Provisions, if any, for ancillary and post-trial care, and for compensation to those who suffer harm from trial participation	____N/A________
Dissemination policy	31a	Plans for investigators and sponsor to communicate trial results to participants, healthcare professionals, the public, and other relevant groups (eg, via publication, reporting in results databases, or other data sharing arrangements), including any publication restrictions	_____34________
	31b	Authorship eligibility guidelines and any intended use of professional writers	_____36________
	31c	Plans, if any, for granting public access to the full protocol, participant-level dataset, and statistical code	_____33________
**Appendices**			
Informed consent materials	32	Model consent form and other related documentation given to participants and authorised surrogates	__Appendix 1____

Biological specimens	33	Plans for collection, laboratory evaluation, and storage of biological specimens for genetic or molecular analysis in the current trial and for future use in ancillary studies, if applicable	__N/A_______

*It is strongly recommended that this checklist be read in conjunction with the SPIRIT 2013 Explanation & Elaboration for important clarification on the items. Amendments to the protocol should be tracked and dated. The SPIRIT checklist is copyrighted by the SPIRIT Group under the Creative Commons "Attribution-NonCommercial-NoDerivs 3.0 Unported" license.

Administrative Information

Note: the numbers in braces in this protocol refer to the SPIRIT checklist items. The order of the items has been modified to group similar items. (Chan AW, Tetzlaff JM, Altman DG, et al. SPIRIT 2013 statement: defining standard protocol items for clinical trials. *Ann Intern Med.* 2013;158:200-207. doi:10.7326/0003-4819-158-3-201302050-00583)

Title	A Randomized Controlled Trial of Sequential Bilateral Accelerated Theta Burst Stimulation in Adolescents With Suicidal Ideation Associated With Major Depressive Disorder
Trial registration	Investigational Device Exemption (IDE) Number: G200220<br>ClinicalTrials.gov Identifier: NCT04701840
Protocol version	June 1, 2021, Data and Safety Monitoring Board DSMB (Approved Version)
Funding	US National Institute of Mental Health (NIMH) Grant 1 R01MH124655-01<br>The content of this protocol and publication is solely the responsibility of the authors and does not necessarily represent the official views of the National Institutes of Health.

Author details	Principal Investigator (PI): Paul E. Croarkin, DO, MS. Co-Investigators: Deniz Yuruk, MD; Can Ozger, BS; Juan F. Garzon, MD; Jarrod M. Leffler, PhD; Julia Shekunov, MD; Jennifer L. Vande Voort, MD; Michael J. Zaccariello, PhD, LP; Paul A. Nakonezny, PhD
Name and contact information for the trial sponsor	National Institute of Mental Health<br>Office of Science Policy, Planning, and Communications<br>6001 Executive Boulevard<br>Room 6200, MSC 9663<br>Bethesda, MD 20892-9663
Role of sponsor	The sponsor-investigator will promptly review documented Unanticipated Adverse Device Effects and as necessary shall report the results from such evaluation to the US Food and Drug Administration (FDA) and DSMB within 10 workdays and to the Mayo Clinic IRB within 5 workdays of initial notice of the effect. Thereafter the sponsor-investigator will submit such additional reports concerning the effect as

requested.

It is the responsibility of the sponsor-investigator to oversee the safety of the study. This safety monitoring will include careful assessment and appropriate reporting of adverse events as noted earlier, as well as the construction and implementation of a site data and safety monitoring plan.

Clinical monitoring will be conducted by the NIMH Clinical Research Education, Support, and Training (CREST) program. This monitoring includes document review, database review, and review of regulatory documents.

An NIMH-constituted Data and Safety Monitoring Board (DSMB) will oversee the study in accordance with the NIMH DSMB charter guidelines.
