# Supplementary material for: Sequential bilateral accelerated theta burst stimulation in adolescents with suicidal ideation associated with major depressive disorder: Protocol for a randomized controlled trial
Source: PLoS One. 2023 Apr 13;18(4):e0280010. doi: 10.1371/journal.pone.0280010 (PMC10101506; doi:10.1371/journal.pone.0280010)
Supplement: S1 File — (PDF) [file pone.0280010.s002.pdf]

S 2. Study Administrative Information

Note: the numbers in braces in this protocol refer to the SPIRIT checklist items. The order of the items has been modified to group similar items. (Chan AW, Tetzlaff JM, Altman DG, et al. SPIRIT 2013 statement: defining standard protocol items for clinical trials. *Ann Intern Med.* 2013;158:200-207. doi:10.7326/0003-4819-158-3-201302050-00583)

Title{1}	A Randomized Controlled Trial of Sequential Bilateral Accelerated Theta Burst Stimulation in Adolescents With Suicidal Ideation Associated With Major Depressive Disorder
Trial registration{2a and 2b}	Investigational Device Exemption (IDE) Number: G200220<br>ClinicalTrials.gov Identifier: NCT04701840
Protocol version{3}	June 1, 2021, Data and Safety Monitoring Board DSMB (Approved Version)
Funding{4}	US National Institute of Mental Health (NIMH) Grant 1 R01MH124655-01<br>The content of this protocol and publication is solely the responsibility of the authors and does not necessarily represent the official views of the National Institutes of Health.
Author details{5a}	Principal Investigator (PI): Paul E. Croarkin, DO, MS. Co-Investigators: Deniz Yuruk, MD; Can Ozger, BS; Juan F. Garzon, MD; Jarrod M. Leffler, PhD; Julia Shekunov, MD; Jennifer L. Vande Voort, MD; Michael J. Zaccariello, PhD, LP; Paul A. Nakonezny, PhD
Name and contact information for the trial sponsor{5b}	Paul E. Croarkin, DO, MS<br>Address: Mayo Clinic Depression Center<br>Department of Psychiatry and Psychology

	200 First Street SW<br>Rochester, Minnesota 55905<br>Telephone Number:<br>Fax Number:<br>E-mail Address: croarkin.paul@mayo.edu
Role of sponsor{5c}	The sponsor-investigator will promptly review documented Unanticipated Adverse Device Effects and as necessary shall report the results from such evaluation to the US Food and Drug Administration (FDA) and DSMB within 10 workdays and to the Mayo Clinic IRB within 5 workdays of initial notice of the effect. Thereafter the sponsor-investigator will submit such additional reports concerning the effect as requested.<br>It is the responsibility of the sponsor-investigator to oversee the safety of the study. This safety monitoring will include careful assessment and appropriate reporting of adverse events as noted earlier, as well as the construction and implementation of a site data and safety monitoring plan.<br>Clinical monitoring will be conducted by the NIMH Clinical Research Education, Support, and Training (CREST) program. This monitoring includes document review, database review, and review of regulatory documents.<br>An NIMH-constituted Data and Safety Monitoring Board (DSMB) will oversee the study in accordance with the NIMH DSMB charter guidelines.
