# Supplementary material for: Sequential bilateral accelerated theta burst stimulation in adolescents with suicidal ideation associated with major depressive disorder: Protocol for a randomized controlled trial
Source: PLoS One. 2023 Apr 13;18(4):e0280010. doi: 10.1371/journal.pone.0280010 (PMC10101506; doi:10.1371/journal.pone.0280010)
Supplement: S2 File — (PDF) [file pone.0280010.s003.pdf]

MAYO CLINIC

Name and Clinic Number

Approval Date: **November 10, 2022**
Not to be used after: **November 9, 2023**

FOR INFORMATIONAL PURPOSES ONLY

RESEARCH PARTICIPANT CONSENT AND PRIVACY AUTHORIZATION FORM

Study Title: A Randomized Controlled Trial of Sequential Bilateral Accelerated Theta Burst Stimulation in Adolescents with Suicidal Ideation Associated with Major Depressive Disorder (Ages 12-17)

IRB#: 20-009630

Principal Investigator: Dr. Paul Croarkin and Colleagues

Key Study Information

This section provides a brief summary of the study. It is important for you to understand why the research is being done and what it will involve before you decide. **Please take the time to read the entire consent form carefully and talk to a member of the research team before making your decision.** You should not sign this form if you have any questions that have not been answered. **<u>You will sign this form to provide informed consent and your child will sign the form to provide assent.</u>**	
**It's Your Choice**	This is a research study. Being in this research study is your choice; you do not have to participate. If you decide to join, you can still stop at any time. You should only participate if you want to do so. You will not lose any services, benefits or rights you would normally have if you choose not to take part.
**Research Purpose**	The purpose of this research is to conduct the first study of an investigational treatment; this means it is a pilot study using an investigational device. The investigational treatment is called sequential bilateral accelerated theta burst stimulation. Sequential bilateral accelerated theta burst stimulation is a new type of repetitive transcranial magnetic stimulation (TMS) that is delivered to both sides of the brain three times a day for 10 days. Throughout this consent and assent form the abbreviation "TMS" will be used to describe research treatment in the study (again the technical name is sequential bilateral accelerated theta burst stimulation). Sham stimulation looks like TMS but has less than 5% of the magnetic field of TMS and is not thought to have an active treatment effect.

	You have been asked to take part in this research because you are an adolescent between the ages of 12 and 18 with suicidal ideation and major depressive disorder. The plan is to have about 80 people with similar symptoms take part in the study at Mayo Clinic. The plan is to have about 80 people with similar symptoms take part in the study at Mayo Clinic.
**What's Involved**	During the treatment part of the study, you will have a screening visit, baseline visit, and 10 days of treatment. Each of these visit days will take up to 5 hours, so you should plan for this (e.g., this means that you may miss some school, work, or extracurricular activities). Study participation involves completing baseline assessment forms, brain measures before and after treatment, and 10 days of treatment with TMS or a sham stimulation. During the 10 days of treatment all patients will participate in a talk therapy program focused on coping skills. After treatment, patients will have monthly follow up visits for one year for assessments. The talk therapy will be provided as part of the study so any other talk therapy with non-study providers must be discontinued during the 10 days of treatment with TMS. This means that you will need to discontinue contact with any other outpatient therapist for 2 weeks.
**Key Information**	Patients in this study will receive a new treatment form of TMS or a sham comparison while participating in talk therapy. Sometimes patients find this new treatment uncomfortable and in rare cases it gives patients a brief seizure. The alternative to participating in the study is to have standard visits with a psychiatrist or therapist.
**Learn More**	If you are interested in learning more about this study, read the rest of this form carefully. The information in this form will help you decide if you want to participate in this research or not. A member of our research team will talk with you about taking part in this study before you sign this form. If you have questions at any time, please ask us.

Making Your Decision

Taking part in research is your decision. Take your time to decide. Feel free to discuss the study with your family, friends, and healthcare provider before you make your decision. Taking part in this study is completely voluntary and you do not have to participate.

If you decide to take part in this research study, you will sign this consent form and your child will sign the form to provide assent to show that you want to take part. We will give you either a printed or electronic copy of this form to keep. A copy of this form will be put in your medical record.

If you are signing this consent form for someone else, “you” in the consent form refers to the participant. The child participant will also sign the form to provide assent.

For purposes of this form, Mayo Clinic refers to Mayo Clinic in Arizona, Florida and Rochester, Minnesota; Mayo Clinic Health System; and all owned and affiliated clinics, hospitals, and entities.

MAYO CLINIC

Approval Date: **November 10, 2022**
Not to be used after: **November 9, 2023**

Contact Information

If you have questions about …	You can contact …
▪ Study tests and procedures<br>▪ Materials you receive<br>▪ Research-related appointments<br>▪ Research-related concern or complaint<br>▪ Research-related injuries or emergencies<br>▪ Withdrawing from the research study	**Principal Investigator:** Paul Croarkin, DO, MS<br>**Phone:** (507) 293-2557<br><br>**Study Team Contact:** Jeremy Weiss<br>**Phone:** (507) 293-5659<br><br>**Institution Name and Address:**<br>Mayo Clinic<br>Department of Psychiatry and Psychology<br>200 First Street SW<br>Rochester, MN 55905
▪ Rights of a research participant	**Mayo Clinic Institutional Review Board (IRB)**<br>**Phone:** (507) 266-4000<br>**Toll-Free:** (866) 273-4681
▪ Rights of a research participant<br>▪ Any research-related concern or complaint<br>▪ Use of your Protected Health Information<br>▪ Stopping your authorization to use your Protected Health Information<br>▪ Withdrawing from the research study	**Research Participant Advocate (RPA)**<br>**(The RPA is independent of the Study Team)**<br>**Phone:** (507) 266-9372<br>**Toll-Free:** (866) 273-4681<br><br>**E-mail:** researchparticipantadvocate@mayo.edu
▪ Billing or insurance related to this research study	**Patient Account Services**<br>**Toll-Free:** (844) 217-9591

Other Information:

A description of this clinical trial will be available on http://www.ClinicalTrials.gov, as required by U.S. Law. This Web site will not include information that can identify you. At most, the Web site will include a summary of the results. You can search this Web site at any time.

A description of this clinical trial will be available on the mayoclinic.org research study web site.

Why are you being asked to take part in this research study?

You are being asked to take part in this research study because you are an adolescent between the ages of 12 and 18 with suicidal ideation and Major Depressive Disorder. The plan is to have about 80 people with similar symptoms take part in this study at Mayo Clinic.

Why is this research study being done?

The purpose of this pilot study is to gather information regarding the feasibility, safety, and benefits of a new form of TMS treatment for suicidal ideation in adolescents with Major Depressive Disorder. The investigators hope to learn if this TMS treatment improves suicidal ideation over 10 days and clinical outcomes over 1 year of follow-up. The investigators will also collect measures of brain function before and after TMS treatment.

Therapy with TMS has U.S. Food and Drug Administration (FDA) clearance for the treatment of adults with Major Depressive Disorder who have failed prior antidepressant medication therapy. Therapy with TMS for children and adolescents with Major Depressive Disorder has been studied previously but is considered investigational in this study. The FDA has granted permission to use the magnetic stimulator in this study (called MagVenture) under an Investigational Device Exemption (IDE).

Information you should know

Who is Funding the Study?

This study is funded by the National Institute of Mental Health (NIMH). The stimulators and supplies needed for the study treatments will be provided by MagVenture.

Information Regarding Conflict of Interest:

Your healthcare provider may be referring you to this research study. If your healthcare provider is also an investigator on this study, there is the chance that his or her responsibilities for the study could influence his or her recommendation for your participation.

If you prefer, your healthcare provider will be happy to refer you to another investigator on the research study team for you to decide if you want to participate in the study and to see you for the research study activities while you are in the study.

How long will you be in this research study?

There is a treatment part of the study that lasts 2 weeks. You then will be asked to follow-up with the study team once a month for 1 year. So if you are willing you will be in the research study for approximately 13 months.

What will happen to you while you are in this research study?

If you agree to be in the study, you will be asked to participate in the following:

<u>Screening Visit</u>
The Screening Visit will help us determine if you are eligible for the study and will take up to 5 hours. The Principal Investigator will review the results of these tests and procedures. If you are not eligible, the Principal Investigator will tell you why. At this visit, we will:

1. Review the informed consent form and answer any questions you have. You must sign the informed consent from prior to completing any study procedures. Your child must also sign this form to provide assent. A signed copy will be provided to you for your records.
2. Ask about your psychiatric and medical history.
3. Review information about medications you have taken and/or are currently taking.
4. Discontinue any medications or treatments that are not allowed during the study.
5. Have an interview with a doctor and/or other study staff member to determine your diagnoses and level of depression. These questions help us make sure that the study is right for you.

   These will include the following forms:
   - M.I.N.I./M.I.N.I.KID (Min International Neuropsychiatric Interview for Children and Adolescents) is a short structured diagnostic interview for psychiatric disorders in children and adolescents.
   - BANPS (Brief Affective Neuroscience Personality Scale) is a 33-question assessment about your personality.
   - CDRS-R (Children’s Depression Rating Scale, Revised) is a 17-question form that helps describe your level of depression. Parents help answer 14 of the 17 questions.
   - CTQ (Childhood Trauma Questionnaire) is a 28-question form that helps describe adversities you faced.

- BDI-II (Beck Depression Inventory II) is a 21-question form that explains your symptoms of depression.
- CGI (Clinical Global Impression) is a form the study team uses to rate your mood
- Columbia Suicide Severity Rating Scale (C-SSRS) is a question that assesses your suicidal ideation.
- YMRS (Young Mania Rating Scale) is a form that rates any symptoms of bipolar disorder you might have.
- You will complete a PDS (Pubertal Development Scale and Tanner Staging) form that (with line drawings and questions) determines how far along you are in puberty.
- A Demographics form that asks about your racial and gender identity, and education of your parents.

6. Have a medical history collected, physical examination, collect vital signs (height, weight, blood pressure, and heart rate), and urine drug screen.
    a. To participate in the study you must let us test your urine for certain drugs, including illegal drugs (for example cocaine and marijuana). If your urine shows you have taken any of these drugs, and they are not prescribed medications, you cannot be in the study. The results of the urine drug test won't become part of your medical record. These test results will, however, remain part of your study record. We will ask you if we can share this information with your parent, however if you choose not to share, we must keep this information confidential. If you are uncomfortable with drug testing or discussing drug use, then we would recommend that you not participate in this study. Throughout the study, if you are in immediate danger of hurting yourself or someone else, then we will notify your parent and refer you for an emergency evaluation.
    b. The vital signs may be collected by the clinical desk staff at the time of appointment check-in and may be recorded in your electronic health record (EHR).
7. Test your urine for pregnancy if you are a female able to become pregnant. Your parent/guardian will not be told the results of the pregnancy test without your permission. But, if your doctor believes that being pregnant may cause serious health problems, they may need to tell your parent/guardian the pregnancy test results.
8. Ask a series of safety-related questions that will help us make sure the study is safe for you. These include the following:

    TASS (Keel Transcranial Magnetic Stimulation Adult Safety Screen). This is a tool to help identify any potential safety problems related to TMS.

<u>Baseline Visit</u>

The Baseline Visit can take up to 4 hours. This visit will include the following:

1. You and your parents will complete a Pre/Post-Treatment Expectations and Experience Questionnaire (PRE-TEEQ-A/PRE-TEEQ-P and POST-TEEQ-A/POST-TEEQ-P) that asks questions about your initial expectations of this study and your final thoughts of this study.
2. Ask you a series of questions that tell us more about your mood at the start of the study. These include the following forms:
    - BDI-II
    - CDRS-R
    - CGI-S
    - C-SSRS
3. Ask you a series of questions that will help us make sure that the study activities are safe for you. These include the following:
    - Neuropsychological cognitive assessments (with the NIH Toolbox Cognition Battery)
    - SIT-R3 (Slosson Intelligence Test-Revised) Only if deemed medically necessary
    - YMRS (Young Mania Rating Scale)
    - Pediatric Adverse Event Rating Scale
    - Physical Symptom Checklist
4. You will have a hearing test. This will occur again after your treatment is complete. It is unlikely that the TMS procedures in the study will impact your hearing, but the study team is collecting safety information regarding hearing.
5. You will provide information about medications you have taken and/or are currently taking.
6. TMS testing of brain function involves placing a TMS coil on the scalp and stimulating either one time (single-pulse) or two times (paired-pulse) very quickly every few seconds over the area of your brain that controls the movement of your right thumb. The duration of each single-pulse or paired-pulse is less than 1 second. The study team will collect information about how much your thumb moves during the measures. The study team members will complete rating scales and brain activity measures with TMS, Electromyography (EMG), and electroencephalography (EEG). The EMG measures your muscle movement in your hand and the EEG measures your brain waves.

During this test:

- You will be given earplugs to wear (you will always have to wear earplugs with any kind of TMS in the study).
- The study team will attach electromyography (EMG) leads to your thumb and hand. The EMG leads have small pads that stick to your skin and send information to the computer. This is a way to measure how much your muscle moves. You will also wear a head net with EEG electrodes.
- The coil of the magnetic stimulator (Magstim 200) will be placed on your scalp and your scalp will be stimulated every few seconds to find the area of your brain that controls movement of your right thumb. The study team will also stimulate the prefrontal cortex. This will not make your thumb move but allows for brain wave measures. Each pulse is less than one second.
- Once the correct area on your head has been identified, the study doctor will place a small mark on your scalp using a felt tip marker. You will wear a swim cap if you prefer so that the felt tip marker does not directly touch your scalp.
- At this time, single and paired-pulse TMS measures will be collected. The doctor will again place the stimulator coil on the scalp, and use the mark on your scalp to as a guide for where to place the coil. You will hear clicking and may feel tapping sensations on your scalp.
- These TMS, EMG, EEG measures will be repeated after the 10 days of treatment.
- Some people may experience pain or discomfort during TMS, EMG, or EEG procedures.

For most people TMS does not hurt at all. Please let the study team know if you are uncomfortable or have pain at any time. TMS measures will be stopped immediately.

7. Before administering TMS treatment (with the MagVenture Stimulator), your specific treatment dose will be determined by finding your “Motor Threshold.” First, you will be seated in the treatment chair, the doctor or technician will place the active treatment coil against your head and will ensure a secure placement of the treatment coil against the left side of your head. You will be wearing earplugs. You will be asked to relax your right arm on the chair arm. When the TMS therapy is started you will again hear clicking and may feel tapping sensations on your scalp. The device power will then be adjusted to give just enough energy to send electromagnetic pulses through your scalp to make your hand twitch. The amount of energy required to make your hand twitch is called the “Motor Threshold.” Everyone has a different motor threshold and the treatments are given at an energy level that is just above the individual’s motor threshold.

Before you begin the treatment visits, you will be assigned by chance (like flipping a coin) to either active accelerated sequential bilateral theta burst stimulation or a sham comparison group. You and the Principal Investigator can't choose your study group. You will have an equal chance of being assigned to the active accelerated sequential bilateral theta burst stimulation or a sham comparison group. You will not be told which type of stimulation you are receiving. This is called "blinded."

Talk therapy will be provided as part of the study treatment so any other talk therapy with non-study providers must be discontinued during the 10 days of treatment

<u>Treatment Visits</u>
The TMS sessions in this study will involve stimulations on both the right and left scalp three times a day for 10 days. These visits will take up to 5 hours daily.

The treatment visits will occur daily, Monday through Friday. You will receive 3 daily treatments that last about 12 minutes each daily for a total of 30 sessions over 10 days. At each treatment visit we will:

1. Ask you a few questions about symptoms of your illness, changes to your medications, or any problems you may be having with treatment. These questions will include how you are feeling, if you have any physical symptoms, if you are struggling to sleep, and if any of your medications have changes. We will also ask about any recent alcohol or street drug use. This will only take about 5 to 10 minutes each time.
2. Help you get ready for your treatment; you will be asked to insert ear plugs into both ears in order to decrease the sound of the TMS treatment when the treatment coil is pulsing. Your treater will also wear ear plugs but still will be able to communicate with you.
3. The treatment coil will be moved to the treatment location on the right and then left side of your head. You will sit in the treatment chair while you receive the treatment that will last about 12 minutes. You may experience mild discomfort at the place where the coil is placed. The 3 daily treatments will be given to you with 1 hour breaks in between. This is for your comfort and to get the full benefit of the treatment.
4. The following measures will be repeated each day.
    - CDRS-R
    - C-SSRS
    - CGI-S
    - YMRS

During the daily treatments you will take part in talk therapy sessions focused on improving crisis skills and the management of suicidal thoughts.

Assessments after 10 days of treatment

After every week of rTMS treatment, the following assessments will be repeated. This process will take approximately 2 hours (in addition to your treatment sessions that day) to complete. You will also be asked to:

- CDRS-R
- C-SSRS
- CGI-S
- Physical Symptom Checklist
- Pediatric Adverse Event Rating Scale
- We will ask you and your parents to both complete the Pre/Post-TEEQ-A and Pre/Post-TEEQ-P.
- Have your vital signs taken
- Have a hearing test done
- Complete neuropsychological assessments (with the NIH Toolbox Cognition Battery)
- BDI-II
- Repeat the Pediatric Adverse Event Rating Scale
- YMRS

Monthly Follow Up Visits for 12 months

- CDRS-R
- BDI-II
- C-SSRS
- CGI-S
- YMRS
- Pediatric Adverse Event Rating Scale
- The study team will collect clinical information on your current psychotherapy treatment, concurrent medications, adverse events, emergency medicine department visits, hospitalizations, and suicide attempts

If the Principal Investigator feels that there is a clinically relevant research result this will be shared with you and you will be provided with documentation. The brain tests with TMS, EMG, EEG will not provide clinical findings. Please ask the Principal Investigator if you have any questions during study participation.

Mandated Reporting

In accordance with Minnesota State Law, all healthcare providers in the state are considered mandated reporters and are therefore required to report any actual or suspected physical, emotional, or sexual abuse of minors having occurred within the last 3 years.

If abuse is reported, a study doctor will meet with you and offer clinical referrals and contact information for additional resources to provide support. If you report that you have abused a child or elder, this abuse must also be reported.

What are the possible risks or discomforts from being in this research study?

The most severe known risks of TMS therapy are seizures and cardiogenic syncope (fainting).

You will be asked about several things which represent potential risks to your health when receiving TMS Therapy and that will prevent your participation in this study. These include:

- A history of bipolar disorder (manic-depressive illness) or eating disorders.
- A history of a neurological disorder, including brain tumors, seizures, stroke, abnormalities in the blood vessels in your brain, dementia, Parkinson’s disease, Huntington’s chorea or multiple sclerosis.
- Anything which could increase your risk of having a seizure, including a history of a head trauma with a loss of consciousness for more than 5 minutes, or the current use of certain medications.
- The presence in your body of cardiac pacemakers, implanted medication pumps of any sort, or a history of heart disease.
- The presence of any metal objects in or near your head which cannot be safely removed for the duration of this study.

Before TMS treatment, if you are taking an antipsychotic, stimulant medication, or some antidepressants (called tricyclic antidepressants) these will be stopped as these medications could increase your risk for seizures. There can be discontinuation symptoms associated with stopping these medications. These symptoms could include changes in mood, increased suicidal thoughts, increased suicidal behaviors, psychotic symptoms, changes in energy level, decreased focus, changes in appetite, and an overall worsening in your clinical condition that requires re-stabilization.

During the TMS treatments, you may experience buzzing, tapping, or painful sensations at the treatment site during the stimulations. These are usually mild to moderate in intensity and may become more tolerable after the first week of treatment. Not all patients experience these effects with TMS treatment.

Approval Date: November 10, 2022
Not to be used after: November 9, 2023

Common side effects of TMS include the following

- Pain or discomfort under the treatment coil
- Dizziness
- Eye pain
- Facial pain
- Toothache pain
- Skin pain
- Facial muscle twitching
- Blurred vision
- Feeling tired during and after treatments due to sedation

Not all patients experienced one of these side effects and, when present, it was generally mild to moderate in intensity and tended to decrease after the first weeks of treatment. The physician or treater may reposition the coil to try to resolve these side effects or the physician may recommend that you take an over-the-counter pain reliever such as acetaminophen (Tylenol) or ibuprofen (Motrin or Advil) before a treatment to decrease these sensations.

Temporary numbness of the face that lasted for 5 weeks after treatment with TMS but then resolved was reported in one patient in clinical trials using TMS therapy.
There may be other risks that are currently unknown.

Since FDA clearance TMS for depression, the seizure risk is ≤0.1% per patient (less than 1 in 1000 patients). In the event that you have a seizure, the study staff will immediately stop the treatment session and make sure that you are safe for the duration of the seizure. You will be observed for a period of time after the seizure to make sure you are feeling well, and someone will be asked to drive you home that day. Having a seizure includes a potential effect on your future employability, insurability, and ability to drive. Should you experience a seizure that is related to magnetic stimulation, your doctor will provide you with a letter stating that the seizure was produced under experimental conditions and that there is no reason to expect another occurrence.

Although the MagVenture stimulator makes less noise than other stimulators, you will wear protective ear plugs during treatment.

There is also a risk that treatment with TMS could lead to worsening of depression and induction of mania. Your existing mood disorder symptoms could become worse or you could develop new symptoms. The study team staff and psychiatrists will monitor for this carefully each day of treatment.

The effect on pregnancy and the unborn fetus are unknown; therefore pregnant subjects are excluded from the study. Females of child bearing potential who are capable of becoming pregnant must use a medically acceptable birth control method during the trial.

Other Risks Associated with the Study

You may have tattoos or piercings that are magnetic. Because of the electromagnetic pulse, these might feel warm or hot during the treatment.

Treatment with the MagVenture TMS Therapy System may involve other risks that are not known at the present time. The long-term effects of TMS are not known.

As with all research, there is a chance that confidentiality could be compromised; however, we take precautions to minimize this risk.

During this study, we will ask you to fill out questionnaires. We hope that you will answer all of the questions, but you can skip any questions you don't want to answer. The questionnaires will take about 20 minutes at each visit to complete.

Are there reasons you might leave this research study early?

You can decide to stop the study at any time. You should tell the Principal Investigator if you decide to stop, and you will be advised whether any additional tests may need to be done for your safety.

In addition, the Principal Investigator or Mayo Clinic may stop you from taking part in this study at any time:

- If it is in your best interest,
- If you don't follow the study procedures, or
- If the study is stopped for any reason.

If you leave this research study early, or are withdrawn from the study, no more information about you will be collected; however, information already collected about you in the study will continue to be used.

We will tell you about any new information that may affect your willingness to stay in the research study.

If you decide to stop taking part in the study for any reason, we will ask you to make a Withdrawal Study Visit. The visit will take up to 3 hours. At this visit, we will:

1. Ask you a series of questions that will tell us more about your mood at the end of the study. These include the following:
    - BDI-II
    - CDRS-R
    - CGI-S
    - C-SSRS
2. Ask you (and your parents) to complete the Pre/Post-TEEQ-A and Pre/Post-TEEQ-P (as applicable).
3. Complete Neuropsychological cognitive assessment (with the NIH Toolbox Cognition Battery)
4. Have a hearing test
5. Have your vital signs taken

What if you are injured from your participation in this research study?

Where to get help:

If you think you have suffered a research-related injury, you should promptly notify the Principal Investigator listed in the Contact Information at the beginning of this form. Mayo Clinic will offer care for research-related injuries, including first aid, emergency treatment and follow-up care as needed.

Who will pay for the treatment of research related injuries?

Care for such research-related injuries will be billed in the ordinary manner, to you or your insurance. You will be responsible for all treatment costs not covered by your insurance, including deductibles, co-payments and coinsurance.

The device manufacturer does not agree to reimburse for treatment of any research related injuries.

What are the possible benefits from being in this research study?

This study may not make your health better. However, you may find that this treatment helps your symptoms of depression and you may not need the other forms of treatment, including medication, or electroconvulsive therapy (ECT) and the associated general anesthesia with that form of treatment.

Your participation in this study may help researchers to determine if TMS can treat suicidal ideation in adolescents who have depression.

What alternative do you have if you choose not to participate in this research study?

You do not have to be in this study to receive treatment for your condition. Your other choices may include continuing your already-prescribed treatments for depression, such as talk therapy or medication therapy. You should talk to the researcher and your regular physician about each of your choices before you decide if you will take part in this study.

What tests or procedures will you need to pay for if you take part in this research study?

You will not need to pay for tests and procedures which are done just for this research study. These tests and procedures are:

- Urine pregnancy test (for women who may become pregnant)
- Urine drug screen
- Hearing screen
- Study related talk therapy
- TMS, EMG, and EEG measures of brain function
- TMS treatments

However, you and/or your health plan will need to pay for all other tests and procedures that you would normally have as part of your regular clinical care.

If you have billing or insurance questions call Patient Account Services at the telephone number provided in the Contact Information section of this form.

Will you be paid for taking part in this research study?

You (the participant) will be paid $50 for the baseline assessment day, $10 for treatment days, $50 for the post-treatment day, and $10 for each monthly follow up visit for a total of $320.

Payment for participation in research is considered taxable income and reportable to the Internal Revenue Service (IRS). Accounts Payable at Mayo Clinic will be given your name, address and Social Security number in order to issue a check for your study participation. If you receive research payments totaling $600 or more in a calendar year, a tax Form 1099 will be sent to you. For Mayo Clinic employees, research payments are included in your paycheck with applicable taxes withheld and reported on your Form W2 after calendar year-end.

Will your information or samples be used for future research?

Identifiable information such as your name, Mayo Clinic number, or date of birth may be removed from your information or samples collected in this study, allowing the information to be used for future research or shared with other researchers without your additional informed consent (and your child's assent).

Data from this study will be submitted to the National Institute of Mental Health Database (NDA) at the National Institutes of Health (NIH). NDA is a large database where deidentified study data from many National Institute of Mental Health (NIMH) studies is stored and managed. Deidentified study data means that all personal information about you (such as name, address, birthdate and phone number) is removed and replaced with a code number. Sharing your deidentified study data helps researchers learn new and important things about mental health and substance use more quickly than before.

During and after the study, the study researchers will send deidentified study data about your health and behavior to the NDA. Other researchers across the world can then request your deidentified study data for other research. Every researcher (and institutions to which they belong) who requests your deidentified study data must promise to keep your data safe and promise not to try to learn your identity. Experts at the NIH who know how to keep your data safe will review each request carefully to reduce risks to your privacy.

Sharing your study data does have some risks, although these risks are rare. Your study data could be accidentally shared with an unauthorized person who may attempt to learn your identity. The study researchers will make every attempt to protect your identity.

You may not benefit directly from allowing your study data to be shared with NDA. The study data provided to NDA may help researchers around the world learn more about mental health and substance use and how to help others who have problems with mental health and substance use. NIMH will also report to Congress and on its website about the different studies using NDA data. You will not be contacted directly about the study data you contributed to NDA.

You may decide now or later that you do not want your study data to be added to the NDA. You can still participate in this research study even if you decide that you do not want your data to be added to the NDA. If you decide any time after today that you do not want your data to be added to the NDA, call or email the study staff who conducted this study, and they will tell NDA to stop sharing your study data. Once your data is part of the NDA, the study researchers cannot take back the study data that was shared before they were notified that you changed your mind. If you would like more information about NDA, this is available on-line at http://nda.nih.gov.

I permit the investigators to send my deidentified data to the National Institute of Mental Health Database (NDA)

☐ Yes ☐ No Please initial here: ______ __Date: ________

How will your privacy and the confidentiality of your records be protected?

Mayo Clinic is committed to protecting the confidentiality of information obtained about you in connection with this research study.

Some of the information we collect about you for the study will be stored in your Mayo Clinic electronic medical record. Access to your electronic medical record is password-protected and persons accessing these records are monitored carefully.

The rest of the information we collect about you for the study will be kept in our study case file. These documents will only refer to you by the study number assigned to you.

During this research, information about your health will be collected. Under Federal law called the Privacy Rule, health information is private. However, there are exceptions to this rule, and you should know who may be able to see, use and share your health information for research and

why they may need to do so. Information about you and your health cannot be used in this research study without your written permission. If you sign this form, it will provide that permission (or “authorization”) to Mayo Clinic.

This research is covered by a Certificate of Confidentiality from the NIH. This means that the researchers cannot release or use information, documents, or samples that may identify you in any action or suit unless you say it is okay. They also cannot provide them as evidence unless you have agreed. This protection includes federal, state, or local civil, criminal, administrative, legislative, or other proceedings. An example would be a court subpoena.

The Certificate does not stop reporting that federal, state or local laws require. Some examples are laws that require reporting of child or elder abuse, some communicable diseases, and threats to harm yourself or others. The certificate cannot be used to stop a sponsoring United States federal or state government agency from checking records or evaluating programs. The certificate does not stop disclosures required by the federal Food and Drug Administration (FDA). The Certificate also does not prevent your information from being used for other research if allowed by federal regulations.

Researchers may release information about you when you say it is okay. For example, you may give them permission to release information to insurers, medical providers or any other persons not connected with the research. The Certificate of Confidentiality does not stop you from willingly releasing information about your involvement in this research. It also does not prevent you from having access to your own information.

Your health information may be collected from:

- Past, present and future medical records.
- Research procedures, including research office visits, tests, interviews and questionnaires.

Your health information will be used and/or given to others to:

- Do the research.
- Report the results.
- See if the research was conducted following the approved study plan, and applicable rules and regulations.

Your health information may be used and shared with:

- Mayo Clinic research staff involved in this study.
- Other Mayo Clinic staff involved in your clinical care.
- The sponsor(s) of this study and the people or groups hired by the sponsor(s) to help perform this research.
- The Mayo Clinic Institutional Review Board that oversees the research.
- Federal and State agencies (such as the Food and Drug Administration, the Department of Health and Human Services, the National Institutes of Health and other United States agencies) or government agencies in other countries that oversee or review research.
- The Data Safety Monitoring Board (DSMB). This is a group that oversees the data (study information) and safety of this research

How your information may be shared with others:

While taking part in this study, you will be assigned a code that is unique to you, but does not include information that directly identifies you. This code will be used if your study information is sent outside of Mayo Clinic. The groups or individuals who receive your coded information will use it only for the purposes described in this consent and assent form.

If the results of this study are made public (for example, through scientific meetings, reports or media), information that identifies you will not be used.

In addition, individuals involved in study oversight and <u>not</u> employed by Mayo Clinic may be allowed to review your health information included in past, present, and future medical and/or research records. This review may be done on-site at Mayo Clinic or remotely (from an off-site location). These records contain information that directly identifies you. However, the individuals will not be allowed to record, print, or copy (using paper, digital, photographic or other methods), or remove your identifying information from Mayo Clinic.

Is your health information protected after it has been shared with others?

Mayo Clinic asks anyone who receives your health information from us to protect your privacy; however, once your information is shared outside Mayo Clinic, we cannot promise that it will remain private and it may no longer be protected by the Privacy Rule.

Your Rights and Permissions

Participation in this study is completely voluntary. You have the right not to participate at all. Even if you decide to be part of the study now, you may change your mind and stop at any time. You do not have to sign this form, but if you do not, you cannot take part in this research study.

Deciding not to participate or choosing to leave the study will not result in any penalty. Saying 'no' will not harm your relationship with your own doctors or with Mayo Clinic.

If you cancel your permission for Mayo Clinic to use or share your health information, your participation in this study will end and no more information about you will be collected; however, information already collected about you in the study may continue to be used.

You can cancel your permission for Mayo Clinic to use or share your health information at any time by sending a letter to the address below:

Mayo Clinic
Office for Human Research Protection
ATTN: Notice of Revocation of Authorization
201 Building 4-60
200 1st Street SW
Rochester, MN 55905

Alternatively, you may cancel your permission by emailing the Mayo Clinic Research Participant Advocate at: researchparticipantadvocate@mayo.edu.

Please be sure to include in your letter or email:

- The name of the Principal Investigator,
- The study IRB number and /or study name, and
- Your contact information.

Your permission for Mayo Clinic to use and share your health information lasts until the end of this study, unless you cancel it. The study does not end until all data has been collected, checked (or audited), analyzed, and reported. Because research is an ongoing process, we cannot give you an exact date when the study will end. Sometimes this can be years after your study visits and/or activities have ended.

You may decide now or later that you do not want your study data to be added to the NDA. You can still participate in this research study even if you decide that you do not want your data to be added to the NDA. If you know now that you do not want your data in the NDA, please tell the study researcher before leaving the clinic today. If you decide any time after today that you do not want your data to be added to the NDA, call or email the study staff who conducted this study, and they will tell NDA to stop sharing your study data. Once your data is part of the NDA, the study researchers cannot take back the study data that was shared before they were notified that you changed your mind. If you would like more information about NDA, this is available on-line at http://nda.nih.gov.

MAYO CLINIC

Name and Clinic Number

Approval Date: November 10, 2022
Not to be used after: November 9, 2023

Enrollment and Permission Signatures

Your signature documents your permission to take part in this research. Signature of Child (ages 12-17):

Not applicable for informational purposes only / / : AM/PM

Printed Name Date Time

Not applicable for informational purposes only

Signature

Signature of Parent(s)/Guardian for Child:
I give permission for my child to take part in this research study and agree to allow his/her health information to be used and shared as described above.

Not applicable for informational purposes only

Printed Name of Child

Not applicable for informational purposes only / / : AM/PM

Printed Name of Parent or Guardian Date Time

Not applicable for informational purposes only

Signature

Name and Clinic Number

Approval Date: November 10, 2022
Not to be used after: November 9, 2023

Person Obtaining Consent

- I have explained the research study to the participant.
- I have answered all questions about this research study to the best of my ability.

Not applicable for informational purposes only / / : AM/PM

Printed Name Date Time

Not applicable for informational purposes only

Signature

MAYO
CLINIC

Name and Clinic Number

Approval Date: **November 10, 2022**
Not to be used after: **November 9, 2023**

FOR INFORMATIONAL PURPOSES ONLY

RESEARCH PARTICIPANT CONSENT AND PRIVACY AUTHORIZATION FORM

Study Title: A Randomized Controlled Trial of Sequential Bilateral Accelerated Theta Burst Stimulation in Adolescents with Suicidal Ideation Associated with Major Depressive Disorder (18-year-olds)

IRB#: 20-009630

Principal Investigator: Dr. Paul Croarkin and Colleagues

Key Study Information

This section provides a brief summary of the study. It is important for you to understand why the research is being done and what it will involve before you decide. **Please take the time to read the entire consent form carefully and talk to a member of the research team before making your decision.** You should not sign this form if you have any questions that have not been answered.	
**It's Your Choice**	This is a research study. Being in this research study is your choice; you do not have to participate. If you decide to join, you can still stop at any time. You should only participate if you want to do so. You will not lose any services, benefits or rights you would normally have if you choose not to take part.
**Research Purpose**	The purpose of this research is to conduct the first study of an investigational treatment; this means it is a pilot study using an investigational device. The investigational treatment is called sequential bilateral accelerated theta burst stimulation. Sequential bilateral accelerated theta burst stimulation is a new type of repetitive transcranial magnetic stimulation (TMS) that is delivered to both sides of the brain three times a day for 10 days. Throughout this consent form the abbreviation "TMS" will be used to describe research treatment in the study (again the technical name is sequential bilateral accelerated theta burst stimulation). Sham stimulation looks like TMS but has less than 5% of the magnetic field of TMS and is not thought to have an active treatment effect.

MAYO CLINIC

Name and Clinic Number

Approval Date: **November 10, 2022**
Not to be used after: **November 9, 2023**

	You have been asked to take part in this research because you are an adolescent between the ages of 12 and 18 with suicidal ideation and major depressive disorder. The plan is to have about 80 people with similar symptoms take part in the study at Mayo Clinic.
**What's Involved**	Study participation involves baseline assessment forms, brain measures before and after treatment, and 10 days of treatment with TMS or a sham stimulation. During the 10 days of treatment all patients will participate in a talk therapy program focused on coping skills. After treatment, patients will have monthly follow up visits for one year for assessments. The talk therapy will be provided as part of the study so any other talk therapy with non-study providers must be discontinued during the 10 days of treatment with TMS. This means that you will need to discontinue contact with any other outpatient therapist for 2 weeks. During the study you will have a screening visit, baseline visit, and 10 days of treatment. Each of these visit days will take up to 5 hours so you should plan for this. (e.g., this means that you may miss some school, work, or extracurricular activities).
**Key Information**	Patients in this study will receive a new treatment form of TMS or a sham comparison while participating in talk therapy. Sometimes patients find this new treatment uncomfortable and in rare cases it gives patients a brief seizure. The alternative to participating in the study is to have standard visits with a psychiatrist or therapist.
**Learn More**	If you are interested in learning more about this study, read the rest of this form carefully. The information in this form will help you decide if you want to participate in this research or not. A member of our research team will talk with you about taking part in this study before you sign this form. If you have questions at any time, please ask us.

Making Your Decision

Taking part in research is your decision. Take your time to decide. Feel free to discuss the study with your family, friends, and healthcare provider before you make your decision. Taking part in this study is completely voluntary and you do not have to participate.

If you decide to take part in this research study, you will sign this consent form to show that you want to take part. We will give you either a printed or electronic copy of this form to keep. A copy of this form will be put in your medical record.

For purposes of this form, Mayo Clinic refers to Mayo Clinic in Arizona, Florida and Rochester, Minnesota; Mayo Clinic Health System; and all owned and affiliated clinics, hospitals, and entities.

Name and Clinic Number

Approval Date: November 10, 2022
Not to be used after: November 9, 2023

Contact Information

If you have questions about …	You can contact …
▪ Study tests and procedures<br>▪ Materials you receive<br>▪ Research-related appointments<br>▪ Research-related concern or complaint<br>▪ Research-related injuries or emergencies<br>▪ Withdrawing from the research study	**Principal Investigator:** Paul Croarkin, DO, MS<br>**Phone:** (507) 293-2557<br><br>**Study Team Contact:** Jeremy Weiss<br>**Phone:** (507) 293-5659<br><br>**Institution Name and Address:**<br>Mayo Clinic<br>Department of Psychiatry and Psychology<br>200 First Street SW<br>Rochester, MN 55905
▪ Rights of a research participant	**Mayo Clinic Institutional Review Board (IRB)**<br>**Phone:** (507) 266-4000<br>**Toll-Free:** (866) 273-4681
▪ Rights of a research participant<br>▪ Any research-related concern or complaint<br>▪ Use of your Protected Health Information<br>▪ Stopping your authorization to use your Protected Health Information<br>▪ Withdrawing from the research study	**Research Participant Advocate (RPA)**<br>**(The RPA is independent of the Study Team)**<br>**Phone:** (507) 266-9372<br>**Toll-Free:** (866) 273-4681<br><br>**E-mail:** researchparticipantadvocate@mayo.edu
▪ Billing or insurance related to this research study	**Patient Account Services**<br>**Toll-Free:** (844) 217-9591

Other Information:

A description of this clinical trial will be available on http://www.ClinicalTrials.gov, as required by U.S. Law. This Web site will not include information that can identify you. At most, the Web site will include a summary of the results. You can search this Web site at any time.

A description of this clinical trial will be available on the mayoclinic.org research study web site.

Why are you being asked to take part in this research study?

You are being asked to take part in this research study because you are an adolescent between the ages of 12 and 18 with suicidal ideation and Major Depressive Disorder. The plan is to have about 80 people with similar symptoms take part in this study at Mayo Clinic.

Why is this research study being done?

The purpose of this study is to gather information regarding the feasibility, safety, and benefits of a new form of TMS treatment for suicidal ideation in adolescents with Major Depressive Disorder. The investigators hope to learn if this TMS treatment improves suicidal ideation over 10 days and clinical outcomes over 1 year of follow-up. The investigators will also collect measures of brain function before and after TMS treatment.

Therapy with TMS has U.S. Food and Drug Administration (FDA) clearance for the treatment of adults with Major Depressive Disorder who have failed prior antidepressant medication therapy. Therapy with TMS for children and adolescents with Major Depressive Disorder has been studied previously but is considered investigational in this study. The FDA has granted permission to use the magnetic stimulator in this study (called MagVenture) under an Investigational Device Exemption (IDE).

Information you should know

Who is Funding the Study?
This study is funded by the National Institute of Mental Health (NIMH). The stimulators and supplies needed for the study treatments will be provided by MagVenture.

Information Regarding Conflict of Interest:
Your healthcare provider may be referring you to this research study. If your healthcare provider is also an investigator on this study, there is the chance that his or her responsibilities for the study could influence his or her recommendation for your participation.

If you prefer, your healthcare provider will be happy to refer you to another investigator on the research study team for you to decide if you want to participate in the study and to see you for the research study activities while you are in the study.

How long will you be in this research study?

There is a treatment part of the study that lasts 2 weeks. You then will be asked to follow-up with the study team once a month for 1 year. So if you are willing you will be in the research study for approximately 13 months.

What will happen to you while you are in this research study?

If you agree to be in the study, you will be asked to participate in the following:

<u>Screening Visit</u>
The Screening Visit will help us determine if you are eligible for the study and will take up to 5 hours. The Principal Investigator will review the results of these tests and procedures. If you are not eligible, the Principal Investigator will tell you why. At this visit, we will:

1. Review the informed consent form and answer any questions you have. You must sign the informed consent from prior to completing any study procedures. A signed copy will be provided to you for your records.
2. Ask about your psychiatric and medical history.
3. Review information about medications you have taken and/or are currently taking.
4. Discontinue any medications or treatments that are not allowed during the study.
5. Have an interview with a doctor and/or other study staff member to determine your diagnoses and level of depression. These questions help us make sure that the study is right for you.

   These will include the following forms:
   - M.I.N.I./M.I.N.I.KID (Min International Neuropsychiatric Interview for Children and Adolescents) is a short structured diagnostic interview for psychiatric disorders in children and adolescents.
   - BANPS (Brief Affective Neuroscience Personality Scale) is a 33-question assessment about your personality.
   - CDRS-R (Children's Depression Rating Scale, Revised) is a 17-question form that helps describe your level of depression. Parents help answer 14 of the 17 questions.
   - CTQ (Childhood Trauma Questionnaire) is a 28-question form that helps describe adversities you faced.

- BDI-II (Beck Depression Inventory II) is a 21-question form that explains your symptoms of depression.
- CGI (Clinical Global Impression) is a form the study team uses to rate your mood
- Columbia Suicide Severity Rating Scale (C-SSRS) is a question that assesses your suicidal ideation.
- YMRS (Young Mania Rating Scale) is a form that rates any symptoms of bipolar disorder you might have.
- You will complete a PDS (Pubertal Development Scale and Tanner Staging) form that (with line drawings and questions) determines how far along you are in puberty/
- A Demographics form that asks about your racial and gender identity, and education of your parents

6. Have a medical history collected, physical examination, collect vital signs (height, weight, blood pressure, and heart rate), and urine drug screen.
   a. To participate in the study you must let us test your urine for certain drugs, including illegal drugs (for example cocaine and marijuana). If your urine shows you have taken any of these drugs, and they are not prescribed medications, you cannot be in the study. The results of the urine drug test won't become part of your medical record. These test results will, however, remain part of your study record.
   b. The vital signs may be collected by the clinical desk staff at the time of appointment check-in and may be recorded in your electronic health record (EHR).
7. Test your urine for pregnancy if you are a female able to become pregnant.
8. Ask a series of safety-related questions that will help us make sure the study is safe for you. These include the following:

   TASS (Keel Transcranial Magnetic Stimulation Adult Safety Screen). This is a tool to help identify any potential safety problems related to TMS.

<u>Baseline Visit</u>
The Baseline Visit can take up to 4 hours. This visit will include the following:

1. You and your parents will complete a Pre/Post-Treatment Expectations and Experience Questionnaire (PRE-TEEQ-A/PRE-TEEQ-P and POST-TEEQ-A/POST-TEEQ-P) that asks questions about your initial expectations of this study and your final thoughts of this study.

2. Ask you a series of questions that tell us more about your mood at the start of the study. These include the following forms:
    - BDI-II
    - CDRS-R
    - CGI-S
    - C-SSRS
3. Ask you a series of questions that will help us make sure that the study activities are safe for you. These include the following:
    - Neuropsychological cognitive assessments (with the NIH Toolbox Cognition Battery)
    - SIT-R3 (Slosson Intelligence Test-Revised) Only if deemed medically necessary
    - YMRS (Young Mania Rating Scale)
    - Pediatric Adverse Event Rating Scale
    - Physical Symptom Checklist
4. You will have a hearing test. This will occur again after your treatment is complete. It is unlikely that the TMS procedures in the study will impact your hearing, but the study team is collecting safety information regarding hearing.
5. You will provide information about medications you have taken and/or are currently taking.
6. TMS testing of brain function involves placing a TMS coil on the scalp and stimulating either one time (single-pulse) or two times (paired-pulse) very quickly every few seconds over the area of your brain that controls the movement of your right thumb. The duration of each single-pulse or paired-pulse is less than 1 second. The study team will collect information about how much your thumb moves during the measures. The study team members will complete rating scales and brain activity measures with TMS, Electromyography (EMG), and electroencephalography (EEG). The EMG measures your muscle movement in your hand and the EEG measures your brain waves.

During this test:

- You will be given earplugs to wear (you will always have to wear earplugs with any kind of TMS in the study).
- The study team will attach electromyography (EMG) leads to your thumb and hand. The EMG leads have small pads that stick to your skin and send information to the computer. This is a painless way to measure how much your muscle moves. You will also wear a head net with EEG electrodes.
- The coil of the magnetic stimulator (Magstim 200) will be placed on your scalp and your scalp will be stimulated every few seconds to find the area of your brain that controls movement of your right thumb. The study team will also stimulate the prefrontal cortex. This will not make your thumb move but allows for brain wave measures. Each pulse is less than one second.

- Once the correct area on your head has been identified, the study doctor will place a small mark on your scalp using a felt tip marker. You will wear a swim cap if you prefer so that the felt tip marker does not directly touch your scalp.
- At this time, single and paired-pulse TMS measures will be collected. The doctor will again place the stimulator coil on the scalp, and use the mark on your scalp to as a guide for where to place the coil. You will hear clicking and may feel tapping sensations on your scalp.
- These TMS, EMG, EEG measures will be repeated after the 10 days of treatment.
- Some people may experience pain or discomfort during TMS, EMG, or EEG procedures.

For most people TMS does not hurt at all. Please let the study team know if you are uncomfortable or have pain at any time. TMS measures will be stopped immediately.

7. Before administering TMS treatment (with the MagVenture Stimulator), your specific treatment dose will be determined by finding your "Motor Threshold." First, you will be seated in the treatment chair, the doctor or technician will place the active treatment coil against your head and will ensure a secure placement of the treatment coil against the left side of your head. You will be wearing earplugs. You will be asked to relax your right arm on the chair arm. When the TMS therapy is started you will again hear clicking and may feel tapping sensations on your scalp. The device power will then be adjusted to give just enough energy to send electromagnetic pulses through your scalp to make your hand twitch. The amount of energy required to make your hand twitch is called the "Motor Threshold." Everyone has a different motor threshold and the treatments are given at an energy level that is just above the individual's motor threshold.

Before you begin the treatment visits, you will be assigned by chance (like flipping a coin) to either active accelerated sequential bilateral theta burst stimulation or a sham comparison group. You and the Principal Investigator can't choose your study group.

You will have an equal chance of being assigned to the active accelerated sequential bilateral theta burst stimulation or a sham comparison group. You will not be told which type of stimulation you are receiving. This is called "blinded."

Talk therapy will be provided as part of the study treatment so any other talk therapy with non-study providers must be discontinued during the 10 days of treatment.

<u>Treatment Visits</u>
The TMS sessions in this study will involve stimulations on both the right and left scalp three times a day for 10 days. These visits will take up to 5 hours daily.

The treatment visits will occur daily, Monday through Friday. You will receive 3 daily treatments that last about 12 minutes each daily for a total of 30 sessions over 10 days. At each treatment visit we will:

1. Ask you a few questions about symptoms of your illness, changes to your medications, or any problems you may be having with treatment. These questions will include how you are feeling, if you have any physical symptoms, if you are struggling to sleep, and if any of your medications have changes. We will also ask about any recent alcohol or street drug use. This will only take about 5 to 10 minutes each time.
2. Help you get ready for your treatment; you will be asked to insert ear plugs into both ears in order to decrease the sound of the TMS treatment when the treatment coil is pulsing. Your treater will also wear ear plugs but still will be able to communicate with you.
3. The treatment coil will be moved to the treatment location on the right and then left side of your head. You will sit in the treatment chair while you receive the treatment that will last about 12 minutes. You may experience mild discomfort at the place where the coil is placed. The 3 daily treatments will be given to you with 1 hour breaks in between. This is for your comfort and to get the full benefit of the treatment.
4. The following measures will be repeated each day.
    - CDRS-R
    - C-SSRS
    - CGI-S
    - YMRS

During the daily treatments you will take part in talk therapy sessions focused on improving crisis skills and the management of suicidal thoughts.

<u>Assessments after 10 days of treatment</u>
After every week of rTMS treatment, the following assessments will be repeated. This process will take approximately 2 hours (in addition to your treatment sessions that day) to complete.

You will also be asked to:

- CDRS-R
- C-SSRS
- CGI-S
- Physical Symptom Checklist
- Pediatric Adverse Event Rating Scale
- We will ask you and your parents to both complete the Pre/Post-TEEQ-A and Pre/Post-TEEQ-P.
- Have your vital signs taken
- Have a hearing test done
- Complete neuropsychological assessments (with the NIH Toolbox Cognition Battery)

- BDI-II
- Repeat the Pediatric Adverse Event Rating Scale
- YMRS

Monthly Follow Up Visits for 12 months

- CDRS-R
- BDI-II
- C-SSRS
- CGI-S
- YMRS
- Pediatric Adverse Event Rating Scale
- The study team will collect clinical information on your current psychotherapy treatment, concurrent medications, adverse events, emergency medicine department visits, hospitalizations, and suicide attempts

If the Principal Investigator feels that there is a clinically relevant research result this will be shared with you and you will be provided with documentation. The brain tests with TMS, EMG, EEG will not provide clinical findings. Please ask the Principal Investigator if you have any questions during study participation.

Mandated Reporting

In accordance with Minnesota State Law, all healthcare providers in the state are considered mandated reporters and are therefore required to report any actual or suspected physical, emotional, or sexual abuse of minors having occurred within the last 3 years.

If abuse is reported, a study doctor will meet with you and offer clinical referrals and contact information for additional resources to provide support. If you report that you have abused a child or elder, this abuse must also be reported.

What are the possible risks or discomforts from being in this research study?

The most severe known risks of TMS therapy are seizures and cardiogenic syncope (fainting).

You will be asked about several things which represent potential risks to your health when receiving TMS Therapy and that will prevent your participation in this study. These include:

- A history of bipolar disorder (manic-depressive illness) or eating disorders.
- A history of a neurological disorder, including brain tumors, seizures, stroke, abnormalities in the blood vessels in your brain, dementia, Parkinson's disease, Huntington's chorea or multiple sclerosis.

- Anything which could increase your risk of having a seizure, including a history of a head trauma with a loss of consciousness for more than 5 minutes, or the current use of certain medications.
- The presence in your body of cardiac pacemakers, implanted medication pumps of any sort, or a history of heart disease.
- The presence of any metal objects in or near your head which cannot be safely removed for the duration of this study.

Before TMS treatment if you are taking an antipsychotic, stimulant medication, or some antidepressants (called tricyclic antidepressants) these will be stopped as these medications could increase your risk for seizures. Please note that there can be discontinuation symptoms associated with stopping these medications. These symptoms could include changes in mood, increased suicidal thoughts, increased suicidal behaviors, psychotic symptoms, changes in energy level, decreased focus, changes in appetite, and an overall worsening in your clinical condition that requires re-stabilization.

During the TMS treatments, you may experience buzzing, tapping, or painful sensations at the treatment site during the stimulations. These are usually mild to moderate in intensity and may become more tolerable after the first week of treatment. Not all patients experience these effects with TMS treatment.

Common side effects of TMS include the following:
- Pain or discomfort under the treatment coil
- Dizziness
- Eye pain
- Facial pain
- Toothache pain
- Skin pain
- Facial muscle twitching
- Blurred vision
- Feeling tired during and after treatments due to sedation

Not all patients experienced one of these side effects and, when present, it was generally mild to moderate in intensity and tended to decrease after the first weeks of treatment. The physician or treater may reposition the coil to try to resolve these side effects or the physician may recommend that you take an over-the-counter pain reliever such as acetaminophen (Tylenol) or ibuprofen (Motrin or Advil) before a treatment to decrease these sensations.

Temporary numbness of the face that lasted for 5 weeks after treatment with TMS but then resolved was reported in one patient in clinical trials using TMS therapy.
There may be other risks that are currently unknown.

Since FDA clearance TMS for depression, the seizure risk is ≤0.1% per patient (less than 1 in 1000 patients). In the event that you have a seizure, the study staff will immediately stop the treatment session and make sure that you are safe for the duration of the seizure. You will be observed for a period of time after the seizure to make sure you are feeling well, and someone will be asked to drive you home that day. Having a seizure includes a potential effect on your future employability, insurability, and ability to drive. Should you experience a seizure that is related to magnetic stimulation, your doctor will provide you with a letter stating that the seizure was produced under experimental conditions and that there is no reason to expect another occurrence.

Although the MagVenture stimulator makes less noise than other stimulators, you will wear protective ear plugs during treatment.

There is also a risk that treatment with TMS could lead to worsening of depression and induction of mania. Your existing mood disorder symptoms could become worse or you could develop new symptoms. The study team staff and psychiatrists will monitor for this carefully each day of treatment.

The effect on pregnancy and the unborn fetus are unknown; therefore pregnant subjects are excluded from the study. Females of child bearing potential who are capable of becoming pregnant must use a medically acceptable birth control method during the trial.

Other Risks Associated with the Study

You may have tattoos or piercings that are magnetic. Because of the electromagnetic pulse, these might feel warm or hot during the treatment.

Treatment with the MagVenture TMS Therapy System may involve other risks that are not known at the present time. The long-term effects of TMS are not known.

During this study, we will ask you to fill out questionnaires. We hope that you will answer all of the questions, but you can skip any questions you don't want to answer. The questionnaires will take about 20 minutes to complete.

As with all research, there is a chance that confidentiality could be compromised; however, we take precautions to minimize this risk.

Are there reasons you might leave this research study early?

You can decide to stop the study at any time. You should tell the Principal Investigator if you decide to stop, and you will be advised whether any additional tests may need to be done for your safety.

In addition, the Principal Investigator or Mayo Clinic may stop you from taking part in this study at any time:

- If it is in your best interest,
- If you don't follow the study procedures, or
- If the study is stopped for any reason.

If you leave this research study early, or are withdrawn from the study, no more information about you will be collected; however, information already collected about you in the study may continue to be used.

We will tell you about any new information that may affect your willingness to stay in the research study.

If you decide to stop taking part in the study for any reason, we will ask you to make a Withdrawal Study Visit. The visit will take up to 3 hours. At this visit, we will:

1. Ask you a series of questions that will tell us more about your mood at the end of the study. These include the following:
   - BDI-II
   - CDRS-R
   - CGI-S
   - C-SSRS
2. Ask you (and your parents) to complete the Pre/Post-TEEQ-A and Pre/Post-TEEQ-P (as applicable).
3. Complete Neuropsychological cognitive assessment (with the NIH Toolbox Cognition Battery)
4. Have a hearing test
5. Have your vital signs taken

What if you are injured from your participation in this research study?

Where to get help:
If you think you have suffered a research-related injury, you should promptly notify the Principal Investigator listed in the Contact Information at the beginning of this form. Mayo Clinic will offer care for research-related injuries, including first aid, emergency treatment and follow-up care as needed.

Who will pay for the treatment of research related injuries?
Care for such research-related injuries will be billed in the ordinary manner, to you or your insurance. You will be responsible for all treatment costs not covered by your insurance, including deductibles, co-payments and coinsurance.

The device manufacturer does not agree to reimburse for treatment of any research related injuries.

What are the possible benefits from being in this research study?

This study may not make your health better. However, you may find that this treatment helps your symptoms of depression and you may not need the other forms of treatment, including medication, or electroconvulsive therapy (ECT) and the associated general anesthesia with that form of treatment.

Your participation in this study may help researchers to determine if TMS can treat suicidal ideation in adolescents who have depression.

What alternative do you have if you choose not to participate in this research study?

You do not have to be in this study to receive treatment for your condition. Your other choices may include continuing your already-prescribed treatments for depression, such as talk therapy or medication therapy. You should talk to the researcher and your regular physician about each of your choices before you decide if you will take part in this study.

What tests or procedures will you need to pay for if you take part in this research study?

You will not need to pay for tests and procedures which are done just for this research study. These tests and procedures are:

- Urine pregnancy test (for women who may become pregnant)
- Urine drug screen
- Hearing screen
- Study related talk therapy
- TMS, EMG, and EEG measures of brain function
- TMS treatments

However, you and/or your health plan will need to pay for all other tests and procedures that you would normally have as part of your regular clinical care.

If you have billing or insurance questions call Patient Account Services at the telephone number provided in the Contact Information section of this form.

Will you be paid for taking part in this research study?

You will be paid $50 for the baseline assessment day, $10 for treatment days, $50 for the post-treatment day, and $10 for each monthly follow up visit for a total of $320.

Payment for participation in research is considered taxable income and reportable to the Internal Revenue Service (IRS). Accounts Payable at Mayo Clinic will be given your name, address and Social Security number in order to issue a check for your study participation. If you receive research payments totaling $600 or more in a calendar year, a tax Form 1099 will be sent to you. For Mayo Clinic employees, research payments are included in your paycheck with applicable taxes withheld and reported on your Form W2 after calendar year-end.

Will your information or samples be used for future research?

Identifiable information such as your name, Mayo Clinic number, or date of birth may be removed from your information or samples collected in this study, allowing the information or samples to be used for future research or shared with other researchers without your additional informed consent.

Data from this study will be submitted to the National Institute of Mental Health Database (NDA) at the National Institutes of Health (NIH). NDA is a large database where deidentified study data from many National Institute of Mental Health (NIMH) studies is stored and managed. Deidentified study data means that all personal information about you (such as name, address, birthdate and phone number) is removed and replaced with a code number. Sharing your deidentified study data helps researchers learn new and important things about mental health and substance use more quickly than before.

During and after the study, the study researchers will send deidentified study data about your health and behavior to the NDA. Other researchers across the world can then request your deidentified study data for other research. Every researcher (and institutions to which they belong) who requests your deidentified study data must promise to keep your data safe and promise not to try to learn your identity. Experts at the NIH who know how to keep your data safe will review each request carefully to reduce risks to your privacy.

Sharing your study data does have some risks, although these risks are rare. Your study data could be accidentally shared with an unauthorized person who may attempt to learn your identity. The study researchers will make every attempt to protect your identity.

You may not benefit directly from allowing your study data to be shared with NDA. The study data provided to NDA may help researchers around the world learn more about mental health and substance use and how to help others who have problems with mental health and substance use. NIMH will also report to Congress and on its website about the different studies using NDA data. You will not be contacted directly about the study data you contributed to NDA.

You may decide now or later that you do not want your study data to be added to the NDA. You can still participate in this research study even if you decide that you do not want your data to be added to the NDA. If you decide any time after today that you do not want your data to be added to the NDA, call or email the study staff who conducted this study, and they will tell NDA to stop sharing your study data. Once your data is part of the NDA, the study researchers cannot take back the study data that was shared before they were notified that you changed your mind. If you would like more information about NDA, this is available on-line at http://nda.nih.gov.

MAYO CLINIC

Name and Clinic Number

Approval Date: **November 10, 2022**
Not to be used after: **November 9, 2023**

I permit the investigators to send my deidentified data to the National Institute of Mental Health Database (NDA)

☐ Yes ☐ No Please initial here: ______ __Date: ________

How will your privacy and the confidentiality of your records be protected?

Mayo Clinic is committed to protecting the confidentiality of information obtained about you in connection with this research study.

Some of the information we collect about you for the study will be stored in your Mayo Clinic electronic medical record. Access to your electronic medical record is password-protected and persons accessing these records are monitored carefully.

The rest of the information we collect about you for the study will be kept in our study case file. These documents will only refer to you by the study number assigned to you.

During this research, information about your health will be collected. Under Federal law called the Privacy Rule, health information is private. However, there are exceptions to this rule, and you should know who may be able to see, use and share your health information for research and why they may need to do so. Information about you and your health cannot be used in this research study without your written permission. If you sign this form, it will provide that permission (or "authorization") to Mayo Clinic.

This research is covered by a Certificate of Confidentiality from the NIH. This means that the researchers cannot release or use information, documents, or samples that may identify you in any action or suit unless you say it is okay. They also cannot provide them as evidence unless you have agreed. This protection includes federal, state, or local civil, criminal, administrative, legislative, or other proceedings. An example would be a court subpoena.

The Certificate does not stop reporting that federal, state or local laws require. Some examples are laws that require reporting of child or elder abuse, some communicable diseases, and threats to harm yourself or others. The certificate cannot be used to stop a sponsoring United States federal or state government agency from checking records or evaluating programs. The certificate does not stop disclosures required by the federal Food and Drug Administration (FDA). The Certificate also does not prevent your information from being used for other research if allowed by federal regulations.

Researchers may release information about you when you say it is okay. For example, you may give them permission to release information to insurers, medical providers or any other persons not connected with the research. The Certificate of Confidentiality does not stop you from willingly releasing information about your involvement in this research. It also does not prevent you from having access to your own information.

Your health information may be collected from:

- Past, present and future medical records.
- Research procedures, including research office visits, tests, interviews and questionnaires.

Your health information will be used and/or given to others to:

- Do the research.
- Report the results.
- See if the research was conducted following the approved study plan, and applicable rules and regulations.

Your health information may be used and shared with:

- Mayo Clinic research staff involved in this study.
- Other Mayo Clinic staff involved in your clinical care.
- The sponsor(s) of this study and the people or groups hired by the sponsor(s) to help perform this research.
- The Mayo Clinic Institutional Review Board that oversees the research.
- Federal and State agencies (such as the Food and Drug Administration, the Department of Health and Human Services, the National Institutes of Health and other United States agencies) or government agencies in other countries that oversee or review research.
- The Data Safety Monitoring Board (DSMB). This is a group that oversees the data (study information) and safety of this research.

How your information may be shared with others:

While taking part in this study, you will be assigned a code that is unique to you, but does not include information that directly identifies you. This code will be used if your study information is sent outside of Mayo Clinic. The groups or individuals who receive your coded information will use it only for the purposes described in this consent form.

If the results of this study are made public (for example, through scientific meetings, reports or media), information that identifies you will not be used.

In addition, individuals involved in study oversight and not employed by Mayo Clinic may be allowed to review your health information included in past, present, and future medical and/or research records. This review may be done on-site at Mayo Clinic or remotely (from an off-site location). These records contain information that directly identifies you.
However, the individuals will not be allowed to record, print, or copy (using paper, digital, photographic or other methods), or remove your identifying information from Mayo Clinic.

Is your health information protected after it has been shared with others?

Mayo Clinic asks anyone who receives your health information from us to protect your privacy; however, once your information is shared outside Mayo Clinic, we cannot promise that it will remain private and it may no longer be protected by the Privacy Rule.

Your Rights and Permissions

Participation in this study is completely voluntary. You have the right not to participate at all. Even if you decide to be part of the study now, you may change your mind and stop at any time. You do not have to sign this form, but if you do not, you cannot take part in this research study.

Deciding not to participate or choosing to leave the study will not result in any penalty. Saying 'no' will not harm your relationship with your own doctors or with Mayo Clinic.

If you cancel your permission for Mayo Clinic to use or share your health information, your participation in this study will end and no more information about you will be collected; however, information already collected about you in the study may continue to be used.

You can cancel your permission for Mayo Clinic to use or share your health information at any time by sending a letter to the address below:

Mayo Clinic
Office for Human Research Protection
ATTN: Notice of Revocation of Authorization
201 Building 4-60
200 1st Street SW
Rochester, MN 55905

Alternatively, you may cancel your permission by emailing the Mayo Clinic Research Participant Advocate at: researchparticipantadvocate@mayo.edu.

Please be sure to include in your letter or email:

- The name of the Principal Investigator,
- The study IRB number and /or study name, and
- Your contact information.

Your permission for Mayo Clinic to use and share your health information lasts until the end of this study, unless you cancel it. The study does not end until all data has been collected, checked (or audited), analyzed, and reported. Because research is an ongoing process, we cannot give you an exact date when the study will end. Sometimes this can be years after your study visits and/or activities have ended.

You may decide now or later that you do not want your study data to be added to the NDA. You can still participate in this research study even if you decide that you do not want your data to be added to the NDA. If you know now that you do not want your data in the NDA, please tell the study researcher before leaving the clinic today. If you decide any time after today that you do not want your data to be added to the NDA, call or email the study staff who conducted this study, and they will tell NDA to stop sharing your study data. Once your data is part of the NDA, the study researchers cannot take back the study data that was shared before they were notified that you changed your mind. If you would like more information about NDA, this is available on-line at http://nda.nih.gov.

MAYO CLINIC

Name and Clinic Number

Approval Date: November 10, 2022
Not to be used after: November 9, 2023

Enrollment and Permission Signatures

Your signature documents your permission to take part in this research. Signature of Adult Subject (age 18):

Not applicable for informational purposes only / / : AM/PM

Printed Name Date Time

Not applicable for informational purposes only

Signature

Person Obtaining Consent

- I have explained the research study to the participant.
- I have answered all questions about this research study to the best of my ability.

Not applicable for informational purposes only / / : AM/PM

Printed Name Date Time

Not applicable for informational purposes only

Signature
