# Supplementary material for: Sequential bilateral accelerated theta burst stimulation in adolescents with suicidal ideation associated with major depressive disorder: Protocol for a randomized controlled trial
Source: PLoS One. 2023 Apr 13;18(4):e0280010. doi: 10.1371/journal.pone.0280010 (PMC10101506; doi:10.1371/journal.pone.0280010)
Supplement: S3 File — (PDF) [file pone.0280010.s004.pdf]

A RANDOMIZED CONTROLLED TRIAL OF SEQUENTIAL BILATERAL ACCELERATED THETA BURST STIMULATION IN ADOLESCENTS WITH SUICIDAL IDEATION ASSOCIATED WITH MAJOR DEPRESSIVE DISORDER

Principal Investigator: Paul E. Croarkin, DO, MS

Co-Investigator(s): Jarrod M. Leffler, PhD
Paul A. Nakonezny, PhD
Julia Shekunov, MD
Jennifer L. Vande Voort, MD
Michael J. Zaccariello, PhD

Mayo Clinic Depression Center
Department of Psychiatry and Psychology
200 First Street SW
Rochester, Minnesota 55905
(507) 293-2557

Funding Sponsor: National Institute of Mental Health (NIMH) Grant 1R01MH124655-01

Study Product: MagPro X100 with MagOption (K173620) and Cool-B70 A/P coil (investigational device), MagVenture

Protocol Number: (IRBe) 20-009630

IDE Number: G200220

IDE Sponsor: Paul E. Croarkin, D.O., M.S.

ClinicalTrials.gov Identifier: NCT04701840

Initial version:17 September 2020 Version **(1.0)**
Subsequent versions:
1 June 2021 Data and Safety Monitoring Board DSMB (Approved Version)

Table of Contents

STUDY SUMMARY **7**

1 INTRODUCTION **12**

1.1 BACKGROUND 12
1.2 INVESTIGATIONAL DEVICE 14
1.3 PRECLINICAL DATA 15
1.4 CLINICAL DATA TO DATE 16
1.5 STUDY RATIONALE AND RISK ANALYSIS (RISKS TO BENEFITS RATIO) 18
1.5.1 Study Rationale 18
1.5.2 Anticipated Risks 22
1.5.3 Potential Benefits 27
1.6 ANTICIPATED DURATION OF THE CLINICAL INVESTIGATION 28

2 STUDY OBJECTIVES **28**

3 STUDY DESIGN **30**

3.1 GENERAL DESIGN 30
3.2 PRIMARY STUDY ENDPOINTS 32
3.3 SECONDARY STUDY ENDPOINTS 32
3.4 PRIMARY SAFETY ENDPOINTS 32

4 SUBJECT SELECTION, ENROLLMENT AND WITHDRAWAL **33**

4.1 INCLUSION CRITERIA 33
4.2 EXCLUSION CRITERIA 33
4.3 SUBJECT RECRUITMENT, ENROLLMENT AND SCREENING 34
4.4 EARLY WITHDRAWAL OF SUBJECTS 36
4.4.1 When and How to Withdraw Subjects 36
4.4.2 Data Collection and Follow-up for Withdrawn Subjects 36

5 STUDY DEVICE **37**

5.1 DESCRIPTION 37
5.2 METHOD FOR ASSIGNING SUBJECTS TO TREATMENT GROUPS 41
5.3 PREPARATION AND ADMINISTRATION/IMPLANTATION OF INVESTIGATIONAL DEVICE 41
5.4 SUBJECT COMPLIANCE MONITORING 41
5.5 PRIOR AND CONCOMITANT THERAPY 41
5.6 PACKAGING AND LABELING 42
5.7 MASKING/BLINDING OF STUDY 42
5.8 RECEIVING, STORAGE, DISTRIBUTION AND RETURN 42
5.8.1 Receipt of Investigational Devices 42
5.8.2 Storage 42
5.8.3 Distribution of Study Device 43
5.8.4 Return or Destruction of Study Device 43

6 STUDY PROCEDURES **43**

6.1 VISIT 1 45
6.2 VISIT 2 46

7 STATISTICAL PLAN **50**

7.1 SAMPLE SIZE DETERMINATION 50
7.2 STATISTICAL METHODS 52
7.3 SUBJECT POPULATION(S) FOR ANALYSIS 55

8 SAFETY AND ADVERSE EVENTS **56**

8.1 DEFINITIONS 57

8.2 Recording of Adverse Events 60
8.3 Sponsor-Investigator Reporting of Unanticipated Adverse Device Effects and Unanticipated Problems 61
8.3.1 Sponsor-Investigator Reporting, Notifying Mayo IRB 61
8.3.2 Sponsor-Investigator Reporting: Notifying the FDA 61
8.4 Unblinding Procedures (Breaking the Blind) (as necessary if the study is blinded) 63
8.5 Stopping Rules 63
8.6 Medical Monitoring 64
8.6.1 Independent Data and Safety Monitoring Board 64

9 DATA HANDLING AND RECORD KEEPING 65

9.1 Confidentiality 65
9.2 Source Documents 65
9.3 Case Report Forms 65
9.4 Records Retention 66

10 STUDY MONITORING, AUDITING, AND INSPECTING 67

10.1 Study Monitoring Plan 67
10.2 Auditing and Inspecting 68

11 ETHICAL CONSIDERATIONS 68

12 STUDY FINANCES 69

12.1 Funding Source 69
12.2 Conflict of Interest 69
12.3 Subject Stipends or Payments 70

13 PUBLICATION PLAN 70

14 REFERENCES 70

LIST OF ABBREVIATIONS

AE	Adverse Event/Adverse Experience
ATHF	Antidepressant Treatment History Form
aTBS	Accelerated Theta Burst Stimulation
cTBS	Continuous Theta Burst Stimulation
BDI-II	Beck Depression Inventory-II
CCaTS	Center for Clinical and Translational Science
CDRS-R	Children’s Depression Rating Scale Revised
CFR	Code of Federal Regulations
CGI-S	Clinical Global Impression Severity Scale
CRF	Case Report Form
C-SSRS	Columbia Suicide Severity Rating Scale
DSMB	Data and Safety Monitoring Board
DSM-5	Diagnostic and Statistical Manual of Mental Disorders
EEG	Electroencephalography
EMG	Electromyography
FDA	Food and Drug Administration
GABA	Gamma-Aminobutyric Acid
GCP	Good Clinical Practice
GEE	Generalized Estimating Equation
HIPAA	Health Insurance Portability and Accountability Act
IDE	Investigational Device Exemption
IFU	Instructions For Use
ISI	Interstimulus Interval
IRB	Institutional Review Board
iTBS	Intermittent Theta Burst Stimulation
LDPFC	Left Dorsolateral Prefrontal Cortex
LICI	Long Interval Intracortical Inhibition
MDD	Major Depressive Disorder
MEP	Motor Evoked Potential
MINI	Mini International Neuropsychiatric Interview
MINI-KID	Mini International Neuropsychiatric Interview for Children and Adolescents
MT	Motor Threshold
NIH	National Institutes of Health
NIMH	National Institute of Mental Health
OCER	Office of Community Engagement
OSHA	Occupational Safety and Health Administration Guidelines
PHI	Protected Health Information

PI	Principal Investigator
RDPFC	Right Dorsolateral Prefrontal Cortex
SAE	Serious Adverse Event/Serious Adverse Experience
SI	Suicidal Ideation
SICI	Short-Interval Intracortical Inhibition
SOP	Standard Operating Procedure
TBS	Theta Burst Stimulation
TMS	Transcranial Magnetic Stimulation
TMS-EEG	Transcranial Magnetic Stimulation-Electroencephalography
UADE	Unanticipated Adverse Device Effect
UPIRTSO	Unanticipated Problems Involving Risks to Subjects or Others
YMRS	Young Mania Rating Scale

The trial will be conducted in accordance with the ICH E6, the Code of Federal Regulations on the Protection of Human Subjects (45 CFR Part 46), Investigational Device Exemption (IDE) regulations, all applicable FDA regulations stipulated in the Investigator Agreement (21 CFR Part 812, 21 CFR Part 50, 21 CFR Part 54, and the NIH IC Terms of Award. The Principal Investigator will assure that no deviation from, or changes to the protocol will take place without prior agreement from the sponsor and documented approval from the Institutional Review Board (IRB), except where necessary to eliminate an immediate hazard(s) to the trial participants. All personnel involved in the conduct of this study have completed Human Subjects Protection Training and Good Clinical Practice Training.

I agree to ensure that all staff members involved in the conduct of this study are informed about their obligations in meeting the above commitments.

Principal Investigator: ___________________________________

Signed: ______________________________________

Date: ______________

Study Summary

Title	*A Randomized Controlled Trial of Sequential Bilateral Accelerated Theta Burst Stimulation in Adolescents with Suicidal Ideation Associated with Major Depressive Disorder*
Running Title	*Sequential Bilateral aTBS for Adolescent SI*
IRB Protocol Number	20-009630
Phase	*Pilot*
Methodology	*Randomized, double-blind, sham controlled*
Overall Study Duration	*5 years*
Subject Participation Duration	*13 months*
Study Interventions	*The proposed study will examine sequential bilateral accelerated theta burst stimulation (aTBS). Three sessions are administered daily for 10 days (5 days per week). During each session continuous theta burst stimulation (cTBS) in which 1800 pulses are delivered continuously over 120 seconds to the right dorsolateral prefrontal cortex (RDPFC) is administered first, followed by iTBS in which 1800 pulses are delivered in 2 second bursts, repeated every 10 seconds for 570 seconds (1800 pulses) to the left dorsolateral prefrontal cortex (LDPFC). The theta burst stimulation (TBS) parameters were adopted from prior work, with 3-pulse 50 Hz bursts given every 200 ms (at 5 Hz) with an intensity of 80% of active motor threshold. The comparison group will receive 3 daily sessions of bilateral sham TBS treatment for 10 days. Subjects in both groups will take part in a daily psychotherapeutic treatment program.*

Aims	Aim 1 examines the efficacy as assessed by the Columbia Suicide Severity Rating Scale (C-SSRS) severity of ideation subscale of sequential bilateral aTBS compared to sham stimulation over 10 days of active treatment and 12 months of follow-up. Aim 1 will also examine the impact of active vs sham sequential bilateral aTBS treatment on the number of emergency department visits and hospitalizations related to suicidality.<br>Aim 2 examines a biomarker of cortical inhibition called long interval cortical inhibition (LICI). It is expected that LICI will have an indirect relationship with severity of suicidal ideation as assessed with the C-SSRS severity of ideation subscale in all subjects at baseline. It is also expected that in adolescent subjects receiving active sequential bilateral aTBS treatment there will be an indirect relationship between LICI and the C-SSRS severity of ideation subscale over 10 days of treatment.<br>Exploratory aims focus on the development of TMS-EEG measures of cortical inhibition.
Number of Subjects	*80*
Study Population	*The study will enroll outpatient and inpatient adolescents (aged 12-18 years) with Major Depressive Disorder (MDD) of at least moderate severity defined as a Children's Depression Rating Scale Revised (CDRS-R) Score of 40 or greater and suicidal ideation defined as a score of 3 or greater on item 13 (Suicidal Ideation) of the CDRS-R* and a minimum score of 1 ("wish to be dead") on the C-SSRS severity of ideation subscale
Investigational Study Device	*The device consists of a magnetic stimulator, MagPro X100 with MagOption and a magnetic coil, based on the Cool-B70 (Please refer to K173620). The coil is specifically designed for double-blind, sham-controlled trials, as it contains an active site (A, identical to Cool-B70) and a sham side (P) in which the magnetic field is reduced < 5% of the active site. This coil is called Cool-B70 A/P.*

Duration of Exposure	*Subjects in the active arm will receive 11.5 minutes sessions of sequential bilateral TBS at 80% motor threshold, 3 times daily (1 hour apart) for 10 days in total (5 days per week). Each session includes cTBS in which 1800 pulses are delivered continuously over 120 seconds to the right dorsolateral prefrontal cortex (RDPFC) followed by iTBS in which 1800 pulses are delivered in 2 second bursts, repeated every10 seconds for 570 seconds (1800 pulses) to the left dorsolateral prefrontal cortex (LDPFC). The TBS parameters were adopted from prior work, with 3-pulse 50 Hz bursts given every 200 ms (at 5 Hz) with an intensity of 80% of active motor threshold. All subjects will be followed for 12 months after the 10 day treatment course to assess safety and ongoing clinical effects.*
Reference therapy	*A comparison group will receive 3 daily bilateral sessions of sham TBS. Sessions in the comparison group are 11.5 minutes in duration, three times daily, for 10 days total (5 days per week).*

Statistical Methodology	*Suicidal Ideation as measured by the Columbia-Suicide Severity Rating Scale (C-SSRS) severity of ideation subscale over the 10-day sequential bilateral aTBS trial period will be the primary ordinal outcome measure. The acute change over time (10 days) in Suicidal Ideation will be compared between the two treatment groups (sequential bilateral aTBS vs. sham stimulation) using an ordinal logistic regression model within a Generalized Estimating Equation (GEE) framework. The logistic model will contain fixed effects terms for treatment, time, treatment × time interaction along with baseline C-SSRS severity of ideation subscale as a covariate. Age, sex, pubertal status, antidepressant medication status, and depression severity (CDRS-R total as a time-varying covariate) will also be included as covariates in the model. Simple treatment group effects in each time period will also be assessed. In particular, we will examine for "rapid-acting" treatment effects on day 2 and day 3 of the 10-day aTBS protocol. Maximum likelihood estimation and Type 3 tests of fixed effects will be used along with the best fitting covariance structure. The sandwich (robust covariance matrix) estimator will also be considered. For the ordinal logistic regression, the cumulative probabilities will be modeled over the lower-ordered suicidal ideation scale scores (less suicidal ideation). The adjusted odds ratio (i.e., odds of suicidal ideation from treatment status), and 95% confidence interval, will be estimated as part of the GEE model so as to interpret the omnibus treatment effect. The odds ratio will also be interpreted as the effect size estimator for the between-subjects treatment effect. The PROC GENMOD procedures (along with maximum likelihood estimation) in SAS software will make use of all available data from all participants from the efficacy analysis set, and provide a robust mechanism for handling data that are assumed missing at random.*

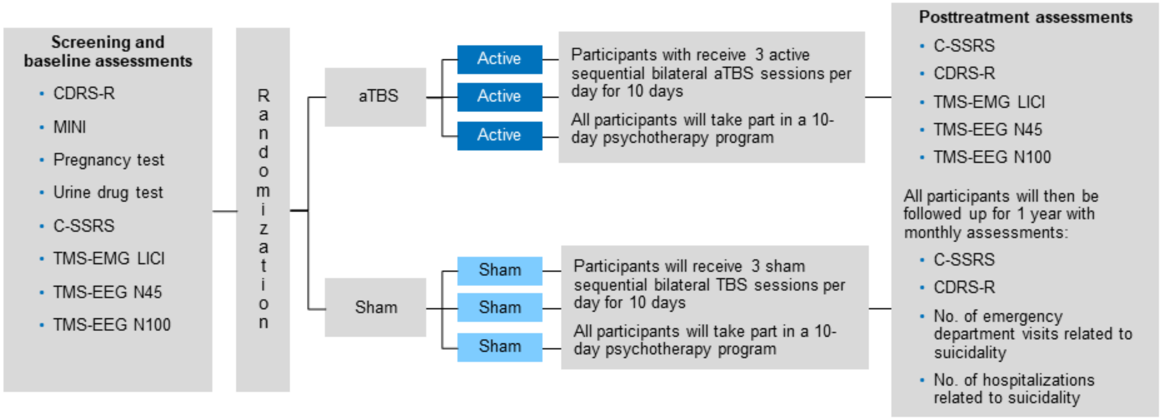

Study Overview

aTBS, Accelerated Theta Burst Stimulation

CDRS-R, Children's Depression Rating Scale Revised

C-SSRS, Columbia-Suicide Severity Rating Scale

MINI, The Mini-International Neuropsychiatric Interview (for age 18) or the Mini-International Neuropsychiatric Interview for Children and Adolescents (for ages 12-17)

TBS, Theta Burst Stimulation

TMS-EEG, Transcranial Magnetic Stimulation with Electroencephalography

TMS-EMG, Transcranial Magnetic Stimulation with Electromyography

1 Introduction

This document is a protocol for a human research study. This study will be carried out in accordance with the procedures described in this protocol, applicable United States government regulations and Mayo Clinic policies and procedures.

1.1 Background

Suicide is one of the leading causes of death in adolescents worldwide and a substantial public health problem.[1-3] In the United States, 18% of teenagers report seriously considering suicide, 15% have made a suicide plan and 9% have made an attempt within the preceding year.[4, 5] Suicidal ideation and attempts at early life predict similar behaviors in adulthood.[6] In the context of these grim statistics, there are limited effective treatments for suicidal ideation in adolescents with major depressive disorder (MDD).[6-8] Outcomes related to suicidality in adolescents have failed to improve over many years.[4]

Prior research focused on suicide in adolescents involves understanding risk factors, examining trends, and considering large initiatives to reduce overall rates.[4, 8-10] Unfortunately, the predictive value of identified risk factors is limited and has not improved much over time despite decades of research.[8] Many biological correlates investigated to date are also weak predictors of suicidal behaviors.[8,11-13]Less attention has been paid to developing new, rapid-acting treatments for adolescents with MDD who are actively struggling with thoughts of suicide or have recently attempted suicide.[7, 14] Recent research has examined the anti-suicidal effects of alternative treatments in adult populations but there are substantial knowledge gaps with respect to adolescents.[8, 15, 16] There are essentially no standard brain-based intervention targeting suicidal ideation in adolescents with MDD. The standard of care for suicidal ideation in adolescents is also arguably poorly defined.[14]

Prior research demonstrates that suicidal patients have structural, functional, neurophysiological, and neuroplasticity alterations in the dorsolateral prefrontal cortex (DPFC).[17-19] For example, structural alterations and serotonergic dysfunction in the DLPFC are associated with lethality of suicide attempts.[19-21]Suicidal patients also demonstrate abnormalities in processing negative emotional stimuli and DLPFC activation. These latter findings vary across studies suggesting multiple neural phenotypes of suicidality that are not well understood.[19] Previous work suggests that suicidal adolescents with depression have pathophysiological DLPFC deficits in gamma-aminobutyric acid (GABA) function. [22-25]Conceptually, impaired prefrontal, GABAergic inhibitory gating related to depression, may yield reduced control over an adolescent's thoughts and behaviors culminating in suicidal ideation and behaviors.[17, 26, 27] Interventions with transcranial magnetic stimulation (TMS) directly address these underlying pathophysiological deficits in the DLPFC.[28, 29]

Recent work with TMS interventions to address suicidal ideation in adults with MDD demonstrates that bilateral TMS has a strong clinical effect.[30, 31] Preliminary work with TMS to address suicidal ideation in adolescents is promising.[14] Treatment with TMS most likely addresses pathological imbalances in prefrontal GABAergic inhibitory function underlying depression and suicidal ideation in adolescents.[14, 22, 23, 29, 32] However, standard 10 Hz TMS does not typically produce rapid relief from suicidal ideation and depressive symptoms.[31, 33, 34] TBS is a type of TMS that induces changes in synaptic plasticity more rapidly than standard

TMS.[35-38] Protocols with TBS have demonstrated **safety in adolescents, are more tolerable than standard TMS, and are delivered over brief sessions** (approximately 12 minute sessions compared to 37 minute sessions with standard TMS).[39, 40] TBS was developed by mirroring endogenous hippocampal discharges.[37] In TBS dosing 3 pulse 50 Hz bursts are delivered at 5 Hz which is every 200 milliseconds (ms). Stimulation with iTBS has been shown to produce long-term potentiation like effects[37, 41, 42] while cTBS reduces cortical excitability.[37, 43] Prior studies of sequential bilateral TBS deliver cTBS to the right prefrontal cortex followed by iTBS delivered to the left prefrontal cortex during each session.[39, 44] Conceptually, this targets hypoactivity of the left DLPFC and hyperactivity of the right DLPFC associated with MDD and suicidal ideation.[19, 45, 46] Sequential, bilateral TBS approaches have demonstrated greater clinical effects for depressive symptoms and suicidal ideation compared to unilateral TBS.[30, 31, 44] Accelerated TBS (aTBS) dosing delivers multiple daily sessions of TBS.[47] Recent work suggests that aTBS protocols more rapidly and effectively improve depressive symptoms and improve synaptic plasticity in the prefrontal cortex. [36, 48, 49] These aTBS protocols also overcome many practical limitations of standard TMS.[47]

Prior clinical translational work demonstrates that prefrontal cortex GABAergic tone is dysregulated in adolescent patients with suicidal ideation and MDD.[22, 23, 25, 50] The relationship between abnormal GABA neurotransmission and suicidal ideation is more prominent in the context of depression or Negative Valence System perturbations.[25, 51] Studies have demonstrated abnormal messenger ribonucleic acid expression of the $GABA_A$ subunit in depressed patients completing suicide, aberrant deoxyribonucleic acid methylation of the $GABA_A$ alpha-1 subunit promotor region in depressed suicide completers, and presynaptic dysfunction related to $GABA_B$ functionality in depressed suicide victims.[52-55] Notably, GABAergic function undergoes considerable change during neurodevelopment.[56-58] Prior studies support the relationship between GABAergic neurotransmission and adolescent suicidal ideation and behaviors.[24, 25, 50]

Neurophysiological studies employing TMS integrated with electromyography (EMG) or electroencephalography (EEG) have demonstrated promise in assessing cortical inhibition biomarkers.[59-61] Cortical inhibition paradigms such short-interval intracortical inhibition (SICI) and long-interval intracortical inhibition (LICI) index cortical $GABA_A$ and $GABA_B$ mediated inhibitory neurotransmission respectively.[61-63] While data collected with TMS-EEG paradigms may yield more direct measures of prefrontal cortex function there are a number of contemporary methodologic controversies.[64-68] Further, TMS-EEG measures have not been reliably collected in adolescents with depression and suicidal ideation.[67] Conversely, TMS-EMG measures have been extensively studied in adolescents with depression and could easily be implemented within clinical environments.[24, 59, 69-71]Adolescents with MDD and suicidal behaviors have deficient LICI with related $GABA_B$ impairment.[25] Other recent work demonstrates that LICI markers improve with concurrent decrements in suicidal ideation in adolescents treated for depression.[50] Hence, TMS-EMG, LICI is a promising biomarker for the assessment and treatment of adolescents with depression and suicidal ideation.

The proposed study will examine sequential, bilateral, aTBS (3 sessions daily for 10 days, 5 days per week). Each session has continuous theta burst stimulation (cTBS) in which 1800 pulses are delivered over 120 seconds to the RDPFC and iTBS in which 1800 pulses are delivered in 2 second bursts, repeated every 10 seconds for 570 seconds (1800 pulses) to the LDPFC. During each session the cTBS is first delivered to the RDPFC and then iTBS is

delivered to the LDPFC. The TBS parameters were adopted from prior work, with 3-pulse 50 Hz bursts given every 200 ms (at 5 Hz) with an intensity of 80% of active motor threshold (MT).[29, 35, 39] The comparison group will receive 3 daily session of bilateral (delivered to alternately to the RDPFC and LDPFC) sham TBS treatment for 10 days. Accelerated protocols administer multiple sessions of TMS or TBS daily. The rationale for accelerated dosing is based on the idea that multiple stimulations in a shorter time frame will yield more rapid, greater, and durable efficacy in a shorter time frame as compared to standard, daily TMS sessions. Recent work with aTBS dosing for adult patients with highly refractory depression has produced encouraging results.[36, 49]Few prior studies have investigated the use of theta burst and accelerated protocols in adolescents. Available findings, however, are encouraging and suggest that the safety profile in adolescents is similar to adults.[39] Prior work also suggests that repeated TBS sessions safely enhance neurophysiological and clinical changes.[39, 40, 72]

The potential impact: The proposed research study addresses the National Institute of Mental Health Strategic Research Strategies 3.2 Develop ways to tailor existing and new interventions to optimize outcomes and 3.3 Test interventions for effectiveness in community practice settings.[73, 74] This study will follow an experimental medicine approach[75] implemented in an intensive outpatient program setting that supports local emergency departments, an 18 bed adolescent inpatient psychiatry unit, and outpatient clinics. The study will examine the efficacy of sequential bilateral, aTBS for suicidal ideation in adolescents with MDD. The study will use an LICI TMS-EMG biomarker to monitor treatment with bilateral aTBS and index the severity of suicidal ideation. Concurrent work with TMS-EEG will provide opportunities to further understanding of the neurophysiological and molecular mechanisms of suicidal ideation in adolescents while refining methodology for future clinical use.[68, 69]

1.2 Investigational Device

The MagVenture TMS Therapy System w/Theta Burst Stimulation will be used under the subject investigational device exemption (IDE). The device consists of a magnetic stimulator, MagPro X100 with MagOption (K173620), and a magnetic coil used for MT determination, C-B70 and a treatment coil, the Cool-B70 A/P coil, both of which have not obtained prior FDA clearance (Please refer to K173620).[35] The Cool-B70 A/P coil is a coil designed specifically for use in double-blind, sham-controlled trials. This coil is identical to the Cool-B70 coil (K173620) and the C-B70 in terms of magnetic field properties. The A/P version of the coil, contains both an active site (A) and a sham (P) site. The magnetic field on the sham site is significantly reduced, and is less than 5% of that of the active site. The magnetic field properties on the active site are identical to that of the standard Cool-B70 coil, cleared for iTBS treatment (K173620). The MT coil, C-B70, is identical in terms of magnetic field properties to the Cool-B70 and the Cool-B70 A/P coils, but is a lightweight, non-liquid cooled coil that allows for an easier MT determination and is necessary in order to keep the blind of both operator and patient.

The product code is OBP, and the classification regulations number for the System is 21 CFR 882.5805. All The majority of components in the subject pre-sub have previously obtained FDA clearance (K173620), except the Cool-B70 A/P and the C-B70 coils.

1.3 Preclinical Data

Our lab has an ongoing collaboration with the Rodger Lab in the Department of Experimental and Regenerative Neurosciences at the University of Western Australia focused on murine models of TMS delivery.[76] These efforts yield high throughput studies to examine novel dosing for human TMS protocols. Recent work examined a chronic restraint stress depression model in rats undergoing a standard low-intensity TMS protocol, an accelerated low-intensity TMS protocol, or sham TMS. Rats receiving low-intensity accelerated TMS had greater improvements in behavioral test outcomes for depression (Figure 1). Magnetic resonance spectroscopy scans revealed no significant changes in cortical N-acetylaspartate (NAA) from pre- to post-treatment. Given that NAA is a measure of neuronal health and integrity these data provide preclinical support for the safety of accelerated TMS protocols (Figure 2).[48]

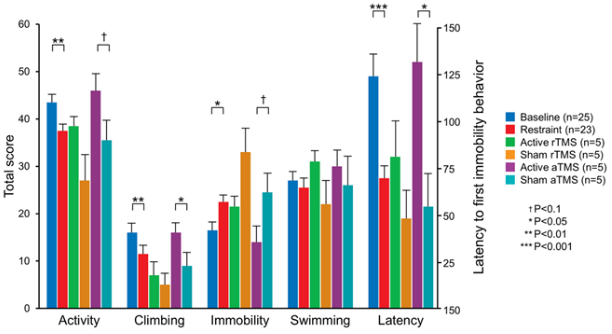

Figure 1. Preclinical study with accelerated TMS

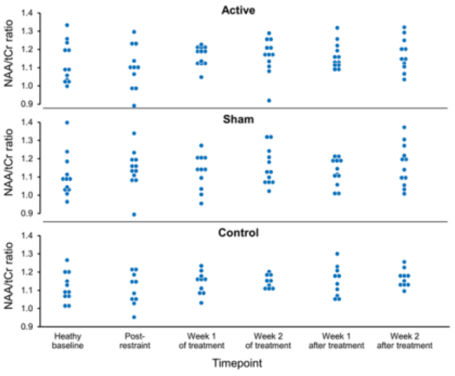

Figure 2. Preclinical safety data with accelerated TMS

Electric-field (E-field) modeling Prior research and our own work examining neuroimaging guided and Beam F3[79] localized treatments supports the use of the Beam F3 approach as a valid and reliable coil localization method.[77, 78] Electric field modeling has demonstrated comparable stimulation intensities for adolescents as compared to adults with Beam F3 coil localization.[77]

Nevertheless, we examined model calculations specific to the equipment used in the proposed study (Figure 3). Model calculations were performed of the field from the Cool-B70 transducer which is a butterfly type of coil with two partially overlapping bend coils. An adult human head was modelled by a homogeneous sphere with a radius of 85 mm and an isotropy conductivity of 0.33 S/m. For an adolescent 12 years of age the head circumference is typically 4-5% lower than for an adult.[79, 80] A model calculation was therefore made where the model head radius is reduced from 85 to 81mm (i.e. a 4.7% reduction). The figure shows the coil over respectively an adult 85mm radius and an 81mm child head model. In both cases the coil is placed corresponding to the usual placement where the outer plastic keeper surface is in contact with the patient head (Figure 3A). The induced E-field on the surface of the adult head had a maximum field of 497 volt per meter (V/m) and the surface field on the adolescent head had a

slightly reduced maximum field of 488 V/m. As the transducer coil is place in the same scalp configuration the surface field maximum change is relative small (1.8% reduction for the adolescent head). The smaller head results in a lower magnetic flux in the adolescent head volume and therefor a slightly reduced electrical field level (Figure 3B). In adults, the MT is determined for the motor cortex at a typical nominal depth of 20mm. The E-field distribution on a radius 65mm sphere at a depth of 20mm which gives a maximum E-field of 164 V/m. For a child the corresponding MT depth is assumed to scale with the head radius and be reduced from 20mm to 81/85*20 = 19.1 mm. The E-field distribution for the smaller adolescent model at a depth of 19.1mm which gives a maximum E-field of 168 V/m corresponding to a modest 2.4% increase (due to the reduced distance from the coil to the motor cortex). We can thus conclude that the induced field difference among adolescent and adults is within a few percentage points. This is a relative small difference as compared to the variation that is observed between different adults. We then modelled the proposed dosing with the Beam F3 localization technique (Figure 3C).

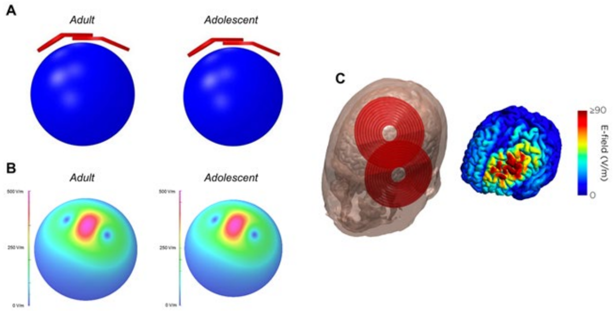

Figure 3. Electric-field (E-field) modeling

1.4 Clinical Data to Date

Pilot Data and Preliminary Work Support Innovative Methodology and Feasibility

Our lab has pioneered recent innovations and adaptions of TMS for adolescent populations.[26] This study capitalizes on several areas of innovation while building our existing experience, expertise, and infrastructure. We will conduct the first double-blind, randomized controlled trial of sequential bilateral aTBS for suicidal ideation in adolescents with MDD in the context of a clinical program. This study will recruit participants who are excluded from our prior and existing protocols. The research methodology will target and track suicidal ideation, which in adolescents is a pernicious public health problem with underdeveloped interventions. This study follows an experimental medicine approach by evaluating a standard marker of cortical inhibition for baseline correlation with suicidal ideation severity and subsequent target engagement.[73, 75]The research team will also collect TMS-EEG measures of cortical inhibition to advance understanding of the neurophysiology of adolescent suicidality, develop additional reliable tools to probe cortical inhibition, and study GABAergic neuro-circuitry in adolescents.[25, 81]

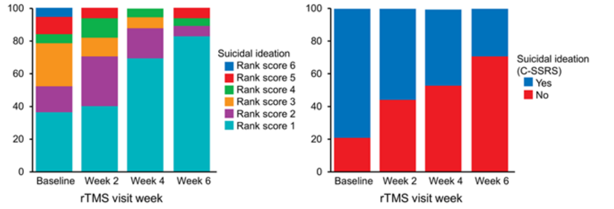

Figure 4. TMS improves suicidal ideation in adolescents with MDD

TBS treatment for Suicidal Ideation in Adolescents: A prior study providing 30 sessions of open-label 10 Hz TMS for adolescents (N=19) with MDD examined suicidal ideation at baseline, after 10 treatments, after 20 treatments, and after 30 treatments.[14] Outcome measures

of suicidal ideation were assessed with the Columbia Suicide Severity Rating Scale (C-SSRS) intensity of ideation subscale and item 13 (suicidality) on the Children's Depression Rating Scale Revised (CDRS-R). The predicted odds of suicidal ideation significantly decreased over 6 weeks of acute TMS treatment with both outcome measures (Figure 4).[14] A more recent study provided 10 daily sessions of sequential bilateral TBS to depressed adolescents (N=20).[39] Sequential bilateral TBS (at the dosing proposed in this application) was well tolerated in this sample of adolescents and demonstrated antidepressant effects. At baseline 17 of 20 of the subjects reported suicidal ideation with a mean C-SSRS intensity of ideation score of 3.11 and standard deviation (SD) of 1.45. After 10 daily sessions of sequential bilateral TBS, 9 subjects reported suicidal ideation with a mean C-SSRS suicidal ideation score of 2.11 (SD = 1.27).[39]

In preparation for study design our group reviewed current literature examining accelerated TMS protocols and published a meta-analysis (Figure 5). There was variability in published protocols. Summary analysis of three randomized controlled trials yielded an effect size (Hedges' g) of 0.4. Another analysis of all active arms and open-label studies revealed an effect size (Hedges' g) of 1.3.[47] Preliminary results suggested that aTBS improved suicidal ideation in adults with MDD.[49]

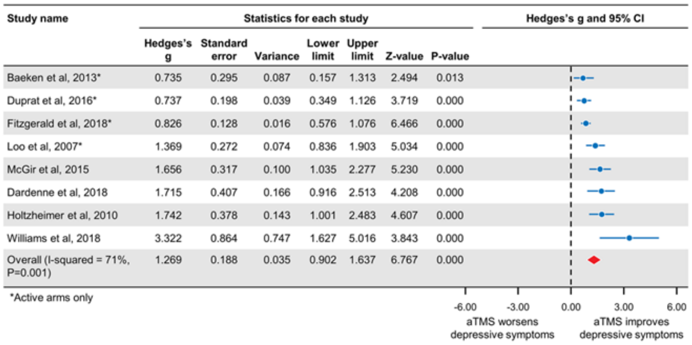

Study name	Hedges's g	Standard error	Variance	Lower limit	Upper limit	Z-value	P-value
Baeken et al, 2013*	0.735	0.295	0.087	0.157	1.313	2.494	0.013
Duprat et al, 2016*	0.737	0.198	0.039	0.349	1.126	3.719	0.000
Fitzgerald et al, 2018*	0.826	0.128	0.016	0.576	1.076	6.466	0.000
Loo et al, 2007*	1.369	0.272	0.074	0.836	1.903	5.034	0.000
McGir et al, 2015	1.656	0.317	0.100	1.035	2.277	5.230	0.000
Dardenne et al, 2018	1.715	0.407	0.166	0.916	2.513	4.208	0.000
Holtzheimer et al, 2010	1.742	0.378	0.143	1.001	2.483	4.607	0.000
Williams et al, 2018	3.322	0.864	0.747	1.627	5.016	3.843	0.000
Overall (I-squared = 71%, P=0.001)	1.269	0.188	0.035	0.902	1.637	6.767	0.000

Figure 5. Prior studies of accelerated TMS

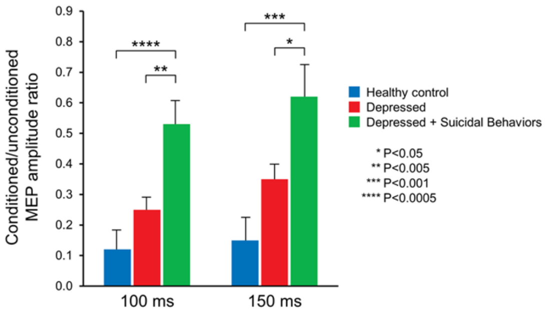

Figure 6. Indexing suicidal ideation with long-interval cortical Inhibition (LICI) biomarkers

The paired-pulse paradigm LICI provides an index of $GABA_B$ receptor mediated inhibition.[70, 82, 83] A suprathreshold test stimulus is applied to the motor cortex eliciting a motor evoked potential (MEP) in the contralateral abductor pollicis brevis electrode. During LICI testing, suprathreshold conditioning and test stimuli are applied with interstimulus intervals (ISIs) of 100-200 ms which results in a MEP of diminished amplitude. This resultant inhibition is mediated by $GABA_B$ receptors.[61] Our prior studies demonstrate that depressed adolescents with suicidal behaviour have impaired LICI and $GABA_B$ mediated inhibition[7, 12] compared to depressed adolescents with no history of suicidal behaviors and healthy controls. Please note that larger LICI values denote diminished inhibition (Figure 6). In another longitudinal study of suicidal adolescents in treatment for depression, enhancements in LICI measures correlated with improvements in suicidal ideation (assessed with the C-SSRS subscale for severity of suicidal ideation) after controlling for depression severity.[50] Other recent work demonstrated the utility of TMS-EEG measures of LICI and N100 as target engagement biomarkers for brain stimulation interventions of suicidal ideation in adults with

depression.[81] These TMS-EEG measures may be more precise markers of cortical inhibition but they have not been validated in adolescents.[67, 68]At present there are also methodological and interpretation controversies regarding TMS-EEG for studies of psychiatric disease.[64-66, 84] Collectively these studies demonstrate that LICI could be readily implemented as a clinical biomarker to assess suicidality and guide treatment with TBS interventions in adolescents with depression.[25, 50, 70]

1.5 Study Rationale and Risk Analysis (Risks to Benefits Ratio)

1.5.1 Study Rationale

The proposed study will examine sequential bilateral aTBS. There are limited data regarding the use of theta burst and accelerated protocols in adolescents.[30, 39] Available data are encouraging and suggest that the safety profile in adolescents is similar to adults.[39, 40, 72, 85-87] Prior work also suggests that repeated TBS sessions safely enhance neurophysiological and clinical changes.[36, 88] A recent, large American survey of seizure risks in the period of 2012-2016 also shows that the risk of seizure is comparable between iTBS and standard TMS, and that the risk is very low.[85, 89]

Twelve prior relevant studies including subjects aged 18 and younger are summarized in Table 1. As noted, studies have utilized cTBS, iTBS, and bilateral stimulation in this age group. One study delivered multiple daily sessions.[87] In general, the reported adverse events were mild and similar to what is noted in adult studies.[85-87] One case report described a seizure induced by iTBS.[90]

In 2012, Wu and colleagues reported on a single session study of TBS in forty subjects (N=16 with Tourette syndrome and N=24 healthy controls) under 18 years of age.[91] The TBS was delivered in a manner consistent with standard protocols. The 50 Hz bursts of stimulation were delivered at 5 Hz. The iTBS protocol delivered three, 50 Hz pulses every 200 ms for 2 seconds and this was repeated every 10 seconds for 20 trains total. The cTBS sessions delivered three 50 Hz pulses every 200 ms repeated 200 times. In this study 31 subjects received 32 sessions of TBS at 80% active MT. Young children (<12 years of age) often had higher MTs with stimulation intensities that exceeds device capabilities and are not possible to deliver. In these cases, the study team delivered two sessions of TBS at 55% and 70% active MT. The team also changed the TBS burst delivery to 30 Hz to extend stimulation intensity capability. Subsequently, 7 subjects received 9 sessions of 30 Hz TBS at an intensity of 90% resting MT. Three of the forty subjects also received cTBS. One subject received iTBS and cTBS on the same day (sessions 2 hours apart). There were no serious adverse events related to TBS. Three subjects reported a mild headache, one subject noted mild neck stiffness, and one subject noted a mild subjective feeling of a finger twitch which could not be visualized and resolved within 12 hours with no intervention. The authors concluded these data suggest that a single session of TBS is tolerable and safe in children aged 18 years and younger.[91]

A later follow-up study in 2012 by Wu and colleagues examined the effects of a single iTBS session on motor-evoked potential amplitudes in a sample of subjects aged 18-42 years (N=10 with Tourette Syndrome and N=11 healthy controls).[92] The subjects received one session of iTBS delivered to the left motor cortex. The iTBS protocol delivered three, 50 Hz pulses every

200 ms for 2 seconds and this was repeated every 10 seconds for 20 trains total at 80% active MT. There were no reported serious adverse events or adverse events in this study.[92]

In 2014, Wu and colleagues reported on a sham-controlled trial for 30 Hz cTBS in subjects (N=13) with chronic tic disorders.[87] One 10-year-old subject had a high MT and as a result did not receive cTBS. Otherwise, six subjects received active cTBS and six received sham cTBS both applied to the left motor cortex. In the group receiving active cTBS there were two 10-year-old subjects, a 2-year-old subject, a 13-year-old subject, and a 16-year-old subject. Active cTBS consisted of 5Hz/30 Hz delivered at 90% resting MT. Four trains of cTBS were administered daily for 2 days. The first and second trains of cTBS were spaced 15 minutes apart. The third train of cTBS was 60 minutes after the first train and the fourth train of cTBS was 75 minutes after the first train of cTBS. All (N=12) subjects who received active or sham cTBS completed the study. Three patients receiving active cTBS reported mild adverse events (abdominal pain, headache, and dry eyes). The authors highlighted that child subjects tolerated 8 trains of cTBS over 2 days with no serious adverse events.[87]

Oberman and colleagues reported on a study of autism spectrum disorder in 2014.[93] Male subjects, aged 9-18 years (N=19) with autism spectrum disorder underwent a 40 second train of 5Hz/50Hz cTBS at 80% active MT, delivered to the left motor cortex. The cTBS protocol was tolerated with no serious adverse events. Adverse events included a mild headache that was relieved with one dose of acetaminophen. Two other subjects noted mild fatigue that resolved the next day. The authors noted that these data provided initial support for the safety and tolerability of TBS in pediatric populations.[93]

A 2015 study by Pedapati and colleagues examined an iTBS protocol in younger subjects.[94] In this study, healthy children, aged 10-16 years (N=14) received 300 pulses of 5Hz/30Hz iTBS at 70% resting MT. The authors related that no adverse events were reported spontaneously or with structured interviews. There were no seizures. The authors highlighted that their findings provide additional reassurance that iTBS can be delivered safely in children.[94]

Another 2015 study by Hong and colleagues sought to compare the safety and tolerability of TBS protocols with single and paired-pulse TMS protocols.[40] The authors retrospectively examined data from (N=165) subjects 6-18 years of age, from various protocols in 2009 through 2014. The studies collected adverse event data with a structured questionnaire. The TBS protocols delivered intensities ranging from 60-90% of resting MT and 30-50 Hz pulse frequencies delivered in 5 Hz bursts. The total number of pulses in TBS protocols was either 300 or 600. Nine of the subjects received both cTBS and iTBS stimulation. Seventy-six subjects were exposed to TBS protocols and eight-nine underwent single or paired-pulse TMS. There was no statistical difference among these two groups with respect to adverse events. Common specific adverse events with TBS included headache (6.6%), arm/hand/other pain (2.6%), numbness or tingling (2.6%), other sensations (2.6%), weakness (1.3%) and other (1.3%). While this study is not without limitations, the results were encouraging. Some consensus guidelines suggest that single and paired-pulse TMS confers minimal risk in studies. Notably, the safety profile in children undergoing TBS was similar to single and paired-pulse TMS. The authors noted that this was the largest study to date examining the safety and tolerability of TBS protocols in children.[40]

In 2016 Pedapati and colleagues examined the neurophysiological effects and tolerability of iTBS in youth with autism spectrum disorder 13-18 years of age (N=9) and healthy control

subjects 11-18 years of age (N=9).[95] Subjects received 300 pulses of 5Hz/30 Hz iTBS at 70% resting MT delivered to the left motor cortex. Adverse event monitoring included a structured interview. One subject noted a mild headache after the iTBS session that resolved with no intervention. Otherwise, iTBS was tolerable to all subjects. There were no seizures or serious adverse events.[95]

Oberman and colleagues also examined a cTBS protocol in 2016 to study plasticity and metaplasticity in patients with autism spectrum disorder and fragile X syndrome.[95] Subjects with autism spectrum disorder (N = 10, 16-62 years of age), fragile X syndrome (N=6, 16-33 years of age), and healthy controls (N=12, 19-61 years of age) underwent 2 sessions of cTBS on 2 consecutive days. During the protocol, 600 pulses of 5Hz/50Hz cTBS at 80 % active MT applied to the left motor cortex. The cTBS was well tolerated and no adverse or serious adverse events were reported.[95]

In 2016, Dileone and colleagues reported on a study that examined the neurophysiological effects of iTBS on patients with Costello syndrome and healthy controls.[96] The experimental protocol delivered 600 pulses of 5Hz/50 Hz iTBS at 80% active MT to the right motor cortex. The samples included patients (N=4) aged 17, 18, 19, and 27 with Costello Syndrome and 21 control patients ranging 16-34 years old. There were no adverse or serious adverse events in the study.[96]

Dr. Abujadi and colleagues presented findings from an interventional study in a 2018 publication.[72] This effort enrolled male subjects aged 9-17 (N=10) with autism spectrum disorder for an open-label trial of iTBS. Subjects received fifteen sessions with 900 pulses per session of 5 Hz/50 Hz iTBS at 100% active MT delivered to the RDPFC. The iTBS was tolerated by all subjects. All subjects completed the study. There were no adverse events, seizures or serious adverse events. The authors highlighted some promising, albeit preliminary findings on clinical effects.[72]

In 2018, Dr. Purushotham and colleagues published a case report describing a seizure in a teenager during iTBS.[90] A 15 year-old, female patient with Schizophrenia was enrolled in a neurophysiological study arm meant to deliver 600 pulses of iTBS at 80% active MT. The subject had no prior or current treatment with psychotropic medications. Prior to iTBS a physical examination, electrocardiogram, electrolytes, complete blood count, liver function tests, renal function tests, thyroid function tests, and computed tomography of the brain were all normal. There was no personal history of head injury, seizures, or other neurological disorders. The family psychiatric and neurological history was normal. The authors described a seizure with limb muscle contractions, eye lid fluttering, jaw contraction, foamy saliva, and urinary incontinence. The subject regained consciousness after 1 minute but was disoriented for approximately 20 minutes. The authors highlighted that this was the first published seizure with TBS in a child or adolescent.[90]

Dr. Dhami and colleagues recently completed a therapeutic trial of TBS with one of the most aggressive protocols to date.[39] This study enrolled twenty youth (ages 16-24) with major depressive disorder. The subjects received ten sessions of sequential, bilateral 5 Hz/50 Hz TBS. Each session included 1800 pulses of cTBS delivered to the RDPFC followed by 1800 pulses of iTBS delivered to the LDPFC prefrontal cortex alternately. All TBS sessions were delivered at 80% active MT. The order of iTBS and cTBS was randomized for each patient. Subjects

endorsed mild headaches and other mild adverse events. There were no seizures or serious adverse events. The authors highlighted promising, preliminary clinical effects.[39]

Collectively this prior work suggests that TBS interventions have similar safety, tolerability, feasibility, and clinical effects in adolescents as compared to adults. Treatment with TBS may improve suicidal ideation in adolescents. Novel, aTBS protocols offer similar safety profiles and clinical effects in adults with depression as compared to standard TBS protocols.[36, 47, 87] With further study, aTBS protocols could provide pragmatic advantages in the treatment of adolescents with suicidal ideation.[14, 47, 69]

Table 1. Prior TBS Studies in Children and Adolescents

Study	Age and (N)	Stimulation Intensity	Number of sessions per day	Total number of sessions	Adverse Events
Proposed Study	12-18 years (N=80)	80% Active MT	3	30	
Wu et al. 2012[91]	8-17 years (N=40)	80% Active MT	1	1	Mild headache 7.0%<br>Neck stiffness 2.3%<br>Sensation of finger twitching 2.3%
Wu et al. 2012[92]	18-42 years (N=21)	80% Active MT	1	1	None reported
Wu et al. 2014[87]	10-22 years (N=13)	90% Resting MT	4	8	Mild abdominal pain 16.7%<br>Mild headache16.7%<br>Mild dry eyes 16.7%
Oberman et al. 2014[93]	9-18 years (N=19)	80% Active MT	1	1	Mild headache 5.3%<br>Fatigue 10.5%
Pedapati et al. 2015[94]	10-16 years (N=14)	70% Resting MT	1	1	None
Hong et al. 2015[40]	6-18 years (N=76)	60-90% Resting MT	1	1	Mild headache 6.6%<br>Moderate Arm/hand/other pain 2.6%<br>Numbness/tingling 2.6%<br>Other sensations 2.6%<br>Weakness 1.3%<br>Other 1.3%
Pedapati et al. 2016[95]	11-18 years (N=18)	70% Resting MT	1	1	Mild headache 5.6%
Oberman et al. 2016[97]	16-62 years (N=28)	80% Active MT	1	2	None
Dileone et al. 2016[96]	16-34 years (N=25)	80% Active MT	1	1	None
Abujadi et al. 2018[72]	9-17 years (N=10)	100% Active MT	1	15	None
Purushotham et al. 2018[90]	15 years (N=1)	80% Active MT	1	1	Seizure
Dhami et al. 2019[39]	16-24 years (N=20)	80% Resting MT	1	10	Mild headache 65%<br>Mild chest tightness 5%<br>Mild scalp pain 5%<br>Mild nausea 5%<br>Mild gastrointestinal symptoms 5%<br>Mild nasopharyngitis 5%

Mild restlessness
Mild discomfort
No serious adverse events
No seizures

1.5.2 Anticipated Risks

This study will utilize three daily sessions (spaced 1 hour apart) of bilateral sequential aTBS for 10 daily sessions (5 days per week). Single and paired-pulse TMS will be used for biomarker studies.

Confidentiality

The potential for a breach in confidentiality always exists, specifically with written research data and study databases. However, information that is obtained will be stored in locked file drawers in locked offices; data will have no identifying information to prevent loss of confidentiality. All computers and databases will be password protected with passwords available to limited study personnel. All staff must sign confidentiality certificates. Loss of confidentiality is a potential risk because participants will be asked to disclose their recent medications history, information pertaining to demographics, family history, and psychiatric history. Since code numbers will be given to each participant and data will be stored by code number, the risk of loss of confidentiality will be minimal. Only personnel working on this project at Mayo Clinic will have access to the data. Only data that are pertinent to the study will be collected so that seriousness of loss of confidentiality will be minimized. The alternative to participating in this project is to decline. Participants will be at no risk if they decline to participate.[98]

Information about study subjects will be kept confidential and managed according to the requirements of the Health Insurance Portability and Accountability Act of 1996 (HIPAA). Those regulations require a signed subject authorization informing the subject of the following:

- What protected health information (PHI) will be collected from Subjects in this study
- Who will have access to that information and why
- Who will use or disclose that information
- The rights of a research subject to revoke their authorization for use of their PHI.

In the event that a subject revokes authorization to collect or use PHI, the investigator, by regulation, retains the ability to use all information collected prior to the revocation of subject authorization. For subjects that have revoked authorization to collect or use PHI, attempts should be made to obtain permission to collect scheduled clinical assessments at the end of their scheduled study period.

Data Security and Confidentiality

- Non-electronic source document data will be stored in a locked cabinet in a secure office. Only authorized study staff will have access.
- Electronic data will be stored on a secure database. Only authorized users will have access. All users will have unique identifiers and passwords. Sharing of log-in information is not permitted.

Discontinuation effects from medications:

Certain antidepressants (tricyclic antidepressants), antipsychotics, and stimulant medications will be discontinued if clinically appropriate for study participation as these medications could increase the potential risk for a seizure associated with TMS. Discontinuation effects (changes in mood, increased suicidal thoughts, increased suicidal behaviors, psychotic symptoms, changes in energy level, decreased focus, changes in appetite, and an overall worsening in a subject's clinical condition that requires re-stabilization) are potential risks related to this process. During any medication taper, subjects will be monitored (either telephonically or in person) daily by a physician to monitor and mitigate this risk.

Before any medications are discontinued for the purpose of study participation, the subject's primary clinician and the principal investigator (or another designated board-certified child and adolescent psychiatrist on the research team) will discuss the rationale and potential risks of discontinuation, with the subject and their parent (if younger than 18). If all agree with the rationale, the medication taper will be supervised by both the clinical and research child and adolescent psychiatrist. These clinicians will communicate on a daily basis during the taper. The principal investigator (or another designated board-certified child and adolescent psychiatrist on the research team) will monitor the patient daily with either a face to face visit or telephone call.

Potential Risk of Seizure

There is a small risk of seizure associated with the use of TMS. This is regarded as the most serious adverse event ever reported in TMS and TBS (a type of TMS) studies to date. Seizures have been reported to result from single-pulse TMS but have occurred most frequently with repetitive TMS. The risk for seizure with TBS may be higher than standard TMS, though a recent survey suggests the risk is comparable.[89] Subjects who have a past history of neurological disorders such as a prior stroke or epilepsy have the greatest risk. This incidence is still low (0.6% or less) which is comparable to the reported incidence of seizures with antidepressant medications. The role of TMS in some case reports of seizures is equivocal but the possibility of TMS inducing seizures in subjects without prior neurological pathology cannot be entirely discounted.[86, 99] It is also noteworthy, that in a prior, multicenter trial of TMS involving 301 adult subjects receiving TMS at 120% MT, 3,000 pulses per session, five days a week for 4-6 weeks (this is TMS delivered at the upper limits of recommended safety guidelines) there were no seizures nor elevated rates of serious adverse events.[100, 101] Recently Blumberger and colleagues demonstrated the safety and efficacy of iTBS in adults at 120% MT culminating in FDA clearance for MDD. No seizures were reported in this trial.[35] Reported seizures associated with TMS have also been self-limited and beyond careful monitoring and neurological assessment have not required acute treatment.[85, 102] It is also important to note that there is no evidence that a single seizure, or even a series of induced seizures, makes a subsequent seizure more likely in an otherwise healthy individual. However, it is also true that

there are potential psychosocial consequences of a seizure, including the potential psychosocial impact. There are 3 case reports of seizures in adolescents with depression undergoing TMS treatments.[86, 99, 103] In two of these instances the adolescents were taking psychotropic medications.[86, 99] In one instance the adolescent used alcohol the night before TMS treatment.[86] One case report described a seizure in an adolescent undergoing deep TMS.[103] There is one published report of a seizure induced with iTBS in a 15-year-old with schizophrenia.[90]

With regard to the potential for seizures, all patients will be carefully screened for current or historical factors that may increase the risk of seizures (see Exclusion criteria). The Transcranial Magnetic Stimulation Adult Safety Screen (TASS) will be used to specifically query for any potential seizure risks.[104] Neurologic disease which may increase the risk for seizures is part of the exclusion criteria.[102]

All aTBS sessions will be supervised by a board-certified child and adolescent psychiatrist and all personnel involved will be provided with training in the identification of potential prodromal signs or symptoms of a seizure, particularly the identification of more subtle behavioral events which may herald the occurrence of a frontal lobe seizure, such as changes in level of consciousness or inattention. All aTBS sessions will be conducted in the neurostimulation suite with access to medical facilities and equipment in the event a seizure should occur. Seizure monitoring will be by visual observation. Investigators have the capability to manage a seizure if it were to occur. The Mayo Clinic Neurostimulation Lab has direct access (within minutes) to emergency medicine services.[102]

In the event of a seizure, the supervising physician will initiate appropriate clinical care as defined within the clinical TMS practice. In the management of seizures, attention must be taken to minimize the risk of aspiration, and when possible guiding the patient into the left lateral decubitus position is desirable. Because seizures in the context of TMS are brief (typically <60 seconds) and without serious physical sequelae, efforts will be focused on preventing complications of the seizure rather than initiating any specific medication that is not required unless a seizure is prolonged. If a prolonged seizure (>60 seconds) is identified, appropriate response measures will be initiated which include notifying the hospital-based pediatric rapid response team and escorting the subject to the emergency medicine department upon stabilization (located directly across from the neurostimulation suite). The patient may be provided lorazepam (or equivalent benzodiazepine available in the resuscitation cart) to abort the seizure. To date, status epilepticus has never been described following TMS or TBS.[85, 102]

If a seizure occurs during the study, TMS (aTBS) treatment as part of the study will be discontinued. Ongoing monitoring of mood and neurocognitive symptoms per the study protocol will be offered. Any seizure will be reported to the Mayo Clinic IRB, Data and Safety Monitoring Board (DSMB), the FDA and MagVenture as required per agreements, regulations, and policies.[98]

Cardiogenic syncope (fainting) is another related but rare risk. In the event of fainting, the study team members will ensure that the subject is not injured by falling out of the TMS chair (which would be unlikely). The PI (Dr. Croarkin) or covering study physician will assess the subject immediately and take further action as clinically appropriate. The PI will consult with the subject, subject's parent or guardian, and the subject's primary care physician regarding next steps which may include exit from the study.

Neuropsychological Function after TMS

Several studies have examined the short-term effects of TMS administration across a range of stimulation parameters, and found little or no evidence to suggest a change in cognitive function as a result of TMS administration.[105, 106]

Although there is no evidence to suggest an untoward effect on cognitive function as a result of TMS administration the study team will collect neurocognitive data at baseline, and posttreatment.

Potential Risk of Alteration of Auditory Threshold

During TMS, the coil produces an audible, high energy click. Existing evidence suggests that TMS can result in transient changes in auditory threshold if no precautions are taken (such as wearing earplugs during TMS).[102]

Ear plugs are required for all subjects and study personnel during any TMS or TBS procedure. These are worn routinely during TMS protocols to mitigate the risk of auditory threshold changes. Subjects will undergo audiometry testing at baseline and upon completion the 10-day treatment protocol. The MagVenture device complies with related Occupational Safety and Health Administration (OSHA) guidelines.[35]

Potential for Unintended Behavioral Change (e.g., worsening of depression or induction of mania)

Crying has been reported in some patients receiving left prefrontal TMS. There is no evidence to suggest that a frank worsening of depression may be seen in patients with major depression receiving TMS. On the other hand, mania has been observed in bipolar disorder patients who have received TMS.[99, 102]

Dr. Croarkin or a board certified child and adolescent psychiatrist will be available 24-hours a day to address any concerns subjects or families have regarding changes in behavior. Adverse events are monitored at every treatment visit. Dr. Croarkin or a board-certified child and adolescent psychiatrist will remain in the neurostimulation suite (in a room adjacent to the TMS treatment room) during all TMS treatment sessions.[98, 102]

Suspected treatment-induced mania will be evaluated using the Young Mania Rating Scale (YMRS). A YMRS score of ≥ 20 will prompt administration of the M.I.N.I.(age 18) or M.I.N.I. KID to determine whether or not the subject meets the DSM-5 criteria for mania. A YMRS score of 20 or greater will be considered an adverse event (AE) and will be monitored using the YMRS. The emergence of DSM-5 verified mania will be treated as a serious adverse event (SAE) and will also be monitored using the YMRS.[99, 107]

Potential Risk of Movement of Metallic Objects in the Patient's Body

Because of the brief, but intense local magnetic field induced by the TMS coil, any metallic object in the vicinity of the coil, namely within the head region, may slightly move as a result.[85, 102]

Patients with metallic objects in or near their head are excluded from the study. Patients will be specifically instructed to remove any magnetically sensitive jewelry or other objects near their head.[85, 102]

Potential for Development of Headache or Local Pain at the Site of Stimulation.

TMS administration may result in the development of a transient headache during or after the TMS session, and the development of pain localized near the site of the stimulation coil. These effects are thought to be due to the direct activation of muscles and nerves near the stimulating coil. This is more common with TMS and TBS than with single or paired-pulse TMS.[85, 102]

TMS sessions or measurements will be discontinued immediately in the event of any significant scalp discomfort or pain. Subjects will be counseled on the use of ibuprofen or acetaminophen to relieve pain or discomfort as necessary.[85, 102]

Unimproved Depression or Clinical Deterioration.

Continued depression or worsening of depressive symptomatology is always a risk of depression, even for those treated with medication and/or psychotherapy. Clinical deterioration due to the underlying psychiatric conditions is also a risk.[98, 102]

The study team has designed a number of safeguards focused on depression and suicidality management. Please note that all rating scales (during both the 10-day treatment period and monthly follow up visits) that assess depressive symptoms or suicidality will be reviewed by a child and adolescent psychiatrist on the day of administration. The PI (Dr. Croarkin) is a board-certified child and adolescent psychiatrist. The research team at Mayo Clinic has prior research experience with child and adolescent depressed populations. The research team includes 3 other board-certified child and adolescent psychiatrists with experience delivering investigational TMS to adolescents, extensive experience with suicidal adolescents, and clinical research experience. Should worsening of depression occur, Dr. Croarkin (or a child and adolescent psychiatrist research team member) will follow standard policies and procedures developed by the Mayo Clinic Child and Adolescent Psychiatry Division in the Department of Psychiatry and Psychology. The study psychiatrist will evaluate the adolescent, interview the adolescent's parent, review the adolescent's crisis plan (including contact and emergency numbers), review home safety (confirm no access to firearms, weapons, or large quantities of pills), discuss the risk/benefit ratio of ongoing study participation, and refer to a higher level of care (inpatient hospitalization) as indicated.[98, 102]

During the consent process, subjects and families will be educated about the possibility of significant changes in mood, suicidal thinking, and/or behaviors during the treatment. These changes could include emergence of mania, worsening of depression, and/or suicidal thinking and behaviors. Both the subjects and parent(s) will be told to initiate contact with their study doctor, if the subject experiences any significant mood or behavior changes including suicidal ideation. Principal Investigator contact information is included in the consent/assent document. Furthermore, during the treatment phases of the study, subjects will have frequent contact with the study team child and adolescent psychiatrist (10 daily visits). Ongoing monitoring for worsening of depression and emergence of suicidal ideation and/or behaviors will be evaluated using the CDRS-R, C-SSRS, and clinical global impression-severity scale (CGI-S).[108-110]

Suicidal Behavior

Patients with current suicidal ideation or past suicide attempts are eligible for the study, as ongoing suicidal ideation and behaviors are frequently seen in depressed youth. Therefore, ongoing suicidal ideation or worsening of suicidal ideation and behavior is a risk.[102]

The PI (Dr. Croarkin) is a board-certified child and adolescent psychiatrist with extensive experience treating adolescents with suicidal behavior. The research team includes 3 other board certified child and adolescent psychiatrists. Dr. Croarkin or the research child and adolescent psychiatrist will follow standard policies and procedures developed by the Mayo Clinic Child and Adolescent Division and implement strategies (e.g., comprehensive assessment of depressive symptomatology, assessment of suicidal ideation [inclusive of intent, means, and plan], immediate notification of the adolescent subject's legal guardian, referral for admission to Generose, 1-West, the Mayo Clinic Child and Adolescent Inpatient Psychiatry unit [in cases of immediate lethality]). Implementation of strategies to mitigate suicidal behavior will be tailored to the specific presenting needs of the individual following the procedures detailed above.[98, 102]

Please note that Dr. Croarkin works with a team of Board Certified Child and Adolescent Psychiatrists with expertise in adolescent depression, suicidality, severe psychiatric disorders in childhood, TMS protocols, and clinical research. One of these physicians (in the event Dr. Croarkin is away) or Dr. Croarkin is present in the treatment suite during TMS delivery and available 24 hours a day in the event of emergencies. We have standard operating procedures that the team of psychiatrists are comfortable with and have experience implementing. For example, this team collectively has had experience in hospitalizing suicidal research subjects. The overriding guiding principal for our research team is that subject safety is our central priority. Any decisions regarding clinical monitoring, referral for higher levels of treatment, and ongoing study participation are informed by what will optimize each subject's safety and clinical status. Beyond this, the Mayo Clinic Division of Child and Adolescent Psychiatry (11 board certified child and adolescent psychiatrists) always has an individual on call for psychiatric emergencies. While these colleagues are not co-investigators, they receive training on a regular basis regarding ongoing adolescent research efforts. All members of the Child and Adolescent Psychiatry Division have proficiency and comfort in dealing with psychiatric emergencies. They contact the PI (Dr. Croarkin) or the covering research child and adolescent psychiatrist with any emergency involving a research subject. Research subjects are ultimately offered standard of care interventions based on clinical presentation and need regardless of stage of participation (to include emergent evaluation in outpatient clinic, emergency medicine visits, or psychiatric hospitalization in Mayo Clinic, Generose 1-West, an 18 bed inpatient unit which is located directly below our neurostimulation treatment suite).[69, 102]

Other anticipated risks of TMS and TBS include dizziness, eye pain, facial numbness, facial pain, toothache pain, skin pain, facial muscle twitching, blurred vision.

1.5.3 Potential Benefits

Participating subjects will have MDD and suicidal ideation. Possible benefits to the subject include an alleviation of depressive symptoms and improvement in suicidal ideation from participation in this trial of sequential bilateral aTBS. The study will assess both short (over 10 days of treatment) and long-term benefits over 12 months post treatment. All subjects will

be provided with an intensive, 10-day psychotherapeutic intervention during the trial of sequential bilateral aTBS.[39, 111, 112] This psychotherapeutic program meets and exceeds both local and national standard of care for the treatment of depressed adolescents with suicidal ideation. This anticipated benefit of the study intervention is at least as favorable or potentially superior to alternative clinical approaches. A summary report of examination results can be created and forwarded to the subject's personal physician at the subject's request .

Suicide is a leading cause of death in adolescents and a challenging public health problem.[2, 4] Current clinical treatment approaches are inadequate. The proposed research will develop and examine sequential bilateral aTBS as a treatment for adolescents with MDD and suicidal ideation for use in clinical practice. This treatment approach may provide a safe and effective alternative to standard treatments. This study will also provide important insights into the pathophysiology of depression and suicidal ideation in adolescents. The proposed study is not without risks but does balance risks with an acceptable level of potential benefit for adolescent research subjects. The focus of this research will require ongoing vigilance to ensure subject safety. Prior clinical trials almost universally exclude the proposed study population (adolescents with MDD and suicidal ideation). This unfortunate and repetitive decision is driven by pragmatic concerns and perhaps stigma. As a result, there are unacceptable knowledge gaps and a dearth of brain based treatment options for adolescents with MDD and suicidal ideation. Outcomes related to suicidality in adolescents are not improving. This current study is a first step in reversing this unfortunate pattern as depressed adolescents with suicidal ideation are an impaired, at-risk population, with a great need for effective and safe clinical interventions.[4, 14]

1.6 Anticipated Duration of the Clinical Investigation

The interventional component of the study involves 30 sessions of aTBS over 2 weeks. The research team will then follow adolescents subjects assigned to both study arms (active and sham) for 1 year. Hence, the duration of participation is approximately 13 months.

2 Study Objectives

Suicide is a leading cause of death in adolescents worldwide.[2] In the United States up to 18% of all teenagers seriously contemplate suicide each year.[1, 19] Few prior studies have focused on developing treatments for adolescents with active suicidal ideation. Outcomes related to suicidality in adolescents have not improved in recent decades.[4, 5] There is an urgent, unmet need for brain-based interventions and predictive biomarkers for suicidal ideation in adolescents.[11, 19, 25]

To address these problems we will examine a transcranial magnetic stimulation (TMS) intervention[14, 39] and electrophysiological biomarkers.[25, 81] Accelerated TMS protocols delivered with theta burst stimulation (TBS) induce synaptic plasticity rapidly and overcome

practical limitations of TMS.[47, 121] Our prior work suggests that a standard cortical inhibition TMS electromyography (TMS-EMG) paradigm, long-interval intracortical inhibition (LICI) has utility in assessing suicidal ideation in adolescents.[25, 50] This LICI biomarker is easily collected in clinic. Transcranial Magnetic Stimulation-Electroencephalography (TMS-EEG) LICI and N100 measures may provide more direct measures of cortical inhibition in the prefrontal cortex but have not been adequately studied in adolescents with depression and suicidal ideation.[68, 81] The proposed, double-blind, randomized, sham-controlled study will examine sequential bilateral accelerated TBS (aTBS) for suicidal ideation in adolescents with major depressive disorder (MDD). All subjects (in both arms) will concurrently receive standard of care treatment.[111] Three TBS sessions will be administered daily for 10 days (5 days per week) for a total of 30 sessions. During each session, continuous theta burst stimulation (cTBS) is first delivered to the right dorsolateral prefrontal cortex and then iTBS is delivered to the left dorsolateral prefrontal cortex. The proposed TBS parameters were adopted from prior work in adolescents.[39, 72] The comparison group will receive 3 daily sessions of bilateral sham TBS treatment for 10 days. The research team will collect pre- and post-treatment TMS-EMG LICI-100, TMS-EEG N45, and N100 biomarkers

2.1 Primary Objective

This is a Pilot Study to designed to support future expanded Pivotal Studies of the device.

Primary Aim 1 examines the efficacy of sequential bilateral aTBS compared to sham stimulation for suicidal ideation in adolescents with MDD. The Columbia Suicide Severity Rating Scale (C-SSRS) severity of ideation subscale is the primary acute outcome measure. Additional outcome measures include monthly follow-up C-SSRS severity of ideation subscale scores, number of hospitalizations, and number of emergency department visits related to suicidal ideation over a 12 month follow-up period.

- **Hypothesis 1a**: We expect 10 days of sequential bilateral aTBS will have greater efficacy for improving suicidal ideation in adolescents with MDD (as assessed by the C-SSRS severity of ideation subscale) over the 10-day acute treatment trial compared to sham stimulation.
- **Hypothesis 1b**: We expect that adolescents who receive 10 days of sequential bilateral aTBS will have lower suicidal ideation (as assessed by the C-SSRS severity of ideation subscale) over the 12-month follow-up period compared to sham stimulation.
- **Hypothesis 1c**: We expect that adolescents who receive 10 days of sequential bilateral aTBS will have fewer emergency department visits and fewer hospitalizations related to suicidality during the 12-months after the intervention compared to sham stimulation.

2.2 Secondary Objective

Secondary Aim 2 examines TMS-EMG LICI as a predictive and target engagement biomarker for suicidality in adolescents with MDD.

- **Hypothesis 2a**: We expect that cortical inhibition (as assessed by pre-treatment LICI-100 collected with TMS-EMG) will have an indirect relationship with severity of

suicidal ideation (as assessed by the C-SSRS severity of ideation subscale) in all subjects at baseline.

- **Hypothesis 2b**: We expect that, among the adolescents who receive active sequential bilateral aTBS treatment, there will be an indirect relationship between cortical inhibition (as assessed by LICI-100, TMS-EMG) and suicidal ideation (as assessed by the C-SSRS severity of ideation subscale) over the 10 day acute treatment trial.

An exploratory Aim focuses on developing TMS-EEG measures of cortical inhibition (TMS-EEG N45 and N100) for assessing suicide risk and responsiveness to aTBS in adolescents with MDD.

This is the first study to examine sequential, bilateral accelerated TBS in depressed adolescents with suicidal ideation.[14, 31, 39] Sequential bilateral aTBS and the proposed panel of biomarkers will be rapidly implemented in the clinic. This novel approach will facilitate a critical paradigm shift toward brain-based approaches for assessing and treating suicidal ideation in adolescents with MDD.

3 Study Design

3.1 General Design

This pilot study is a randomized, double-blind, sham-controlled trial of sequential bilateral aTBS in adolescents with depression and suicidal ideation (Figure 7). Three sessions are administered daily for 10 days (5 days per week). During each session, cTBS in which 1800 pulses are delivered continuously over 120 seconds to the right dorsolateral prefrontal cortex (RDPFC) is administered first, followed by iTBS in which 1800 pulses are delivered in 2 second bursts, repeated every 10 seconds for 570 seconds (1800 pulses) to the left dorsolateral prefrontal cortex (LDPFC). The TBS parameters were adopted from prior work, with 3-pulse 50 Hz bursts given every 200 ms (at 5 Hz) with an intensity of 80% of active MT. The comparison group will receive 3 daily session of bilateral sham TBS treatment for 10 days.

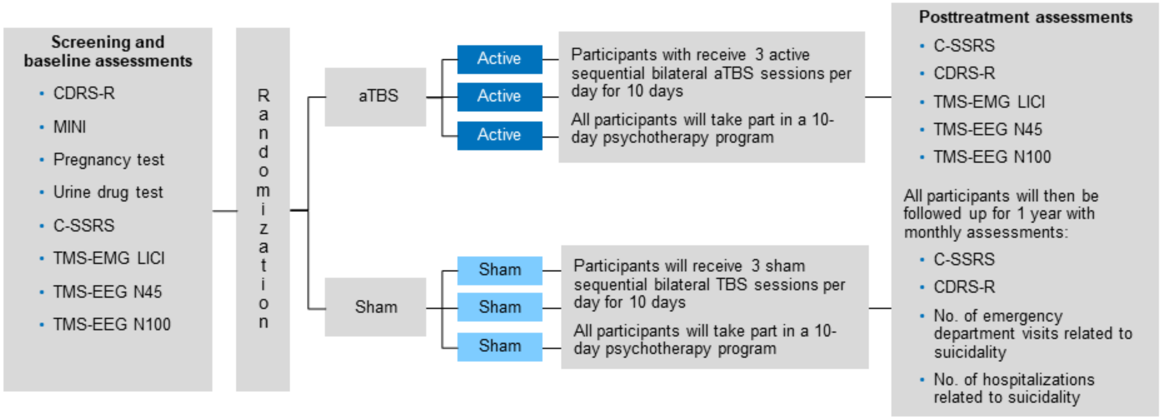

Figure 7. Study Overview

aTBS, Accelerated Theta Burst Stimulation

CDRS-R, Children's Depression Rating Scale Revised

C-SSRS, Columbia-Suicide Severity Rating Scale

MINI, The Mini-International Neuropsychiatric Interview (for age 18) or the Mini-International Neuropsychiatric Interview for Children and Adolescents (for ages 12-17)

TBS, Theta Burst Stimulation

TMS-EEG, Transcranial Magnetic Stimulation with Electroencephalography

TMS-EMG, Transcranial Magnetic Stimulation with Electromyography

All subjects will receive standard of care treatment for depression and suicidal ideation including daily psychotherapeutic skill sessions (Figure 8).[7, 111] The 10-day psychotherapy treatment program was adapted from recent work and input from a consultant (Dr. Kennard).[111] The primary outcome measure is suicidal ideation assessed with the C-SSRS severity of ideation subscale.[110]Depressive symptom severity will be assessed with the CDRS-R.

Session	Participants	Topic
1	Adolescent and Parent	Chain analysis and safety plan
2	Adolescent and Parent	Review adherence, psychoeducation, and safety plan
3	Adolescent	Reasons for living module
4	Adolescent	Mindfulness module
5	Adolescent	Distress tolerance module
6	Adolescent and Parent	Review sessions 3-5 with parent
7	Adolescent	Problem-solving module
8	Adolescent	Socialization and support module
9	Adolescent	Wellness and relapse prevention module
10	Adolescent and Parent	Review work with parent

Figure 8. Psychotherapy Sessions

3.2 Primary Study Endpoints

The primary aim examines the efficacy of sequential bilateral aTBS compared to sham stimulation for suicidal ideation in adolescents with MDD. The Columbia Suicide Severity Rating Scale (C-SSRS) severity of ideation subscale is the primary acute outcome measure. Additional outcome measures include monthly follow-up C-SSRS severity of ideation subscale scores, number of hospitalizations, and number of emergency department visits related to suicidal ideation over a 12 month follow-up period.

3.3 Secondary Study Endpoints

The secondary aim examines TMS-EMG LICI as a predictive and target engagement biomarker for suicidality in adolescents with MDD. An exploratory aim will examine TMS-EEG N45 and N100 measures for assessing suicide risk and responsiveness to sequential bilateral aTBS in adolescents with MDD.

3.4 Primary Safety Endpoints

Data will be collected to determine:

- Incidence of emergency department and hospitalizations related to suicidality over a 12-month period.
- Incidence of adverse events, serious adverse events, and unanticipated adverse device effects.
- Any changes in cognition associated with treatment as assessed by the NIH Toolbox Cognition Battery.
- Any changes in auditory threshold assessments associated with treatment.

4 Subject Selection, Enrollment and Withdrawal

Adolescents with MDD and suicidal ideation will be recruited from the general community and referrals from local providers. Currently Dr. Croarkin directs a research program and outpatient clinic for adolescent mood disorders. It is anticipated that adolescents with MDD and suicidal ideation will also be recruited from an eighteen bed child and adolescent psychiatry unit within Mayo Clinic. Our research group historically has been successful in the recruitment of similar samples. Based on prior experience it is anticipated that 200 patients will be screened and 100 will be enrolled to reach the target sample size of 80 needed to complete this pilot study.

4.1 Inclusion Criteria

Patients are eligible for participation if they are:

- Inpatients or outpatients
- Voluntary clinical patient with the capacity to assent (or consent if 18) to treatment and a parent or legal guardian with the capacity to consent (if younger than 18)
- Biological female or male (nonbinary or other gender identities are not exclusionary)
- 12-18 years of age
- Diagnosed with MDD based on Diagnostic and Statistical Manual for Mental Disorders, 5th edition (DSM-5) criteria with the Mini-International Neuropsychiatric Interview for Children and Adolescents (MINI Kid) in subjects 12-17 years of age; The Mini-International Neuropsychiatric Interview will be used for subjects who are 18 years of age
- In a current episode of MDD with duration of at least 4 weeks but less than 3 years
- Depressive symptom severity as demonstrated by CDRS-R total composite score of Forty or greater and a suicidal ideation score of 3 or more on item 13 of the CDRS-R
- A minimum score of 1 ("wish to be dead") on the C-SSRS severity of ideation subscale
- Demonstrating that depressive symptom severity as evaluated at the screening visit does not improve between screening and baseline by 25% or more
- Eligible for transcranial magnetic stimulation (TMS) based on safety criteria
- On a medically acceptable form of birth control during the 10 day acute treatment course if female and of child bearing potential
- Taking an antidepressant medication if recommended by the referring clinician and agreed upon by parents and patients. Please note that patients are not required to take an antidepressant medication for study participation for practical, ethical, and human subject protection concerns. Medication status and prior treatment resistance will be carefully recorded with the Antidepressant Treatment History Form criteria for relevant statistical considerations.

4.2 Exclusion Criteria

Patients are not eligible for participation if they have:

- Diagnosis of bipolar disorder, anorexia nervosa, bulimia

nervosa, substance use disorders within the past year (with the exception of caffeine and tobacco)

- Lifetime diagnosis of a psychotic disorder confirmed by a research screening interview (including schizophrenia, major depressive disorder with psychotic features, and bipolar disorder with psychotic features)
- Intelligent quotient less than 70 (if there is a clinical concern, subjects will be psychometrically assessed with the Slosson Intelligence Test, Revised)
- Positive urine drug screen at baseline
- Patients with a history of epilepsy or unexplained seizures
- Any family history of epilepsy
- Patients medicated with drugs lowering the seizure threshold (examples: neuroleptic agents and tricyclic antidepressants)
- History of any treatment with electroconvulsive therapy or TMS
- Use of any investigational drug within 4 weeks of baseline
- Prior brain surgery
- Risk for increased intracranial pressure such as a brain tumor
- Life-time history of head trauma with loss of consciousness for greater than 5 minutes duration.
- Any true positive findings on the TMS safety screening form (TASS)
- Pregnant or nursing patients
- Conductive, ferromagnetic, or other magnetic-sensitive metals implanted in the head within. 30 cm of the treatment coil excluding the mouth that cannot be safely removed (examples include cochlear implants, vagus nerve stimulators, deep brain stimulators, implanted electrodes/stimulators, aneurysm clips or coils, stents, bullet fragments, jewelry and hair barrettes)
- Patients with any implanted stimulators or implants controlled by physiologic signals, including VNS, SCS, PNS, defibrillators and pacemakers.
- Patients with neurological conditions that include a history of seizures, cerebrovascular disease, cerebral aneurysm, dementia, movement disorders, having a history of repetitive or severe head trauma, or with primary or secondary tumors in the CNS
- Patients with a history of increased intracranial pressure, history of severe headaches within the previous 1 year, or severe head trauma
- Implanted medication pumps and cardiac pacemakers
- Patients suffering from vascular, traumatic, tumoral, infectious, or metabolic lesions of the brain, even without a history of seizure, or without anticonvulsant medication
-
- Patients with an unstable medical illness (other than depression)
- Inability to adhere to the protocol

4.3 Subject Recruitment, Enrollment and Screening

Adolescent subjects will be recruited through the Mayo Clinic Depression Center and Division of Child and Adolescent Psychiatry. This includes recruitment through outpatient clinics, an

18-bed child and adolescent inpatient psychiatry unit, and the Mayo Clinic Emergency Medicine Department. The Mayo Clinic Health Systems Clinics throughout Minnesota and Wisconsin provide additional recruitment environments. Ongoing and future outreach efforts also involve local treatment facilities in Rochester, St. Paul, and Minneapolis in Minnesota. The principal investigator (Dr. Croarkin) has prior and planned collaborations with the Rochester Public School System. This includes consultation with the school board. With appropriate approvals, recruitment flyers can be disseminated to the parents of all youth attending Rochester Public Schools.

During inpatient hospitalizations, outpatient visits, and emergency medicine department evaluations, a treatment team member will inform the research team and clinical attending physician of a patient's potential interest. The attending physician will review and determine whether or not a discussion of research participation is clinically appropriate. Dr. Croarkin or a research team member will be contacted to speak to the patient and family regarding the study and research participation. If the patient and family are interested, the research team will initiate the informed consent process.

The study team will take proactive steps to foster diversity and inclusion in recruitment efforts with respect to race, ethnicity, and gender. Local (Olmsted County) demographics suggest that of graduating students, 79.2% are White, 5.8% are Asian, 4.73% are African American, and 3.34% are Hispanic or Latino. However, it is anticipated that national patients will travel to Rochester, Minnesota for study participation. The study team will strive to recruit a sample more reflective of affected demographics. The research team will present the study to internal clinical services and external facilities during staff meetings. External efforts will be tailored to foster both community education regarding depression and suicidality in adolescents while establishing referral patterns. Mayo Clinic Institutional Review Board (IRB) approved flyers will be posted within facilities advertising the study. The research team will participate in local and national events such as Suicide Awareness Day, National Depression Day, and National Mental Health Day to facilitate community engagement and subject recruitment. Dr. Croarkin will continue to access and collaborate with relevant offices in the Mayo Clinic Center for Clinical and Translational Science (CCATS) such as the Office of Community Engagement (OCER). The OCER is led by Dr. Christi A. Patten. Dr. Croarkin has ongoing contact with Dr. Patten, Ms. Tabetha Brockman, and Miguel A. Valdez Soto focused on present and future study recruitment. These collaborations provide local speaking opportunities. The study team engages clinical services on a weekly basis as well. When the study opens for enrollment, Dr. Croarkin will attend staff meetings and give presentations at local facilities. The following are three examples:

a. PrairieCare Medical Group
b. Fernbrook Family Center
c. Highland Meadows Counseling Center, Inc.

Dr. Croarkin and the research team have extensive experience with brain stimulation research and studies of adolescents with depression. This prior experience includes the recruitment and retention of adolescents in transcranial magnetic stimulation protocols with 30-60 visits over 6-12 weeks. Our prior experience suggests that the sequential bilateral accelerated theta burst protocol in the present study will facilitate subject retention, as it is a more pragmatic schedule with 10 treatment days total. The Mayo Clinic Neuromodulation research team has the capability of offering treatment sessions over the weekend if necessary to accommodate an

adolescent's or family's schedule. The current protocol also delivers theta burst with an intensity of 80% motor threshold (as compared to standard protocols delivered at a 120% motor threshold). Treatment intensities of 80% motor threshold are more tolerable than 120%. It is anticipated that the current protocol will be more tolerable and preferred by adolescent subjects. The flexibility of scheduling and study theta burst dosing parameters will be highly effective in retaining adolescent subjects for the proposed study.

The primary investigator (Dr. Croarkin) will review documentation of inclusion and exclusion criteria. If not documented in the medical record during the month prior to enrollment, subjects will undergo a urine drug screen and urine pregnancy test (female subjects) during the screening or baseline visit.

4.4 Early Withdrawal of Subjects

4.4.1 When and How to Withdraw Subjects

The PI may withdraw a subject if he believes that for safety reasons it is in the best interest of the subject to be withdrawn. Please refer to section 8.5 (Stopping Rules) for a complete discussion of reasons for subject study withdrawal.

Discontinuation information [e.g., date and the reason(s) for discontinuation] will be recorded in the Subject's Case Report Form (CRF). Subjects who discontinue prematurely should complete the Week 6 assessment procedures within 2 days following their last transcranial magnetic stimulation (TMS) treatment session. See the Schedule of Events for the specific procedures to be performed at this discontinuation visit.

Subjects withdrawn from the study due to an AE will be followed up for 30 days or until resolution. Subjects withdrawn from the study will not be replaced, regardless of the reason for withdrawal. An effort will be made to determine why a subject does not return for the required visits or is dropped from the study. This information will subsequently be recorded on the subject's CRF.

Subjects will be encouraged to remain compliant with all expected study visits. Non-adherence to expected study visits will be documented and may result in removal from the study. This will be clearly discussed during the consent/assent process and reinforced throughout the study through regular screening for issues with compliance.

Dr. Croarkin will assist with clinical referral for all subjects who voluntarily withdraw from the study or are withdrawn by the research team and PI. Specifically, Dr. Croarkin will offer ongoing clinical care or community referrals based on the preference of the subject.

4.4.2 Data Collection and Follow-up for Withdrawn Subjects

In the event that a subject has withdrawn from the treatment phase of the study, the research team will attempt to obtain permission for data collection for the twelvemonth follow-up period.

5 Study Device

5.1 Description

The MagVenture TMS Therapy System will be used under the subject IDE. The device consists of a magnetic stimulator, MagPro X100 with MagOption (K173620), and two magnetic coils, one for MT determination, C-B70, a treatment coil the Cool-B70 A/P coil .

The product code is OBP, and the classification regulations number for the System is 21 CFR 882.5805. All components, except the coils, in the subject pre-sub have previously obtained FDA clearance (K173620).

The MagVenture TMS Therapy System is cleared for treatment of MDD in adult patients who have failed to receive satisfactory improvement from prior antidepressant medication in the current episode. The MagVenture TMS Therapy System is based on the use of intermittent theta burst stimulation, or iTBS. This novel TMS protocol delivers treatment in only 3 min and is based on a patterned TMS paradigm delivered to the Left-Dorsolateral prefrontal cortex (LDPFC) at 120% of resting motor threshold (MT). It consists of bursts of 3 pulses at 50 Hz, with bursts repeated at 5 Hz, in a duty cycle of 2s on, 8 s off, over 3 min 9 s, and a total of 600 pulses. Treatment is administered daily for 5 days per week, for 4-6 weeks.

In the subject pre-sub for an IDE the device will differ from the previously cleared protocol on three important parameters:

- The stimulation protocol,
- The intended use, and
- The MT and treatment coils, C-B70 and Cool-B70 A/P coils, respectively

The TMS therapy will be delivered on both the R- and L-DLFPC, and in this case, treatment will be administered 3 times daily for 10 days in total (5 days per week). The stimulation of the R-DLFPC consists of a continuous theta burst stimulation (cTBS), which has not previously been cleared by the FDA.

The intensity of the newly approved iTBS treatment for adult patients with MDD, is based on the use of an intensity of 120% of MT. However, due to the nature of the accelerated treatment paradigm as well as the age of the target population (ages 13-18 yrs), the intensity will be decreased to 80% MT.

The stimulation parameters proposed here are based on previously published clinical trial literature, showing that bilateral accelerated TBS is both feasible and safe in adult and adolescent patients.

The coil used under the subject IDE, is a coil designed specifically for the use in double-blind, sham-controlled trials. The coil contains both an active site (A) and a sham site (P). The active

site is identical to the previously cleared standard coil, Cool-B70 (K173620) in terms of magnetic field properties. The sham site has a significantly reduced magnetic field, which is less that 5% of that of the active site, enabling a sham stimulation. As the coil contains both an active and a sham site, it is possible to blind the operator as well, as the coil looks identical on both sites.

The keeping of the blind is dependent on the use of a dedicated coil for MT determination. We intend to use the C-B70 coil, which is identical in terms of magnetic properties to the Cool-B70 A/P coil, but is a lightweight, non-cooled coil.

Short description of stimulator and magnetic coils

MagPro Family - Magnetic Stimulators (K173620):

The MagPro family consists of several models based on the same hardware- and software platform. The models which can be used for the TBS protocol are:

- MagPro R30,
- MagPro R30 with MagOption
- MagPro X100
- MagPro X100 with MagOption

The MagPro stimulator is a computerized, electromechanical medical device that produces and delivers non-invasive, magnetic fields to induce electrical currents directed at regions of the cerebral cortex. The magnetic stimulator is connected to a transducer coil which transfers the magnetic stimulation to the cortex.

C-B70 Coil

This coil is used for the determination of the Motor Threshold (MT) of the individual patient. An MT coil is necessary in order to keep the blind of both operator and patient. C-B70 coil has a butterfly type design with two loops of windings. The coil is equipped with a trigger button on the coil handle as well as an intensity wheel, which enable the use of the coil for MT determination. MT needs to be determined for both the right and left motor cortex, when performing bilateral stimulation. The coil is produced with a slightly bent surface. The geometry of the C-B70-coil winding provides an improved coupling of the transducers magnetic field to the brain tissue, thereby reducing the required intensity. The geometry and magnetic field properties are identical to that of the active side (A) of the treatment coil, Cool-B70 A/P.

As the coil is used for a short period and only for MT determination, liquid cooling is unnecessary as the risk of overheating is minimal. The temperature of the internal winding is monitored by a thermal sensor – and if the temperature exceeds 43°C/109°F the MagPro system automatically stops.

Treatment coil, Cool-B70 A/P coil:

This coil is used for both active treatment and sham treatment. The coil has an integrated sham-side that enables double-blind, sham-controlled trial designs, in which both patient and operator are blinded. The coil's active side is identical in terms of magnetic field properties to the C-B70 MT coil presented above, as well as the Cool-B70 standard coil, which is cleared for treatment with iTBS (K173620). The sham side (P) has a significantly reduced magnetic field strength that is less than 5% of that of the active side. The coil is seen in the picture to the right. The coil looks identical on both sides and the MagPro Stimulator will guide the operator as to how the coil should be oriented during treatment of a given patient. This way, the operator will be kept blind towards whether or not the patient receives active or sham treatment.

The Cool-B70 A/P coil is also substantially equivalent to the standard Cool-B70 coil in terms of design elements, such as the use of liquid_cooling in order to maintain the coil cool during treatments. The temperature of the internal winding is monitored by a thermal sensor – and if the temperature exceeds 43°C/109°F the MagPro system automatically stops.

Output waveform

Theta Burst Stimulation (TBS) provides bursts of three individual stimuli at a rate of 50 Hz (i.e. 20 ms apart), repeated at 5 Hz (i.e. 200 ms between burst). This is called a Biphasic Burst with 3 pulses.

Biphasic Burst (Theta Burst)

The biphasic burst waveform is able to provide a powerful stimulation. Biphasic Burst should be selected with 5 pulses in each stimulation with very low IPI (Inter Pulse Interval). The biphasic burst mode can be used to define Theta Burst stimulations. The MagVenture TMS Therapy System has undergone verification tested to ensure that the intensity of individual stimuli is equal throughout the course of treatment. It has been demonstrated that at the relevant TBS intensities required in the treatment setting, the individual intensity of the three stimuli is equal and kept constant.

Instructions for Use

For your convenience we have submitted a copy of the Instructions for use (IFU) pertaining to The MagVenture TMS Therapy System that was cleared in August 2018, K173620. This is the standard iTBS 3 min protocol that the protocol in the present pre-sub builds on.

We have also submitted the IFU pertaining to the use of the Cool-B70 A/P coil.

Before submission of an IDE pertaining to this trial, a full IFU will be made available for this trial specifically.

List of components

List of components (Table 2 that make up the system in this pre-sub along with the specific FDA submission numbers for each component that received a prior 510(k) clearance.

Table 2 MagVenture TMS Therapy System Components

Components	Prior FDA clearance	Specific FDA 510(k) submission number
R30, Stimulator	Yes	K173620, K150641, K171481
X100, Stimulator	Yes	K173620, K150641, K171481
X100 with MagOption, Stimulator	Yes	K173620, K150641, K171481
C-B70, coil	No	Substantially equivalent to Cool-B70, cleared in K173620
Cool-B70 A/P coil	No	Substantially equivalent to Cool-B70, cleared in K173620
Cooler Unit	Yes	K173620, K150641, K171481
Trolley	Yes	K173620, K150641, K171481
Isolation Transformer	Yes	K173620, K150641, K171481
Vacuum Pump and Pillow*	Yes	K173620, K150641, K171481, K171967*

Pillow case	Yes	K173620, K150641, K171481, K172667
Super Flexible Arm	Yes	K173620, K150641, K171481
Treatment Chair	Yes	K173620, K150641, K171481, K171967*
Cap	Yes	K173620, K150641, K171481, K172667
Software v. 7.2	Yes	K173620

5.2 Method for Assigning Subjects to Treatment Groups

Randomization: Subjects will not be randomized until all entry (inclusion and exclusion) criteria have been met and all pre-study procedures have been satisfactorily completed. The study biostatistician will generate a randomization schedule using a random number program (e.g., SAS software via PROC PLAN).[124] Subjects will be randomly assigned in a 1:1 ratio to receive one of the two treatment regimens (either 3 daily sequential bilateral aTBS sessions in the active arm or 3 daily sham sessions) for 10 days.

5.3 Preparation and Administration of Investigational Device

Preparation and administration of the TMS treatments will be consistent with procedures outlined by the MagVenture user manual and user training documentation.

5.4 Subject Compliance Monitoring

Non-adherence to the assigned treatment regimen is defined as missing ≥2 treatments days, or missing more than 20% of the total number of treatment sessions (i.e. 6 sessions) occurring during the 2 weeks of treatment as outlined in the schedule of events.

5.5 Prior and Concomitant Therapy

Reasonable efforts will be made to determine all somatic therapies for depression received by the participant in the past 2 years. All other medications and therapies received within six months of study enrollment will also be determined. All relevant information will be recorded on the Participants CRF. The ATHF will be completed to quantify treatment history.

During the trial, subjects will continue to take antidepressant medication if recommended by the referring clinician. Please note that subjects are not required to take an antidepressant medication for study participation for practical, ethical, and human subject protection concerns. Medication status and prior treatment resistance will be carefully recorded with the ATHF for relevant statistical considerations. The research team will collect information on subsequent pharmacological treatments during the 1-year follow up period.

Zaleplon, zolpidem, or zopiclone (1 dose nightly) as needed for treatment emergent insomnia or lorazepam (up to 2 mg daily) for treatment emergent anxiety, may be administered for up to 10 doses during the treatment phase of the study. The use of alternative hypnotics or anxiolytic compounds requires prior approval from the PI (Dr. Croarkin). Hormonal contraceptives are allowed. Short-term treatments for headaches, allergies, colds, and flu symptoms will be allowed during. All questions regarding the acceptability of specific medications must be approved by the PI/Sponsor (Dr Croarkin).

5.6 Packaging and Labeling

The MagVenture device will be clearly labeled with the following warning:

"CAUTION – Investigational Device. Limited by Federal (or United States) law to investigational use"

5.7 Masking/Blinding of Study

Blinding: The use of the MagVenture Cool-B70 A/P coils facilitates blinding of the operator and subjects receiving aTBS or sham treatments. The study team members completing clinical rating scales will also not be allowed in the treatment suite during TBS treatment sessions and will be blinded to treatment assignment. A pre-post treatment expectancy and experience form will ask subjects and parents to make a guess regarding treatment arm (active or sham). Blinded study team members will also be asked to guess treatment assignments for each subject.

5.8 Receiving, Storage, Distribution and Return

5.8.1 Receipt of Investigational Devices

The MagVenture device will be delivered to the Principal Investigator/Sponsor and installed by MagVenture.

Upon receipt of the MagVenture TMS Therapy System and study treatment supplies, an inventory will be performed and a device accountability log completed by designated study staff. The designated study staff will count and verify that the shipment contains all the items noted in the shipping invoice. Any discrepancies or damaged or unusable devices in a given shipment will be documented in the device accountability log. The PI will notify MagVenture immediately of any discrepancies or damaged or unusable products.

5.8.2 Storage

The supplies and disposables required for each treatment will be stored in a supply station separate from clinical practice stock.

5.8.3 Distribution of Study Device

The study team will maintain a local device accountability log noting treatments received by each participant with the device. Reconciliation and audits will take place at least annually.

5.8.4 Return or Destruction of Study Device

The MagVenture Devices that are loaned to the study site for purposes of this study will be returned to MagVenture at conclusion of the study. Remaining disposables and related study equipments that are remaining in the site study research inventory at the completion of the study will be packaged and returned to MagVenture.

6 Study Procedures

Experimental Design: This is a randomized, double-blind, sham-controlled trial of sequential bilateral aTBS in adolescents with depression and suicidal ideation (Figure 7). Three sessions are administered daily for 10 days (5 days per week). During each session, cTBS in which 1800 pulses are delivered continuously over 120 seconds to the right dorsolateral prefrontal cortex (RDPFC) is administered first, followed by iTBS in which 1800 pulses are delivered in 2 second bursts, repeated every 10 seconds for 570 seconds (1800 pulses) to the left dorsolateral prefrontal cortex (LDPFC). The TBS parameters were adopted from prior work, with 3-pulse 50 Hz bursts given every 200 ms (at 5 Hz) with an intensity of 80% of active MT. The comparison group will receive 3 daily session of bilateral sham TBS treatment for 10 days.

All subjects will receive standard of care treatment for depression and suicidal ideation including daily psychotherapeutic skill sessions (Figure 8).[7, 111] The 10-day psychotherapy treatment program was adapted from recent work and input from a consultant (Dr. Kennard).[111] The primary outcome measure is suicidal ideation assessed with the C-SSRS severity of ideation subscale. Depressive symptom severity will be assessed with the CDRS-R.

Table 3. Schedule of Events						
**Study Activity**	**Screening**	**Baseline**	**Daily aTBS and psychotherapy sessions**	**Posttreatment (End of 10-day treatment)**	**Monthly Follow-up Visits**	**Comments**
Consent and Assent	X					
Transcranial Magnetic Stimulation Safety Screen (TASS)	X					
Antidepressant Treatment History Form (ATHF)	X					
Medical History	X					
Demographics Questionnaire	X					
Physical Exam	X					
Vital Signs	X			X		
Pubertal Development Scale (self-assessment)	X					
Urine Drug Screen	X					
Urine Pregnancy Screen (if applicable)	X					
Auditory thresholds		X		X		
Mini-International Neuropsychiatric Interview for Children and Adolescents (MINI Kid) Adult version used for subjects who are age 18	X					
NIH Toolbox Cognition Battery		X		X		
The Brief Affective Personality Scale	X					
Young Mania Rating Scale (YMRS)	X	X	X	X	X	Collected weekly during 10-day treatment period
Childhood Depression Rating Scale – Revised (CDRS-R)	X	X	X	X	X	Collected weekly during 10 day treatment period
Columbia Suicide Severity Rating Scale (C-SSRS)	X	X	X	X	X	Collected daily during 10 day treatment period
Childhood Trauma Questionnaire	X					
Beck Depression Inventory-II (BDI-II)	X	X		X	X	Collected weekly during 10 day treatment period

Pre/Post Treatment Expectations and Experience Questionnaire (Adolescent and Parent)		X		X		
Clinical Global Impressions Severity Scale (CGI-S)	X	X	X	X	X	Collected weekly during 10 day treatment period
Concurrent Medications	X	X	X	X	X	
Adverse Events	X	X	X	X	X	
Serious Adverse Events	X	X	X	X	X	
TMS Neurophysiology Measures (TMS-EMG LICI-100 and TMS-EEG LICI-100 and N100)		X		X		
Case Report Forms	X	X	X	X	X	
Concurrent Psychotherapy	X	X	X	X	X	
Concurrent Medications	X	X	X	X	X	
Hospitalizations Related to Suicidality Noted					X	
Emergency Department Visits Related to Suicidality Noted					X	

6.1 Visit 1

Table 3 describes the schedule of events. Table 4 describes all study assessments. The first visit is a screening visit. Informed consent and assent will be completed. A medical history, physical exam, vital signs, urine pregnancy test (if applicable) and urine drug screen will be completed. The subject's parent or guardian will not be told the results of the pregnancy test without the subject's permission. But if the study physician believes that being pregnant may cause serious health problems, they may need to tell the parent or guardian the test results. Patients with a positive urine drug screen that are not due to prescribed drugs are not eligible for study participation. The study physician will ask if the results can be shared with the parent or guardian. This information will be kept confidential if the subject chooses not to share the drug test results with the parent or guardian. The results of the urine drug test will not become pat of the medical record, but the results will remain in the study record. Additional screening assessments include: the Transcranial Magnetic Stimulation Safety Screen (TASS), Antidepressant Treatment History Form (ATHF), pubertal development scale (PDS), Mini-International Neuropsychiatric Interview (MINI Kid for ages 12-17 and MINI for age 18), Young Mania Rating Scale (YMRS), Children's Depression Rating Scale Revised (CDRS-R), Columbia Suicide Severity Rating Scale (C-SSRS), Beck Depression Interview-II (BDI-2), Clinical Global Impressions Severity Scale (CGI-S), concurrent medications, adverse events , serious adverse events, demographics questionnaire, case report forms, and concurrent psychotherapy. The screening visit can occur on the same day or across 2 consecutive days based on participant preference.

6.2 Visit 2

After eligibility is confirmed, additional assessments will be completed (Table 3 and 4). These assessments include auditory thresholds, NIH Toolbox Cognition Battery, YMRS, CDRS-R, C-SSRS, BDI-2, Pre/Post Treatment Expectations CGI, concurrent medications, adverse events, serious adverse events, case report forms, concurrent psychotherapy. TMS neurophysiology measures are collected. The participant is randomized to the active aTBS group or sham aTBS. Coil localization (Beam F3) and motor threshold procedures are completed.[125] The participant will complete day one of aTBS treatment (either 3 active sequential bilateral TBS or 3 bilateral sham treatments).

6.3 Daily aTBS sessions. All study subjects will receive 30 sessions of sequential bilateral aTBS or sham stimulation. The research team will use the Beam F3 method for coil localization of the RDPFC and LDPFC. Prior studies have demonstrated the validity and reliability of the Beam F3 method for coil localization in comparison to magnetic resonance guided approaches. Prior work demonstrated that the Beam F3 method is a feasible, valid, and reliable method for coil localization for TMS protocols in adolescents.[78, 126] Subjects in the active arm will receive 11.5 minutes sessions of sequential bilateral aTBS at 80% active MT, 3 times daily (1 hour apart) for 10 days in total (5 days per week). Each session includes TBS in which 1800 pulses are delivered continuously over 120 seconds to RDPFC followed by iTBS in which 1800 pulses are delivered in 2 second bursts, repeated every 10 seconds for 570 seconds (1800 pulses) to the LDPFC. Subjects assigned to the sham arm will receive identical treatment but with a magnetic field strength less than 5% of the active treatment. The stimulation parameters proposed here are adapted from initial work with adolescents and previously published clinical trial literature, showing that bilateral accelerated TBS is both feasible and safe for patients. Broadly, theta burst stimulation is delivered with 3-pulse 50 Hz bursts given every 200 ms (at 5 Hz) with an intensity of 80% of active MT. The MagVenture TMS Therapy System will be used in accordance with a FDA investigational device exemption (IDE). The device consists of a magnetic stimulator, MagPro X100 with MagOption and two magnetic coils. The C-B70 coil is for MT determination. The Cool-B70 A/P coil is the treatment coil. The Cool-B70 A/P coil that will be used was specifically designed for double-blind, sham-controlled trials. The coil contains both an active site (A) and a sham site (P). The active site is identical to a previously FDA cleared standard coil, Cool-B70 in terms of magnetic field properties. The sham site has a significantly reduced magnetic field, which is less than 5% of that of the active site, enabling a sham stimulation. As the coil contains both an active and a sham site, it is possible to blind the operator as well, as the coil looks identical on both sites The coil has somatosensory electrodes to enhance the blinding of participants and operators. The electrodes are placed on the RDLPFC and LDLPFC under the coil during stimulation. The keeping of the blind is dependent on the use of a dedicated coil for MT determination. The C-B70 coil will be used for MT determination. The C-B70 coil is identical in terms of magnetic properties to the Cool-B70 A/P coil, but is a lightweight, non-cooled coil.[35]

***Concurrent Psychotherapeutic Sessions*.** All subjects will receive psychotherapeutic treatment which meets and exceeds the local standard of care. This will consist of daily psychotherapeutic sessions during the 10-day course of sequential bilateral aTBS or sham treatment. The psychotherapeutic treatment protocol was adapted from prior NIMH funded work and a grant funded intensive outpatient program at UT Southwestern Medical Center Dallas.[7, 111] Dr. Kennard (consultant) assisted in the proposed adaptation and will provide ongoing input to Dr. Leffler. Dr. Leffler will provide local supervision and oversight of the 10-day psychotherapy program. The research team will collect information on subsequent psychotherapy treatments during the 1-year follow up period.[98, 127]

6.4 Methods and Measures

Table 4 describes the assessments for the study.

**Table 4. Description of Assessments**	
**Assessment**	**Description**
Antidepressant Treatment History Form (ATHF)	Collected at baseline to systematically document prior psychotropic medication exposure and for quantification of treatment resistance (number of failed antidepressant trials with an adequate dose and duration).[113]
Adverse events	Subjects will be evaluated for adverse events at each visit. Serious adverse events (SAEs) will be reported as they occur to the Mayo Clinic IRB and USA FDA (as per regulations). Systematic data collection focused on adverse events will include the Pediatric Adverse Event Rating Scale and Physical Symptom Checklist.[114, 115]
Auditory thresholds	Subjects will undergo audiometry testing at baseline and upon completion of 10 days of sequential bilateral aTBS.
Beck Depression Inventory-II	This is a validated, 21-item, self-report measure with a range of 0-63. Collected at screening, baseline, weekly during the 10-day treatment and then monthly for 1 year follow-up period.[116]
Brief Affective Neuroscience Personality Scale	This is a validated 33-item, self-report measure collected at screening only to assess behavioral characteristics in 6 affective neurobiological systems (play, seek, care, fear, anger, and sadness)
CDRS-R – Childhood Depression Rating Scale – Revised	This is a validated, 17-item, clinician-rated tool to assess severity of depression. Parents provide input into 14 of the items. Total possible scores range from 17 to 113. Collected at screening, baseline, and weekly (after 5 treatments and posttreatment) during 10-day treatment and then monthly for 1 year follow-up period.[109]
CGI-S– Clinical Global Impressions Severity Scale	A standard measure of symptom severity and treatment response. Collected at screening, baseline, weekly (after 5 treatments and posttreatment) during 10-day treatment and then monthly for 1 year follow-up period.[108]
C-SSRS – Columbia Suicide Severity Rating Scale	This is a validated, clinician-rated tool collected to assess lifetime and ongoing suicidal ideation and behavior at screening, baseline, daily during the 10 day treatment, then monthly for 1 year follow-up period. The severity of ideation subscale is a 5-point ordinal scale that is the primary clinical outcome measure for this study.[110]
Demographics Questionnaire	This is a self-reported questionnaire that collects the subjects gender, race, ethnicity, sexual orientation and educational levels of parents.
Mini-International Neuropsychiatric Interview for Children and Adolescents (MINI Kid) and Mini-International Neuropsychiatric Interview (MINI)	A structured psychiatric interview (MINI Kid for ages 12-17 and MINI for age 18 and). Collected at baseline to confirm diagnostic inclusion criteria and rule out diagnostic exclusion criteria.[117, 118]

NIH Toolbox Cognition Battery	http://www.healthmeasures.net/index.php<br>Cognition measures: http://www.healthmeasures.net/explore-measurement-systems/nih-toolbox/intro-to-nih-toolbox/cognition<br>**The NIH Toolbox Cognition Battery,** recommended for ages 7+, consists of tests of multiple constructs. It yields individual measure scores and the following summary scores: Education, Handedness, Attention (Flanker Inhibitory Control and Attention Test), Episodic memory (Picture Sequence Memory Test, and Auditory Verbal Learning Test), Executive Function (Dimensional Change Card Sort Test, Flanker Inhibitory Control and Attention Test), Language (Picture Vocabulary Test, Oral Reading Recognition Test), Processing Speed (Pattern Comparison Processing Speed Test), Working Memory (List Sorting Working Memory Test). Collected at baseline and posttreatment.[119]
Pre/Post Treatment Expectations and Experience Questionnaire (Adolescent and Parent)	This questionnaire assesses patient and parent impressions, expectations, and the integrity of blinding by asking adolescents and parents to make a guess regarding study arm assignment. Collected at baseline and posttreatment.
Pubertal Development Scale and Tanner Staging	A self-report questionnaire and line-drawings used to evaluate level of pubertal development. Collected at screening.[120]
TASS – Transcranial Magnetic Stimulation Safety Screen	This safety screen is completed prior to TMS for confirmation patient safety. Collected at baseline.[104]
Vital Signs	(Pulse, blood pressure, temperature, height, and weight) will be collected at screening and posttreatment.
YMRS-Young Mania Rating Scale	This is an 11-item measure used to assess for manic symptoms. The YMRS scale will be completed at baseline and repeated during the course of study participation if symptoms of mania occur in a study subject. If the YMRS is positive, A MINI/MINI-Kid will be repeated to evaluate for mania. If the study subject meets full DSM-5 criteria for mania or hypomania he or she will be discontinued from the study and followed clinically.[107]
TMS Neurophysiology Measures	TMS-EMG (LICI-100)and TMS-EEG (LICI-100 and N100) neurophysiology measures will be collected in adolescent subjects at baseline and after the final day of sequential bilateral aTBS.[25, 81]

Clinical and Standardized Assessments: At screening subjects will undergo a urine drug screen, urine pregnancy test, MINI-Kid or MINI interview, Brief Affective Neuroscience Personality Scale, CDRS-R, C-SSRS, Childhood Trauma Questionnaire, Clinical Global Impressions Scale (CGI), Safety Screening for TMS, Young Mania Rating Scale, Adverse Event Rating Scales, Medical History, Physical Exam, Vital Signs, Pubertal Development Scale, and the Beck Depression Inventory-II (BDI-II).[116] At the baseline visit subjects will repeat the CDRS-R, C-SSRS, CGI, BDI-II, and Adverse Rating Scales. Other baseline assessments include the NIH Toolbox Cognition Battery, Auditory Threshold Testing, and a Pre-treatment Expectation and Experience Questionnaire. Subjects will undergo TMS-EMG testing and TMS-EEG testing at the baseline line visit. The C-SSRS (suicidal ideation severity subscale, which is the primary outcome measure) will be assessed daily for the 10 treatment days. The CDRS-R, CGI, and BDI-II will be collected weekly during the treat period (baseline, after 5 treatments, and posttreatment). After the final day of treatment, the subjects will complete the C-SSRS, CDRS-R, CGI, BDI-II, Post-treatment Expectation and Experience Questionnaire and the NIH Toolbox Cognition Battery. Vital signs will be collected at this posttreatment visit. Subjects will undergo posttreatment TMS-EMG and TMS-EEG testing. Follow-up, post-treatment assessments will be collected each month over the 12-month follow-up period. These monthly assessments will include the C-SSRS, CDRS-R, CGI, BDI-II, number of hospitalizations related to suicidality, and number of emergency department visits

related to suicidality. Concurrent medications, concurrent psychotherapy, and adverse events will be collected at every study visit.

Neurophysiological TMS: Neurophysiological TMS procedures (LICI-100 is the primary outcome measure) will be conducted at Mayo Clinic with our standard protocols that have been published previously.[25, 59] Relaxation will be monitored with audio feedback. Surface EMG readings are collected from the abductor pollicis brevis (APB) muscle. The TMS-EMG panel includes LICI, SICI, intracortical facilitation (ICF), and the cortical silent period (CSP) counterbalanced to avoid order effects.[59] Note that SICI, ICF, and CSP are collected for exploratory analyses and are not the focus of the present grant specific aims. The primary outcome measure is LICI-100 (LICI with an ISI of 100 ms). The TMS-EEG recordings (for LICI-100 and N100 as examined in prior studies of adults) will be acquired through a 64-Channel EEG (EGI Foundation) System in accordance with prior published protocols (that have yielded high test-retest reliability with an intraclass correlation coefficient greater than 0.9).[128, 129] In light of recent controversies and publications, the protocol will include a realistic sham (scalp and auditory stimulation) to account for peripheral multisensory stimulation. A foam sponge will be placed between the coil and the EEG cap to reduce tactile artifact. Foam earphones and white noise cancellation will be used to mask the auditory click that occurs with TMS, thereby mitigating auditory artifact.[64-66, 130]

TMS-EMG: The research team will ensure participants maintain relaxation during the procedures with audio feedback tests. TMS stimulation will be applied to the hand area of the contralateral cortex with a figure-of-eight magnetic coil (with a coil diameter of 70 mm on each loop) using the Magstim 200 magnetic stimulator device. The resting MT is defined as the stimulation intensity which elicits a motor evoked potential (MEP) of > 50 microvolts in 5 of 10 trials with a relaxed APB muscle. For LICI measurements, a suprathreshold conditioning stimulus (CS) precedes a suprathreshold test stimulus (TS). The CS and TS are both set to 120% of resting MT, which is calibrated to produce an average MEP of 0.5 to 1.5 millivolt peak-to-peak amplitude in the contralateral APB muscle. Conditioning stimuli are delivered to the motor cortex prior to the TS in one of three random ISIs at 100 ms, 150 ms, and 200 ms along with single TS randomly interspersed within the three random ISIs. Changes in TS MEP amplitude of each ISI are expressed as a percentage of the mean unconditioned MEP amplitude.[59, 70]

TMS-EEG: TMS-EEG data for the exploratory aim will be collected with standard procedures published previously. Subjects will undergo LICI-100, N45, and N100 in the LDPFC with a 64-Channel EEG (EGI Foundation), TMS compatible MicroCel net in place. The research team will ensure participants maintain relaxation during the procedures with audio feedback tests. TMS stimulation will be applied to the hand area of the contralateral cortex with a figure-of-eight magnetic coil (with a coil diameter of 70 mm on each loop) using the Magstim 200 magnetic stimulator device. The TMS coil is held tangentially on the head with the handle backward at 45 degrees laterally from the midline to determine resting MT. The optimal coil position is located by moving the coil in 1-cm increments over the motor cortex. The resting MT is defined as the stimulation intensity which elicits a motor evoked potential (MEP) of > 50 microvolts in 5 of 10 trials with a relaxed APB muscle. For DLPFC stimulation, the TMS coil is localized over the F5 electrode in line with AF3. This approach has been shown to yield quality data in adults and magnetic resonance guided localization is not practical in the context of this exploratory work. The EEG electrodes will be referenced to Cz. In line with recent

collaborative efforts to improve the validity and reliability of TMS-EEG biomarkers, data collection will include a realistic sham condition (scalp and auditory stimulation). These measures will ensure the appreciation of sensory event-related potentials and related impact on TMS-EEG N45 and N100 recordings. Recordings will be in DC mode with 1000-Hz low pass filter (48 dB/octave) and a sampling rate of 20 kHz. These recording parameters have been shown to avoid amplifier saturation and minimize TMS-related artifact.[64, 81]

For LICI measurements, a suprathreshold conditioning stimulus (CS) precedes a suprathreshold test stimulus (TS). The CS and TS are both set to 120% of resting MT, which is calibrated to produce an average MEP of 0.5 to 1.5 millivolt peak-to-peak amplitude in the contralateral APB muscle. Conditioning stimuli are delivered to the motor cortex prior to the TS in one of three random ISIs at 100 ms, 150 ms, and 200 ms along with a single TS randomly interspersed within the three random ISIs. Changes in TS MEP amplitude of each ISI are expressed as a percentage of the mean unconditioned MEP amplitude. For N100 response data collection, mean single pulse data will be collected for each electrode for the TMS-evoked potential. The N100 peak value is then extracted throughout electrodes from the negative in closest proximity to 100 ms.[64, 81]

Custom MATLAB scripts and EEGLAB toolbox will be used for EEG preprocessing. The EEG signal will be resampled from 20 kHz to 1000 Hz and epoched into trials with data from 1000 ms before to 1000 ms after the onset of each test pulse. Artifact free periods will be used to baseline correct and remove TMS artifact. Channels and trials with amplitude and frequency outliers will be removed. Independent component analyses will be applied to the data. Components with TMS decay artifacts will be removed. The signal will be band-pass filtered to the range of physiologically meaningful EEG (1-80 Hz) along with a notch filter set to 60 Hz to remove line noise. Other outlier trials and channels will be removed with a second independent component analysis to remove eye blinks, electrode artifacts, muscle artifacts, and sensory artifacts. Missing channels will be interpolated with linear surface interpolation. All channels will be average referenced.[64, 81]

7 Statistical Plan

7.1 Sample Size Determination

Sample Size Estimation and Power Analysis for the Primary Aim of the Study

We established the sample size for the Primary Aim/Outcome/Hypothesis 1a (suicidal ideation over 10 days of acute sequential bilateral aTBS treatment) by incorporating a repeated measures design, with assumptions about potential participants (e.g., effect size/odds ratio), based on general guidance from a recent study that evaluated the effect of bilateral rTMS vs. sham stimulation on suicidal ideation in adult patients with treatment-resistant MDD.[31] The results of the *a priori* sample size estimation and power analysis suggest that a sample size of 35 participants per group (N=70) achieves 80% power, at a 0.05 alpha level (two-tailed), to detect an overall odds ratio of 2.85 (sequential bilateral aTBS vs. Sham stimulation on lower-ordered intensity of suicidal ideation CSSR-S subscale scores—less suicidal ideation) in a 2 (between-subjects) group randomized design with 10 (within-subjects) repeated measurements of the primary ordinal outcome (suicidal ideation), when the proportion (or incident rate) of suicidal ideation from the sham group at the end of the 10-

day trial ≥0.50, and the assumed within-subject correlation between observations (suicide ideation) on the same subject is ≤0.50 (0.50 was based on guidance from our preliminary TMS work).[14] We note that the posited odds ratio of 2.85 for the current application is a bit more conservative than what was reported in the Weissman, et al. (2018) study of bilateral TMS vs. Sham stimulation on suicidal ideation in adult patients with treatment-resistant MDD (odds ratio=3.03).[40]

We anticipate some dropouts (~10% based on treatment discontinuation results from our previous rTMS studies).[14, 78, 131] Thus, ~80 participants will be enrolled to allow for this expected rate of attrition with the intent of capturing a range of evaluable data for 70 participants. The Power Analysis and Sample Size (PASS) 2019 software, version 14.0.15, was used to carry out the sample size and power analysis (PASS 2016, NCSS, Inc., Kaysville, Utah).[132]

We recognize that to definitively evaluate the effect of sequential bilateral aTBS treatment on suicidal ideation in youth a sample size larger than 70 may be necessary. The treatment effect of sequential bilateral aTBS has not been previously studied in suicidal, depressed youth, we have no previous findings from a youth population to guide effect size selection for the present effort, and the current study would be the first of its kind to examine this treatment effect in depressed youth with suicidal ideation. The selection of 70 participants was, in part, resource-driven. The sample size is designed, in part, to detect a moderate effect and to establish a *proof of concept* so as to expand to a subsequent, larger-scale multi-center study. Nevertheless, the current proposal will permit an evaluation of whether receiving sequential bilateral aTBS shows initial evidence of an effect on improvement in suicidal ideation in a depressed adolescent population.

7.2 Statistical Methods

Statistical Methods

All statistical analyses will be carried out using SAS software, version 9.4. The level of significance will be set at $\alpha = 0.05$ (two-tailed) and, to address multiple testing (where applicable), p-values will be adjusted using the False Discovery Rate.[133]

Primary Aim

Data Analysis Plan for Primary Aim 1 (Hypotheses 1a−1c)

Primary Outcome for Aim 1: Suicidal Ideation during the 10-day acute Trial Period

Hypothesis 1a: We expect 10 days of sequential bilateral aTBS will have greater efficacy for improving suicidal ideation in adolescents with MDD (as assessed by the C-SSRS severity of ideation subscale) over the 10-day acute treatment trial compared to sham stimulation.

Suicidal Ideation (as measured by the C-SSRS severity of ideation subscale) over the 10-day acute sequential bilateral aTBS trial period will be the primary ordinal outcome measure. The acute change over time (10 days) in Suicidal Ideation will be compared between the two treatment groups (sequential bilateral aTBS vs. sham stimulation) using an ordinal logistic regression model within a Generalized Estimating Equation (GEE) framework. The logistic model will contain fixed effects terms for treatment, time, treatment × time interaction along with baseline C-SSRS severity of ideation subscale as a covariate. Age, sex, pubertal status, antidepressant medication status, and depression severity (CDRS-R total as a time-varying covariate) will also be included as covariates in the model. Simple treatment group effects in each time period will also be assessed. [see sensitivity analysis section below for missing not at random (MNAR) mechanism].

Secondary Ordinal Outcome for Aim 1: Suicidal Ideation during the 12 month Follow-up

Hypothesis 1b: We expect that adolescents who receive 10 days of sequential bilateral aTBS will have lower suicidal ideation (as assessed by the C-SSRS severity of ideation subscale) over the 12-month follow-up period compared to sham stimulation.

Suicidal Ideation (as measured by C-SSRS severity of ideation subscale) will be assessed at monthly follow up visits over a 12-month period after the intervention. An ordinal logistic regression model within a GEE analysis framework similar to that described above for Hypothesis 1a will be used here to estimate suicidal ideation over the 12 month follow-up period. In addition to the covariates specified above in Hypothesis 1a, we will also consider the inclusion of pharmacotherapy and/or psychotherapeutic intervention received over the follow-up period as additional covariates.

Secondary Count Outcome for Aim 1: Emergency Department visits and Hospitalization visits during the 12 month Follow-up

Hypothesis 1c: We expect that adolescents who receive 10 days of sequential bilateral aTBS will have fewer emergency department visits and fewer hospitalization visits related to suicidality during the 12-months after the intervention compared to sham stimulation.

Emergency department visits and hospitalization visits related to suicidality will be assessed at monthly follow up visits over a 12-month period after the intervention. A separate Poisson

(or negative binomial) mixed model analysis will be used to estimate the number of emergency department visits and hospitalization visits, respectively, over the 12 months after intervention for those who received sequential bilateral aTBS vs. those who received sham stimulation. The model will contain fixed effects terms for treatment, time, treatment × time interaction, and the same covariates identified in hypotheses 1a and 1b above. Simple treatment group effects in each time period will also be assessed. Maximum likelihood estimation, Type 3 tests of fixed effects, and generalized least squares means will be used along with the best fitting covariance structure. The sandwich (robust covariance matrix) estimator will also be considered. The Poisson model will be checked for overdispersion. The Poisson (or negative binomial) regression will model the logarithm of each outcome as a random variable, and the mean and variance will be estimated on the logarithmic scale. Thus, to facilitate interpretation, geometric means will be reported, which will be obtained by exponentiating the log-scaled parameter estimates. The procedures of PROC GLIMMIX in SAS software will be used to conduct the Poisson (or negative binomial) regression for Hypothesis 1c and provide a robust mechanism for handling data that are assumed missing at random (see sensitivity analysis section below for MNAR).

Secondary Aim

Data Analysis Plan for Secondary Aim 2 (Hypotheses 2a–2b)

<u>Secondary Ordinal Outcome for Aim 2: Suicidal Ideation at Baseline Period</u>

Hypothesis 2a: We expect that cortical inhibition (as assessed by pre-treatment LICI-100 collected with TMS-EMG) will have an indirect relationship with severity of suicidal ideation (as assessed by the C-SSRS severity of ideation subscale) in all subjects at baseline.

An ordinal logistic regression model will be used to estimate severity of suicidal ideation at baseline from baseline measure of LICI-100 collected with TMS-EMG. Age, sex, pubertal status, antidepressant medication status, and depression severity at baseline (CDRS-R total) will be included as covariates in the model. Maximum likelihood estimation and Type 3 tests of fixed effects will be used along with the Wald Chi-Square statistic. Robust standard errors (Huber Sandwich Estimator) will also be considered. For the ordinal logistic regression, the cumulative probabilities will be modeled over the lower-ordered suicidal ideation scale scores (less suicidal ideation). The adjusted odds ratios (i.e., odds of suicidal ideation from LICI with TMS-EMG), and 95% confidence interval, will be estimated as part of the model so as to interpret the relationship between the TMS-EMG biomarker and baseline suicidal ideation.

<u>Secondary Ordinal Outcome for Aim 2: Suicidal Ideation during the 10-day acute Trial Period</u>

Hypothesis 2b: We expect that, among the adolescents who receive active sequential bilateral aTBS treatment, there will be an indirect relationship between cortical inhibition (as assessed by LICI-100 collected with TMS-EMG) and suicidal ideation (as assessed by the C-SSRS severity of ideation subscale) over the 10 day acute treatment trial.

An ordinal logistic regression model within a GEE analysis framework similar to that described above for Hypothesis 1a will be used here to examine the relationship between cortical inhibition (LICI-100 collected with TMS-EMG as a time-varying covariate) and C-SSRS severity of ideation subscale (as the response variable) over the 10-day acute treatment trial. The parameter estimates (regression coefficients) will be interpreted from the solution for fixed effects in the GEE analysis. The adjusted odds ratio (i.e., odds of suicidal ideation from cortical

inhibition), and 95% confidence interval, will be estimated as part of the GEE model. The same covariates specified above in Hypothesis 1a will be included in the model for Hypothesis 2b.

Exploratory Aim

Data Analysis Plan for Exploratory Aim 3 (Hypotheses 3a–3b)

Exploratory Ordinal Outcome for Aim 3: Suicidal Ideation at Baseline Period

Hypothesis 3a: We expect that cortical inhibition (as assessed by pre-treatment LICI-100 and N100 collected with TMS-EEG) will have an indirect relationship with severity of suicidal ideation (as assessed by the C-SSRS severity of ideation subscale) in all participants at baseline.

An ordinal logistic regression model similar to that described above for Hypothesis 2a will be used to estimate severity of suicidal ideation at baseline from baseline measures of LICI-100 and N100. A separate model will be implemented for each measure of TMS-EEG cortical inhibition. The same covariates specified in Hypothesis 2a will be included in the model for Hypothesis 3a. The adjusted odds ratios (i.e., odds of suicidal ideation from LICI-100 and N100), and 95% confidence interval, will be estimated as part of the model so as to interpret the relationship between each TMS-EEG biomarker and baseline suicidal ideation.

Exploratory Ordinal Outcome for Aim 3: Suicidal Ideation during the 10-day acute Trial Period

Hypothesis 3b: We expect that, among the adolescents who receive active sequential bilateral aTBS treatment, there will be an indirect relationship between cortical inhibition (as assessed by TMS-EEG N45, N100) and suicidal ideation (as assessed by the C-SSRS severity of ideation subscale) over the 10 day acute treatment trial.

An ordinal logistic regression model within a GEE analysis framework similar to that described above for Hypothesis 2b will be used here to examine the relationship between cortical inhibition (LICI-100, N100 as a time-varying covariate) and C-SSRS severity of ideation subscale (as the response variable) over the 10-day acute treatment trial. A separate model will be implemented for each measure of TMS-EEG cortical inhibition. The same covariates specified in Hypothesis 2b will be included in the model for Hypothesis 3b. The parameter estimates (regression coefficients) will be interpreted from the solution for fixed effects in the GEE analysis. The adjusted odds ratio (i.e., odds of suicidal ideation from TMS-EEG cortical inhibition), and 95% confidence interval, will be estimated as part of the GEE model.

Sensitivity Analysis

In this section we present a brief sensitivity analysis plan to address incomplete response patterns according to a missing not at random mechanism. First, we will follow the outcome-based model framework of Diggle and Kenward (1994)[134] for longitudinal data with non-random missingness along with the model-based approach proposed in Carpenter et al. (2002)[135] for modeling both the treatment response and the missing data process. That is, if deemed necessary, we will build longitudinal models for the data which includes a model for the dropout (or missing data) process so as to assess the sensitivity of the results about the dependence between dropout (or missingness) and treatment response. Additionally, if deemed necessary, we will consider implementing the semi-parametric shared parameter model approach as described in Tsonaka et al. (2009)[136] to handle nonmonotone nonignorable missingness for mixed-effects models. A Pattern-Mixture Model will also be considered (if

necessary). The results from such (non-random dropout/missingness) models will then be compared with the results of the proposed standard methods (dropout or missing at random models) so as to assess the sensitivity of the results to the effect of dropouts (or incomplete response patterns according to a missing not at random mechanism).

7.3 Subject Population(s) for Analysis

- All-treated population: Any subject randomized into the study that received at least one exposure (treatment) from the study device.

8 Safety and Adverse Events

Table 5. Reporting Time Frames

Reportable Events	DSMB (Time Frame)	FDA (Time Frame)	IRB (Time Frame)	OHRP (Time Frame)
IRB/DSMB/OHRP/FDA Suspensions or Termination	Within 3 business days of receipt	Within 3 business days of receipt	Within 3 business days of receipt	Within 3 business days of receipt
Deaths related to study participation	Within 5 business days)	Within 5 business days)	Within 5 business days)	Not applicable
Unexpected Serious Adverse Events Related to Study Participation	Within 5 business days upon notification	Within 5 business days upon notification	Within 5 business days upon notification	Not applicable
Expected Serious Adverse Events Related to Study Participation	Within 5 business days upon notification	Within 5 business days upon notification	Within 5 business days upon notification	Not applicable
Unexpected/Unrelated and Expected/Unrelated Serous Adverse Events	Filed in the triannual DSMB progress reports	Filed with annual progress reports	Filed with annual progress reports	Not applicable
Unanticipated Adverse Device Effect	Within 5 business days upon notification	Within 5 business days upon notification	Within 5 business days upon notification	Not applicable
Unanticipated Problems Involving Risks to Subjects or Others	Within 5 business days upon notification	Within 5 business days upon notification	Within 5 business days upon notification	Within 5 business days upon notification
Serious Or Continuing Noncompliance	Within 5 business days upon notification	Within 5 business days upon notification	Within 5 business days upon notification	Within 5 business days upon notification
Adverse Events	Expected and unrelated are filed in the triannual DSMB progress reports	Expected and unrelated are filed with annual progress reports	Expected and unrelated are filed with annual progress reports	Not applicable

Protocol Violations	Annual progress reports	Annual progress reports	Annual progress reports	Not applicable
Pregnancy	Within 5 business days upon notification	Within 5 business days upon notification	Within 5 business days upon notification	Not applicable

Note – Some Adverse Events represent Unanticipated Problems that are required to be reported to OHRP (see: https://www.hhs.gov/ohrp/regulations-and-policy/guidance/reviewing-unanticipated-problems/index.html)

All adverse events occurring during the study, including those not meeting the criteria of an Unanticipated Adverse Device Effect (UADE) will be recorded on the appropriate case report form. Reporting time frames for the DSMB, FDA, and IRB are summarized in table 5. Expected clinical adverse events and anticipated adverse device effects include pain or discomfort under the treatment coil, eye pain, facial pain, facial numbness, toothache pain, skin pain, facial muscle twitching, blurred vision, seizures, cardiogenic syncope, and worsening mood symptoms. The DSMB will review all adverse events (expected and unexpected) in DSMB data reports submitted tri-annually. Unanticipated Adverse Device Effects (UADEs) will be reported to the DSMB, FDA, and IRB immediately (as noted in Table 5). Records of these events will be maintained and reports submitted to the FDA and IRB according to the regulatory requirements. Expected clinical adverse events and nonsignificant (not serious) clinical adverse events will be reported annually. Expected clinical adverse events and anticipated adverse device effects are those listed in Section 1.5.2.

Any pregnancy detected during the acute 10 day treatment course or a pregnancy that may have been present during the acute 10 day treatment course (based on estimated date of conception in consultation with the subject's clinical obstetrician) will be reported within 5 business days to the IRB, DSMB, and FDA. If a pregnancy is detected during the acute 10 day treatment course no further investigational treatments will be delivered but the patient will be followed for 12 months as per the study protocol. The pregnancy outcome will be documented.

8.1 Definitions

Unanticipated Adverse Device Effect (UADE)
A UADE is any serious adverse effect on health or safety or any life-threatening problem or death caused by, or associated with, a device if that effect, problem or death was not previously identified in nature, severity, or degree of incidence in the investigational plan or IDE application (including a supplementary plan or application), or any other unanticipated serious problem associated with a device that relates to the rights, safety, or welfare of subjects.

Adverse Effect (Event)
Any untoward medical occurrence in a subject involved in clinical study of an investigational device; regardless of the causal relationship of the problem with the device or, if applicable, other study related treatment(s). Adverse events will be reported to the DSMB tri-annually.

Associated with the investigational device: There is a reasonable possibility that the adverse effect may have been caused by the investigational device. "Reasonable possibility" will be determined based on the PIs experience and relevant literature.[85,102] These determinations will be reviewed with the study medical monitor, Della J. Derscheid, APRN, CNS, PhD, MS) three times per year.

Life-threatening adverse effect: Any adverse effect that places the subject, in the view of either the investigator or the sponsor, at immediate risk of death from the effect **as it occurred**. It does not include a reaction that, had it occurred in a more severe form, might have caused death.

Serious adverse effect: An adverse effect is considered "serious" if, in the view of either the investigator or the sponsor, it results in any of the following outcomes:

- death
- a life-threatening AE
- inpatient hospitalization or prolongation of existing hospitalization
- a persistent or significant disability/incapacity
- a congenital anomaly/birth defect.

Unanticipated adverse effect: Any adverse effect, the nature, specificity, severity, or frequency of which is not consistent with the risk information in the clinical study protocol or elsewhere in the current IDE application.

General Physical Examination Findings
At screening, any clinically significant abnormality should be recorded as a preexisting condition. At the end of the study, any new clinically significant findings/abnormalities that meet the definition of an adverse event must also be recorded and documented as an adverse event.

Hospitalization, Prolonged Hospitalization or Surgery
Any adverse event that results in hospitalization or prolonged hospitalization should be documented and reported as an unanticipated adverse device effect unless specifically instructed otherwise in this protocol. Any condition responsible for surgery should be documented as an adverse event if the condition meets the criteria for an adverse event.

Neither the condition, hospitalization, prolonged hospitalization, nor surgery are reported as an adverse event in the following circumstances:

- Hospitalization or prolonged hospitalization for diagnostic or elective surgical procedures for a preexisting condition. Surgery should **not** be reported as an outcome

of an adverse event if the purpose of the surgery was elective or diagnostic and the outcome was uneventful
- Hospitalization or prolonged hospitalization required to allow efficacy measurement for the study
- Hospitalization or prolonged hospitalization for therapy of the target disease of the study, unless it is a worsening or increase in frequency of hospital admissions as judged by the clinical investigator.

Post-study Adverse Event

All unresolved adverse events should be followed by the investigator until the events are resolved, the subject is lost to follow-up, or the adverse event is otherwise explained. The study team will report unresolved adverse events to the DSMB when the subject exits the study. The study team will track adverse events through resolution and report to the DSMB as feasible (if the patient can be contacted or monitored). The study team will report unresolved adverse events that cannot be tracked through resolution to the DSMB (the subject cannot be contacted or is unwilling to have contact with the study team). At the last scheduled visit, the study team will instruct each subject to report, to the local investigator, any subsequent event(s) that the subject, or the subject's personal physician, believes might reasonably be related to participation in this study. The local investigator should notify the study regulatory sponsor of any death or adverse event occurring at any time after a subject has discontinued or terminated study participation that may reasonably be related to this study. The sponsor should also be notified if the local investigator should become aware of the development of problems, cancer or of a congenital anomaly in a subsequently conceived offspring of a subject that has participated in this study.

Preexisting Condition

A preexisting condition is one that is present at the start of the study. A preexisting condition should be recorded as an adverse event if the frequency, intensity, or the character of the condition worsens during the study period.

Unanticipated Problems Involving Risk to Subjects or Others (UPIRTSO)

Any unanticipated problem or adverse event that meets all of the following three criteria:

- Serious: Serious problems or events that results in significant harm, (which may be physical, psychological, financial, social, economic, or legal) or increased risk for the subject or others (including individuals who are not research subjects). These include: (1) death; (2) life threatening adverse experience; (3) hospitalization - inpatient, new, or prolonged; (4) disability/incapacity - persistent or significant; (5) birth defect/anomaly; (6) breach of confidentiality and (7) other problems, events, or new information (i.e. publications, DSMB reports, interim findings, product labeling change) that in the opinion of the local investigator may adversely affect the rights, safety, or welfare of the subjects or others, or substantially compromise the research data, **AND**
- Unanticipated: (i.e. unexpected) problems or events are those that are not already described as potential risks in the protocol, consent document, not listed in the Investigator's Brochure, or not part of an underlying disease. A problem or event is

"unanticipated" when it was unforeseeable at the time of its occurrence. A problem or event is "unanticipated" when it occurs at an increased frequency or at an increased severity than expected, **AND**

- Related: A problem or event is "related" if it is possibly related to the research procedures.

Adverse Event Reporting Period

For this study, the study treatment follow-up period is defined as 12 months following the last administration of study treatment.

8.2 Recording of Adverse Events

At each contact with the subject, the investigator must seek information on adverse events by specific questioning and, as appropriate, by examination. Study subjects will be routinely questioned about adverse effects at study visits. Information on all adverse events should be recorded immediately in the source document, and also in the appropriate adverse event section of the case report form (CRF) or in a separate adverse event worksheet. All clearly related signs, symptoms, and abnormal diagnostic, laboratory or procedure results should recorded in the source document.

All adverse events occurring during the study period must be recorded. All observed or volunteered adverse effects (serious or non-serious) and abnormal test findings, regardless of the treatment group if applicable or suspected causal relationship to the investigational device or if applicable other study treatment or diagnostic product(s) will be recorded in the subjects' case history. For all adverse effects sufficient information will be pursued and or obtained as to permit; an adequate determination of the outcome, an assessment of the casual relationship between the adverse effect and the investigational device or, if applicable other study treatment or diagnostic product. The clinical course of each event should be followed until resolution, stabilization, or until it has been ultimately determined that the study treatment or participation is not the probable cause. Serious adverse events that are still ongoing at the end of the study period must be followed up, to determine the final outcome. Any serious adverse event that occurs after the study period and is considered to be at least possibly related to the study treatment or study participation should be recorded and reported immediately.

A form should be created for capturing information related to adverse events and submitted with the IDE application to the FDA.
Recommended information to capture on the Adverse Event CRF:

- Subject Study Number/Identifier
- Device information (model and serial number)
- Date of event onset
- Description of the event
- Indication if study treatment was discontinued, or if investigational device was removed
- Subject current status, or if the event was resolved

- Principal Investigator assessment of if the event was serious and justification for determination
- Principal Investigator assessment of causality and relationship to study treatment.

Causality and severity assessment
The sponsor-investigator will promptly review documented adverse effects and abnormal test findings to determine 1) if the abnormal test finding should be classified as an adverse effect; 2) if there is a reasonable possibility that the adverse effect was caused by the investigational device or other study treatments; and 3) if the adverse effect meets the criteria for a serious adverse effect.

If the sponsor-investigator's final determination of causality is "unknown and of questionable relationship to the investigational device or other study treatments," the adverse effect will be classified as associated with the use of the investigational device or other study treatments for reporting purposes. If the sponsor-investigator's final determination of causality is "unknown but not related to the investigational device or other study treatments," this determination and the rationale for the determination will be documented in the respective subject's case history.

8.3 Sponsor-Investigator Reporting of Unanticipated Adverse Device Effects and Unanticipated Problems

When an adverse event has been identified, the study team will take appropriate action necessary to protect the study participant and then complete the Study Adverse Event Worksheet and log. The sponsor-investigator will evaluate the event and determine the necessary follow-up and reporting required.

The sponsor-investigator will promptly review documented Unanticipated Adverse Device Effects and as necessary shall report the results of such evaluation to the FDA and DSMB within 10 working days and to the Mayo IRB within 5 working days of initial notice of the effect. Thereafter the sponsor-investigator will submit such additional reports concerning the effect as requested.

8.3.1 Sponsor-Investigator Reporting, Notifying Mayo IRB

The sponsor-investigator will report to the Mayo IRB any UPIRTSOs and Non-UPIRTSOs according to the Mayo IRB Policy and Procedures.

8.3.2 Sponsor-Investigator Reporting: Notifying the FDA

The sponsor-investigator will report to the FDA all unanticipated adverse device effects according to the required reporting timelines, formats and regulations.

The sponsor-investigator will submit a completed FDA Form 3500A to the FDA's Center for Devices and Radiological Health for any observed or reported adverse effect that is determined to be an unanticipated adverse device effect. A copy of this completed form will be provided to the DSMB and all participating sub-investigators.

The completed FDA Form 3500A will be submitted to the FDA as soon as possible and, in no event, later than 10 working days after the sponsor-investigator first receives notice of the adverse effect.

If the results of the sponsor-investigator's follow-up evaluation shows that an adverse effect that was initially determined to not constitute an unanticipated adverse device effect does, in fact, meet the requirements for reporting; the sponsor-investigator will submit a completed FDA Form 3500A as soon as possible, but in no event later than 10 working days, after the determination was made.

For each submitted FDA Form 3500A, the sponsor-investigator will identify all previously submitted reports that that addressed a similar adverse effect experience and will provide an analysis of the significance of newly reported adverse effect in light of any previous, similar report(s).

Subsequent to the initial submission of a completed FDA Form 3500A, the sponsor-investigator will submit additional information concerning the reported adverse effect as requested by the FDA.

Reporting Process
Unanticipated Adverse Device Effect reports will be submitted on FDA Form 3500A.
The contact information for submitting reports is:

Food and Drug Administration
Center for Devices and Radiological Health
Document Mail Center - WO66-G609
10903 New Hampshire Avenue
Silver Spring, Maryland 20993-0002

Deviations from the investigational plan.

The sponsor-investigator shall notify Mayo IRB (see 21 CFR 56.108(a) (3) and (4)) of any deviation from the investigational plan to protect the life or physical well-being of a subject in an emergency. Such notice shall be given as soon as possible, but in no event later than 5 working days after the emergency occurred. Except in such an emergency, prior approval by the sponsor-investigator is required for changes in or deviations from a plan, and if these changes or deviations may affect the scientific soundness of the plan or the rights, safety, or

welfare of human subjects, DSMB, FDA, and IRB notification in accordance with 21 CFR 812.35(a) also is required.

8.4 Unblinding Procedures (Breaking the Blind) (as necessary if the study is blinded).

While the safety of the participant always comes first, it is still important to seriously consider if unblinding the study therapy is necessary to ensure a participant's safety. In the event of a serious adverse device effect, the Principal Investigator will carefully assess whether breaking the blind will critically affect how a participant is treated in response to the adverse effect and whether this knowledge outweighs the implications to the scientific soundness of the study. This decision will be reviewed and approved by the study medical monitor (Della J. Derscheid, APRN, CNS, PhD, MS). In the case of most serious adverse effects, treatment would be discontinued and symptoms treated symptomatically irrespective of the knowledge of whether the treatment received was active or sham in nature. In these instances, having this information would not significantly alter the treatment of the adverse effect(s).

If the decision to break the blind is made immediately upon learning of the adverse event, this information will be reported to the DSMB, FDA, and IRB at the time of initial adverse event reporting. If the unblinding occurs after the initial reporting, the DSMB, FDA, and IRB will be notified of the action within ten working days from the time of breaking the blind.

8.5 Stopping Rules

Stopping Rules

In collaboration with the FDA, the following stopping rules have been developed.

Subject Stopping Rules

Subjects may withdraw voluntarily from the study at any time. The subject stopping rules apply to the two-week period when the subjects are receiving the study intervention. (active or sham sequential bilateral aTBS). These rules do not apply to subjects in the naturalistic 1-year follow-up portion of the study. Subjects will be withdrawn (please refer to section 4.4) from the study by the PI/Sponsor if a subject:

- experiences a seizure,
- experiences a DSM-5-confirmed treatment-induced mania,
- experiences psychotic symptoms
- is non-compliant with study procedures; or
- endorsed illicit drug use or has a positive urine drug screen during the study

Study Stopping Rules

In order to ensure that subjects are not exposed to risks across the study and that risks are evaluated the follow are study stopping rules. **These stopping rules apply to the two-week period when subjects are receiving the study intervention (active or sham sequential**

bilateral aTBS). These rules do not apply to subjects in the naturalistic 1-year follow-up portion of the study. There are no pre-determined stopping rules for events that take place during the follow up-period as suicide attempts and psychiatric hospitalizations are expected to be common events. Adverse events that occur while subjects are in the follow up period will be tracked during the long term naturalistic follow up. The study team will be evaluating the expectedness and relatedness of these events; the IRB, DSMB, FDA will provide oversight of these determinations. AEs and SAEs will be reported and reviewed by the DSMB in the data reports according to the timeframes delineated in Section 8, Table 5.

i. Two subjects who experience seizure(s).
ii. One or more subject(s) who experiences status epilepticus.
iii. One or more subject(s) who attempts or dies by suicide.
iv. Two or more Serious Adverse Events--related to the study intervention and occurring within the 2-week treatment period--in one or more subjects.

In the event these study stopping rules are met, enrollment of new subjects will cease, but all study activities will continue for enrolled subjects. The PI will consult with the DSMB and the FDA within 10 busines days upon initial notice of meeting stopping rules.

8.6 Medical Monitoring

It is the responsibility of the sponsor-investigator to oversee the safety of the study. This safety monitoring will include careful assessment and appropriate reporting of adverse events as noted above, as well as the construction and implementation of a site data and safety-monitoring plan (see Section 10 Auditing, Monitoring and Inspecting). Medical monitoring will include a regular assessment of the number and type of serious adverse events. The study has an internal (Mayo Clinic) medical monitor (Della J. Derscheid, APRN, CNS, PhD, MS). The medical monitor will be blinded to study intervention. The medical monitor will be consulted if a serious adverse device effect occurs to determine if the blind should be broken for the safety of the participant. The medical monitor will assist with the evaluation of adverse effects with respect to the reasonable possibility that the adverse effect may have been caused by the investigational device. "Reasonable possibility" will be determined based on the PIs experience and relevant literature.[85,102] These determinations will be reviewed with the study medical monitor (Della J. Derscheid, APRN, CNS, PhD, MS) three times per year.

8.6.1 Independent Data and Safety Monitoring Board

An NIMH constituted DSMB will oversee the study in accordance with the NIMH DSMB Charter guidelines.

9 Data Handling and Record Keeping

9.1 Confidentiality

Information about study subjects will be kept confidential and managed according to the requirements of the Health Insurance Portability and Accountability Act of 1996 (HIPAA). Those regulations require a signed subject authorization informing the subject of the following:

- What protected health information (PHI) will be collected from subjects in this study
- Who will have access to that information and why
- Who will use or disclose that information
- The rights of a research subject to revoke their authorization for use of their PHI.

In the event that a subject revokes authorization to collect or use PHI, the investigator, by regulation, retains the ability to use all information collected prior to the revocation of subject authorization. For subjects that have revoked authorization to collect or use PHI, attempts should be made to obtain permission to collect at least vital status (long term survival status that the subject is alive) at the end of their scheduled study period.

9.2 Source Documents

Source data comprise all information, original records of clinical findings, observations, or other activities in a clinical trial necessary for the reconstruction and evaluation of the trial. Source data are contained in source documents. Examples of these original documents, and data records include: hospital records, clinical and office charts, laboratory notes, memoranda, subjects' diaries or evaluation checklists, pharmacy dispensing records, recorded data from automated instruments, copies or transcriptions certified after verification as being accurate and complete, microfiches, photographic negatives, microfilm or magnetic media, x-rays, subject files, and records kept at the pharmacy, at the laboratories, and at medico-technical departments involved in the clinical trial. When applicable, information recorded on the CRF shall match the Source Data recorded on the Source Documents.

9.3 Case Report Forms

A Case Report Form (CRF) will be completed for each subject enrolled into the clinical study. The investigator-sponsor will review, approve and sign/date each completed CRF; the investigator-sponsor's signature serving as attestation of the investigator-sponsor's responsibility for ensuring that all clinical and laboratory data entered on the CRF are complete, accurate and authentic.

The study case report form (CRF) is the primary data collection instrument for the study. All data requested on the CRF must be recorded. All missing data must be explained. If a space on the CRF is left blank because the procedure was not done or the question was not asked, write "N/D". If the item is not applicable to the individual case, write "N/A". All entries should be printed legibly in black ink. If any entry error has been made, to correct such an error, draw a single straight line through the incorrect entry and enter the correct data above

it. All such changes must be initialed and dated. Do not obliterate, erase, or use "white-out" for errors. For clarification of illegible or uncertain entries, print the clarification above the item, then initial and date it. If the reason for the correction is not clear or needs additional explanation, neatly include the details to justify the correction.

Data Management

a) Data is entered into a REDCap database.
b) Study data will be reviewed regularly by the Investigator and Study Coordinator for the following:
    a. Subject inclusion criteria has been met
    b. Transcription of data is accurate and complete
    c. Units of measure are recorded appropriately
c) Details of the study treatments including treatment parameters, such as percent of MT for each treatment will be stored in the The MagVenture Double Blinded Reasearch Study software, and a daily summary of the treatments will be created and stored in the Subject's source document.
d) Case files will be created for each Subject where completed visit CRFs will be stored. Any adverse event CRFs will also be stored in the case file.

Data Security and Confidentiality

a) Non-electronic source document data will be stored in a locked cabinet in a secure office. Only authorized study staff will have access.
b) Electronic data will be stored on a secure database. Only authorized users will have access. All users will have unique identifiers and passwords. Sharing of log-in information is not permitted.

9.4 Records Retention

The sponsor-investigator will maintain records and essential documents related to the conduct of the study. These will include subject case histories and regulatory documents.

The sponsor-investigator will retain the specified records and reports during the study and for the longer of the following;

1. As outlined in the Mayo Clinic Research Policy Manual –"Retention of and Access to Research Data Policy" http://mayocontent.mayo.edu/research-policy/MSS_669717,

    OR

2. A period of 2 years after the latter of the following two dates: The date on which the investigation is terminated or completed, or the date that the records are no longer required for purposes of supporting a premarket approval application or a notice of completion of a product development protocol.

10 Study Monitoring, Auditing, and Inspecting

10.1 Study Monitoring Plan

The investigator will allocate adequate time for such monitoring activities. The study team will refer and adhere to a clinical monitoring plan (CMP) that will be in place and followed that will include a schedule of visits. The Investigator will also ensure that the monitor or other compliance or quality assurance reviewer is given access to all the study-related documents and study related facilities (e.g. pharmacy, diagnostic laboratory, etc.), and has adequate space to conduct the monitoring visit.

10.1.1 Clinical Monitoring
Clinical Monitoring will be conducted by the NIMH Clinical Research Education, Support, and Training (CREST) program. This monitoring includes document review, database review, and review of regulatory documents. Clinical site monitoring is conducted to ensure that the rights and well-being of human subjects are protected, that the reported trial data are accurate, complete, and verifiable, and that the conduct of the trial is compliant with currently approved protocol/amendment(s), with GCP, and with applicable regulatory requirement(s). The main features are below.

- Monitoring will be conducted throughout the study, and involve targeted data verification of key data variables
- The site PI will be provided copies of monitoring reports within 10 days of each visit and will be provided to the NIMH DSMB liaison within 30 days of the visit.
- Details of clinical site monitoring are documented in the Clinical Monitoring Plan (CMP). The CMP describes who will conduct the monitoring, at what frequency monitoring will be done, at what level of detail monitoring will be performed, and the distribution of monitoring reports.
- The site will perform internal quality management of study conduct, data collection, documentation and completion. An individualized quality management plan will be developed to describe the site's quality management.

10.1.2 Quality Assurance and Quality Control
The clinical site will have quality assurance (QA) and quality control (QC) measures in place. The site will perform internal quality management of study conduct, data and biological specimen collection, documentation and completion. An individualized quality management plan will be developed to describe a site's quality management.

Quality control (QC) procedures will be implemented beginning with the data entry system and data QC checks that will be run on the database will be generated. Any missing data or data anomalies will be communicated to the site(s) for clarification/resolution.

Following written Standard Operating Procedures (SOPs), the monitors will verify that the clinical trial is conducted and data are generated and biological specimens are collected, documented (recorded), and reported in compliance with the protocol, International Conference on Harmonisation Good Clinical Practice (ICH GCP), and applicable regulatory requirements (e.g., Good Laboratory Practices (GLP), Good Manufacturing Practices (GMP)).

The investigational site will provide direct access to all trial related sites, source data/documents, and reports for the purpose of monitoring and auditing by the NIMH, and inspection by local and regulatory authorities, including the FDA.

10.2 Auditing and Inspecting

The sponsor-investigator will permit study-related monitoring, audits, and inspections by the IRB, the monitor, and government regulatory agencies, of all study related documents (e.g., source documents, regulatory documents, data collection instruments, study data etc.). The sponsor-investigator will ensure the capability for inspections of applicable study-related facilities (e.g., pharmacy, diagnostic laboratory, etc.).

Participation as a sponsor-investigator in this study implies acceptance of potential inspection by government regulatory authorities and applicable compliance offices.

11 Ethical Considerations

This study is to be conducted according to United States government regulations and Institutional research policies and procedures.

This protocol and any amendments will be submitted to a properly constituted local Institutional Review Board (IRB), in agreement with local legal prescriptions, for formal approval of the study. The decision of the IRB concerning the conduct of the study will be made in writing to the sponsor-investigator before commencement of this study.

All subjects for this study will be provided a consent form describing this study and providing sufficient information for subjects to make an informed decision about their participation in this study. This consent form will be submitted with the protocol for review and approval by the IRB for the study. The formal consent of a subject, using the Approved IRB consent form, must be obtained before that subject undergoes any study procedure. The consent form must be signed and dated by the subject or the subject's legally authorized representative, and the individual obtaining the informed consent.

Subject confidentiality and privacy is strictly held in trust by the participating investigators, their staff, and the sponsor(s) and their interventions. This confidentiality is extended to cover the clinical information relating to subjects. Therefore, the study protocol,

documentation, data, and all other information generated will be held in strict confidence. No information concerning the study or the data will be released to any unauthorized third party without prior written approval of the sponsor.

All research activities will be conducted in as private a setting as possible. Potential subjects will have the opportunity to discuss study participation without their parent or guardian present.

The study monitor, other authorized representatives of the sponsor, representatives of theNIH, FDA, and IRB may inspect all documents and records required to be maintained by the investigator, including but not limited to, medical records.

The study subject's contact information will be securely stored at each clinical site for internal use during the study. At the end of the study, all records will continue to be kept in a secure location for as long a period as dictated by the reviewing IRB, Institutional policies, FDA (IDE) record requirements, or sponsor requirements.

As a NIMH-funded study, data sharing through the NIMH Data Archive (NDA) is required. NDA is a large database where deidentified study data from many NIMH studies are stored and managed. Any researcher who requests access to the deidentified data of the NDA is bound to adhere to strict data safety practices and to avoid any attempts at deidentification. The intention to submit deidentified subject data to the NDA is included in consent and assent documents, and participants or guardians are able to opt out of this data sharing at any time. Opting out of NDA data sharing does not in any way impact trial eligibility.

To further protect the privacy of study subjects, a Certificate of Confidentiality will be issued by the National Institutes of Health (NIH). This certificate protects identifiable research information from forced disclosure. It allows the investigator and others who have access to research records to refuse to disclose identifying information on research participation in any civil, criminal, administrative, legislative, or other proceeding, whether at the federal, state, or local level. By protecting researchers and institutions from being compelled to disclose information that would identify research participants, Certificates of Confidentiality help achieve the research objectives and promote participation in studies by helping assure confidentiality and privacy to participants.

12 Study Finances

12.1 Funding Source

National Institute of Mental Health. Grant 1R01MH124655-01

12.2 Conflict of Interest

Any study team member who has a conflict of interest with this study (patent ownership, royalties, or financial gain greater than the minimum allowable by their institution, etc.) must have the conflict reviewed by a properly constituted Conflict of Interest Committee with a

Committee-sanctioned conflict management plan that has been reviewed and approved by the study sponsor-investigator prior to participation in this study.

12.3 Subject Stipends or Payments

Subjects will also be remunerated for their time in the study. This will include $50 for the baseline assessment day, $10 for treatment days, $50 for the posttreatment day, and $10 for each monthly follow up visit for a total of $320.

13 Publication Plan

The PI/Sponsor (Dr. Croarkin) holds the primary responsibility for publications of results from the proposed study. The PI/Sponsor will register the study on ClinicalTrials.gov (https://register.clinicaltrials.gov/) prior to subject recruitment and enrollment. Results will be posted to ClinicalTrials.gov within 12 months of final data collection form the primary outcomes.

14 References

1. Fazel S, Runeson B. Suicide. *N Engl J Med.* 2020;382(3):266-274.
2. Kann L, McManus T, Harris WA, Shanklin SL, Flint KH, Queen B, Lowry R, Chyen D, Whittle L, Thornton J, Lim C, Bradford D, Yamakawa Y, Leon M, Brener N, Ethier KA. Youth Risk Behavior Surveillance - United States, 2017. *MMWR Surveill Summ.* 2018;67(8):1-114.
3. Organization WH. Preventing suicide: A global imperative. https://www.who.int/mental_health/suicide-prevention/world_report_2014/en/. Accessed 3 January 2020.
4. Miron O, Yu KH, Wilf-Miron R, Kohane IS. Suicide Rates Among Adolescents and Young Adults in the United States, 2000-2017. *JAMA.* 2019;321(23):2362-2364.
5. Olfson M, Blanco C, Wall M, Liu SM, Saha TD, Pickering RP, Grant BF. National Trends in Suicide Attempts Among Adults in the United States. *JAMA Psychiatry.* 2017;74(11):1095-1103.
6. Copeland WE, Goldston DB, Costello EJ. Adult Associations of Childhood Suicidal Thoughts and Behaviors: A Prospective, Longitudinal Analysis. *J Am Acad Child Adolesc Psychiatry.* 2017;56(11):958-965 e954.
7. Brent DA, Greenhill LL, Compton S, Emslie G, Wells K, Walkup JT, Vitiello B, Bukstein O, Stanley B, Posner K, Kennard BD, Cwik MF, Wagner A, Coffey B, March JS, Riddle M, Goldstein T, Curry J, Barnett S, Capasso L, Zelazny J, Hughes J, Shen S, Gugga SS, Turner JB. The Treatment of Adolescent Suicide Attempters study (TASA): predictors of suicidal events in an open treatment trial. *J Am Acad Child Adolesc Psychiatry.* 2009;48(10):987-996.

8. Zalsman G, Hawton K, Wasserman D, van Heeringen K, Arensman E, Sarchiapone M, Carli V, Hoschl C, Barzilay R, Balazs J, Purebl G, Kahn JP, Saiz PA, Lipsicas CB, Bobes J, Cozman D, Hegerl U, Zohar J. Suicide prevention strategies revisited: 10-year systematic review. *Lancet Psychiatry.* 2016;3(7):646-659.
9. Bennett K, Rhodes AE, Duda S, Cheung AH, Manassis K, Links P, Mushquash C, Braunberger P, Newton AS, Kutcher S, Bridge JA, Santos RG, Manion IG, McLennan JD, Bagnell A, Lipman E, Rice M, Szatmari P. A Youth Suicide Prevention Plan for Canada: A Systematic Review of Reviews. *Can J Psychiatry.* 2015;60(6):245-257.
10. Franklin JC, Ribeiro JD, Fox KR, Bentley KH, Kleiman EM, Huang X, Musacchio KM, Jaroszewski AC, Chang BP, Nock MK. Risk factors for suicidal thoughts and behaviors: A meta-analysis of 50 years of research. *Psychol Bull.* 2017;143(2):187-232.
11. Gordon JA, Frost Bellgowan JA, Lawhorn C, Scheinert RB. Challenges and Opportunities in Psychiatric Neuroscience. *Cold Spring Harb Symp Quant Biol.* 2018;83:1-8.
12. Kapur S, Phillips AG, Insel TR. Why has it taken so long for biological psychiatry to develop clinical tests and what to do about it? *Mol Psychiatry.* 2012;17(12):1174-1179.
13. Perlis RH. Translating biomarkers to clinical practice. *Mol Psychiatry.* 2011;16(11):1076-1087.
14. Croarkin PE, Nakonezny PA, Deng ZD, Romanowicz M, Voort JLV, Camsari DD, Schak KM, Port JD, Lewis CP. High-frequency repetitive TMS for suicidal ideation in adolescents with depression. *J Affect Disord.* 2018;239:282-290.
15. Bastiampillai T, Sharfstein SS, Allison S. Increasing the Use of Lithium and Clozapine in US Suicide Prevention. *JAMA Psychiatry.* 2017;74(4):423.
16. Daskalakis ZJ, Farzan F, Barr MS, Rusjan PM, Favalli G, Levinson AJ, Fitzgerald PB. Evaluating the relationship between long interval cortical inhibition, working memory and gamma band activity in the dorsolateral prefrontal cortex. *Clin EEG Neurosci.* 2008;39(3):150-155.
17. Croarkin PE, Levinson AJ, Daskalakis ZJ. Evidence for GABAergic inhibitory deficits in major depressive disorder. *Neurosci Biobehav Rev.* 2011;35(3):818-825.
18. Krnjevic K. Glutamate and gamma-aminobutyric acid in brain. *Nature.* 1970;228(5267):119-124.
19. Schmaal L, van Harmelen AL, Chatzi V, Lippard ETC, Toenders YJ, Averill LA, Mazure CM, Blumberg HP. Imaging suicidal thoughts and behaviors: a comprehensive review of 2 decades of neuroimaging studies. *Mol Psychiatry.* 2019.
20. Chang BP, Franklin JC, Ribeiro JD, Fox KR, Bentley KH, Kleiman EM, Nock MK. Biological risk factors for suicidal behaviors: a meta-analysis. *Transl Psychiatry.* 2016;6(9):e887.
21. Oquendo MA, Sullivan GM, Sudol K, Baca-Garcia E, Stanley BH, Sublette ME, Mann JJ. Toward a biosignature for suicide. *Am J Psychiatry.* 2014;171(12):1259-1277.
22. Gabbay V, Bradley KA, Mao X, Ostrover R, Kang G, Shungu DC. Anterior cingulate cortex gamma-aminobutyric acid deficits in youth with depression. *Transl Psychiatry.* 2017;7(8):e1216.

23. Gabbay V, Mao X, Klein RG, Ely BA, Babb JS, Panzer AM, Alonso CM, Shungu DC. Anterior cingulate cortex gamma-aminobutyric acid in depressed adolescents: relationship to anhedonia. *Arch Gen Psychiatry.* 2012;69(2):139-149.

24. Lewis CP, Nakonezny PA, Ameis SH, Vande Voort JL, Husain MM, Emslie GJ, Daskalakis ZJ, Croarkin PE. Cortical inhibitory and excitatory correlates of depression severity in children and adolescents. *J Affect Disord.* 2016;190:566-575.

25. Lewis CP, Nakonezny PA, Blacker CJ, Vande Voort JL, Port JD, Worrell GA, Jo HJ, Daskalakis ZJ, Croarkin PE. Cortical inhibitory markers of lifetime suicidal behavior in depressed adolescents. *Neuropsychopharmacology.* 2018;43(9):1822-1831.

26. King JD, Horton SE, Hughes JL, Eaddy M, Kennard BD, Emslie GJ, Stewart SM. The Interpersonal-Psychological Theory of Suicide in Adolescents: A Preliminary Report of Changes Following Treatment. *Suicide Life Threat Behav.* 2018;48(3):294-304.

27. Kotak VC, Mirallave A, Mowery TM, Sanes DH. GABAergic inhibition gates excitatory LTP in perirhinal cortex. *Hippocampus.* 2017;27(12):1217-1223.

28. Levitt JG, Kalender G, O'Neill J, Diaz JP, Cook IA, Ginder N, Krantz D, Minzenberg MJ, Vince-Cruz N, Nguyen LD, Alger JR, Leuchter AF. Dorsolateral prefrontal gamma-aminobutyric acid in patients with treatment-resistant depression after transcranial magnetic stimulation measured with magnetic resonance spectroscopy. *J Psychiatry Neurosci.* 2019;44(6):386-394.

29. Li CT, Huang YZ, Bai YM, Tsai SJ, Su TP, Cheng CM. Critical role of glutamatergic and GABAergic neurotransmission in the central mechanisms of theta-burst stimulation. *Hum Brain Mapp.* 2019;40(6):2001-2009.

30. George MS, Raman R, Benedek DM, Pelic CG, Grammer GG, Stokes KT, Schmidt M, Spiegel C, Dealmeida N, Beaver KL, Borckardt JJ, Sun X, Jain S, Stein MB. A two-site pilot randomized 3 day trial of high dose left prefrontal repetitive transcranial magnetic stimulation (rTMS) for suicidal inpatients. *Brain Stimul.* 2014;7(3):421-431.

31. Weissman CR, Blumberger DM, Brown PE, Isserles M, Rajji TK, Downar J, Mulsant BH, Fitzgerald PB, Daskalakis ZJ. Bilateral Repetitive Transcranial Magnetic Stimulation Decreases Suicidal Ideation in Depression. *J Clin Psychiatry.* 2018;79(3).

32. Stagg CJ, Wylezinska M, Matthews PM, Johansen-Berg H, Jezzard P, Rothwell JC, Bestmann S. Neurochemical effects of theta burst stimulation as assessed by magnetic resonance spectroscopy. *J Neurophysiol.* 2009;101(6):2872-2877.

33. McClintock SM, Reti IM, Carpenter LL, McDonald WM, Dubin M, Taylor SF, Cook IA, O'Reardon J, Husain MM, Wall C, Krystal AD, Sampson SM, Morales O, Nelson BG, Latoussakis V, George MS, Lisanby SH, National Network of Depression Centers r TMSTG, American Psychiatric Association Council on Research Task Force on Novel B, Treatments. Consensus Recommendations for the Clinical Application of Repetitive Transcranial Magnetic Stimulation (rTMS) in the Treatment of Depression. *J Clin Psychiatry.* 2018;79(1).

34. Perera T, George MS, Grammer G, Janicak PG, Pascual-Leone A, Wirecki TS. The Clinical TMS Society Consensus Review and Treatment Recommendations for TMS Therapy for Major Depressive Disorder. *Brain Stimul.* 2016;9(3):336-346.

35. Blumberger DM, Vila-Rodriguez F, Thorpe KE, Feffer K, Noda Y, Giacobbe P, Knyahnytska Y, Kennedy SH, Lam RW, Daskalakis ZJ, Downar J. Effectiveness of

theta burst versus high-frequency repetitive transcranial magnetic stimulation in patients with depression (THREE-D): a randomised non-inferiority trial. *Lancet.* 2018;391(10131):1683-1692.

36. Fitzgerald PB, Chen L, Richardson K, Daskalakis ZJ, Hoy KE. A pilot investigation of an intensive theta burst stimulation protocol for patients with treatment resistant depression. *Brain Stimul.* 2020;13(1):137-144.

37. Huang YZ, Edwards MJ, Rounis E, Bhatia KP, Rothwell JC. Theta burst stimulation of the human motor cortex. *Neuron.* 2005;45(2):201-206.

38. Mott MC, Gordon JA, Koroshetz WJ. The NIH BRAIN Initiative: Advancing neurotechnologies, integrating disciplines. *PLoS Biol.* 2018;16(11):e3000066.

39. Dhami P, Knyahnytska Y, Atluri S, Lee J, Courtney DB, Croarkin PE, Blumberger DM, Daskalakis ZJ, Farzan F. Feasibility and clinical effects of theta burst stimulation in youth with major depressive disorders: An open-label trial. *J Affect Disord.* 2019;258:66-73.

40. Hong YH, Wu SW, Pedapati EV, Horn PS, Huddleston DA, Laue CS, Gilbert DL. Safety and tolerability of theta burst stimulation vs. single and paired pulse transcranial magnetic stimulation: a comparative study of 165 pediatric subjects. *Front Hum Neurosci.* 2015;9:29.

41. Chung SW, Lewis BP, Rogasch NC, Saeki T, Thomson RH, Hoy KE, Bailey NW, Fitzgerald PB. Demonstration of short-term plasticity in the dorsolateral prefrontal cortex with theta burst stimulation: A TMS-EEG study. *Clin Neurophysiol.* 2017;128(7):1117-1126.

42. Gong N, Li Y, Cai GQ, Niu RF, Fang Q, Wu K, Chen Z, Lin LN, Xu L, Fei J, Xu TL. GABA transporter-1 activity modulates hippocampal theta oscillation and theta burst stimulation-induced long-term potentiation. *J Neurosci.* 2009;29(50):15836-15845.

43. Feng YW, Huang YQ, Yan Y, Li G, He XF, Liang FY, Pei Z, Lan Y, Xu GQ. Phasic GABA signaling mediates the protective effects of cTBS against cerebral ischemia in mice. *Neurosci Lett.* 2020;715:134611.

44. Li CT, Chen MH, Juan CH, Huang HH, Chen LF, Hsieh JC, Tu PC, Bai YM, Tsai SJ, Lee YC, Su TP. Efficacy of prefrontal theta-burst stimulation in refractory depression: a randomized sham-controlled study. *Brain.* 2014;137(Pt 7):2088-2098.

45. Kimbrell TA, Little JT, Dunn RT, Frye MA, Greenberg BD, Wassermann EM, Repella JD, Danielson AL, Willis MW, Benson BE, Speer AM, Osuch E, George MS, Post RM. Frequency dependence of antidepressant response to left prefrontal repetitive transcranial magnetic stimulation (rTMS) as a function of baseline cerebral glucose metabolism. *Biol Psychiatry.* 1999;46(12):1603-1613.

46. Teneback CC, Nahas Z, Speer AM, Molloy M, Stallings LE, Spicer KM, Risch SC, George MS. Changes in prefrontal cortex and paralimbic activity in depression following two weeks of daily left prefrontal TMS. *J Neuropsychiatry Clin Neurosci.* 1999;11(4):426-435.

47. Sonmez AI, Camsari DD, Nandakumar AL, Voort JLV, Kung S, Lewis CP, Croarkin PE. Accelerated TMS for Depression: A systematic review and meta-analysis. *Psychiatry Res.* 2019;273:770-781.

48. Seewoo B, Feindel K, Etherington S, Hennessy L, Croarkin PE, Rodger J. Validation of the Chronic Restraint Stress Model of Depression in Rats and Investigation of

Standard vs. Accelerated rTMS Treatment. *The 58th Annual Meeting of the American College of Neuropsychopharmacology.* Vol 44. Orlando, FL; 2019:122-123.

49. Williams NR, Sudheimer KD, Bentzley BS, Pannu J, Stimpson KH, Duvio D, Cherian K, Hawkins J, Scherrer KH, Vyssoki B, DeSouza D, Raj KS, Keller J, Schatzberg AF. High-dose spaced theta-burst TMS as a rapid-acting antidepressant in highly refractory depression. *Brain.* 2018;141(3):e18.

50. Lewis CP, Camsari DD, Sonmez AI, Nandakumar AL, Gresbrink MA, Daskalakis ZJ, Croarkin PE. Preliminary evidence of an association between increased cortical inhibition and reduced suicidal ideation in adolescents treated for major depression. *J Affect Disord.* 2019;244:21-24.

51. Glenn CR, Kleiman EM, Cha CB, Deming CA, Franklin JC, Nock MK. Understanding suicide risk within the Research Domain Criteria (RDoC) framework: A meta-analytic review. *Depress Anxiety.* 2018;35(1):65-88.

52. Korpi ER, Kleinman JE, Wyatt RJ. GABA concentrations in forebrain areas of suicide victims. *Biol Psychiatry.* 1988;23(2):109-114.

53. Manchon M, Kopp N, Rouzioux JJ, Lecestre D, Deluermoz S, Miachon S. Benzodiazepine receptor and neurotransmitter studies in the brain of suicides. *Life Sci.* 1987;41(24):2623-2630.

54. Smiley JF, Hackett TA, Bleiwas C, Petkova E, Stankov A, Mann JJ, Rosoklija G, Dwork AJ. Reduced GABA neuron density in auditory cerebral cortex of subjects with major depressive disorder. *J Chem Neuroanat.* 2016;76(Pt B):108-121.

55. Tao R, Davis KN, Li C, Shin JH, Gao Y, Jaffe AE, Gondre-Lewis MC, Weinberger DR, Kleinman JE, Hyde TM. GAD1 alternative transcripts and DNA methylation in human prefrontal cortex and hippocampus in brain development, schizophrenia. *Mol Psychiatry.* 2018;23(6):1496-1505.

56. Croarkin PE, Nakonezny PA, Lewis CP, Zaccariello MJ, Huxsahl JE, Husain MM, Kennard BD, Emslie GJ, Daskalakis ZJ. Developmental aspects of cortical excitability and inhibition in depressed and healthy youth: an exploratory study. *Front Hum Neurosci.* 2014;8:669.

57. Ehrlich DE, Ryan SJ, Hazra R, Guo JD, Rainnie DG. Postnatal maturation of GABAergic transmission in the rat basolateral amygdala. *J Neurophysiol.* 2013;110(4):926-941.

58. Shen H, Sabaliauskas N, Yang L, Aoki C, Smith SS. Role of alpha4-containing GABAA receptors in limiting synaptic plasticity and spatial learning of female mice during the pubertal period. *Brain Res.* 2017;1654(Pt B):116-122.

59. Croarkin PE, Nakonezny PA, Husain MM, Melton T, Buyukdura JS, Kennard BD, Emslie GJ, Kozel FA, Daskalakis ZJ. Evidence for increased glutamatergic cortical facilitation in children and adolescents with major depressive disorder. *JAMA Psychiatry.* 2013;70(3):291-299.

60. Levinson AJ, Fitzgerald PB, Favalli G, Blumberger DM, Daigle M, Daskalakis ZJ. Evidence of cortical inhibitory deficits in major depressive disorder. *Biol Psychiatry.* 2010;67(5):458-464.

61. Ziemann U, Reis J, Schwenkreis P, Rosanova M, Strafella A, Badawy R, Muller-Dahlhaus F. TMS and drugs revisited 2014. *Clin Neurophysiol.* 2015;126(10):1847-1868.

62. Ziemann U. Pharmacology of TMS. *Suppl Clin Neurophysiol.* 2003;56:226-231.

63. Ziemann U. TMS and drugs. *Clin Neurophysiol.* 2004;115(8):1717-1729.
64. Belardinelli P, Biabani M, Blumberger DM, Bortoletto M, Casarotto S, David O, Desideri D, Etkin A, Ferrarelli F, Fitzgerald PB, Fornito A, Gordon PC, Gosseries O, Harquel S, Julkunen P, Keller CJ, Kimiskidis VK, Lioumis P, Miniussi C, Rosanova M, Rossi S, Sarasso S, Wu W, Zrenner C, Daskalakis ZJ, Rogasch NC, Massimini M, Ziemann U, Ilmoniemi RJ. Reproducibility in TMS-EEG studies: A call for data sharing, standard procedures and effective experimental control. *Brain Stimul.* 2019;12(3):787-790.
65. Conde V, Tomasevic L, Akopian I, Stanek K, Saturnino GB, Thielscher A, Bergmann TO, Siebner HR. The non-transcranial TMS-evoked potential is an inherent source of ambiguity in TMS-EEG studies. *Neuroimage.* 2019;185:300-312.
66. Siebner HR, Conde V, Tomasevic L, Thielscher A, Bergmann TO. Distilling the essence of TMS-evoked EEG potentials (TEPs): A call for securing mechanistic specificity and experimental rigor. *Brain Stimul.* 2019;12(4):1051-1054.
67. Tremblay S, Rogasch NC, Premoli I, Blumberger DM, Casarotto S, Chen R, Di Lazzaro V, Farzan F, Ferrarelli F, Fitzgerald PB, Hui J, Ilmoniemi RJ, Kimiskidis VK, Kugiumtzis D, Lioumis P, Pascual-Leone A, Pellicciari MC, Rajji T, Thut G, Zomorrodi R, Ziemann U, Daskalakis ZJ. Clinical utility and prospective of TMS-EEG. *Clin Neurophysiol.* 2019;130(5):802-844.
68. Voineskos D, Blumberger DM, Zomorrodi R, Rogasch NC, Farzan F, Foussias G, Rajji TK, Daskalakis ZJ. Altered Transcranial Magnetic Stimulation-Electroencephalographic Markers of Inhibition and Excitation in the Dorsolateral Prefrontal Cortex in Major Depressive Disorder. *Biol Psychiatry.* 2019;85(6):477-486.
69. Croarkin PE, Rotenberg A. Pediatric Neuromodulation Comes of Age. *J Child Adolesc Psychopharmacol.* 2016;26(7):578-581.
70. Croarkin PE, Nakonezny PA, Husain MM, Port JD, Melton T, Kennard BD, Emslie GJ, Kozel FA, Daskalakis ZJ. Evidence for pretreatment LICI deficits among depressed children and adolescents with nonresponse to fluoxetine. *Brain Stimul.* 2014;7(2):243-251.
71. Doruk Camsari D, Lewis CP, Sonmez AI, Nandakumar AL, Gresbrink MA, Daskalakis ZJ, Croarkin PE. Transcranial Magnetic Stimulation Markers of Antidepressant Treatment in Adolescents With Major Depressive Disorder. *Int J Neuropsychopharmacol.* 2019;22(7):435-444.
72. Abujadi C, Croarkin PE, Bellini BB, Brentani H, Marcolin MA. Intermittent theta-burst transcranial magnetic stimulation for autism spectrum disorder: an open-label pilot study. *Braz J Psychiatry.* 2018;40(3):309-311.
73. Insel TR. The NIMH experimental medicine initiative. *World Psychiatry.* 2015;14(2):151-153.
74. National Insitute of Mental Health Strategic Priorities Overview. https://www.nimh.nih.gov/about/strategic-planning-reports/strategic-research-priorities/index.shtml. Accessed January 3, 2020, 2020.
75. Grabb MC, Gobburu JVS. Challenges in developing drugs for pediatric CNS disorders: A focus on psychopharmacology. *Prog Neurobiol.* 2017;152:38-57.

76. Heath A, Lindberg DR, Makowiecki K, Gray A, Asp AJ, Rodger J, Choi DS, Croarkin PE. Medium- and high-intensity rTMS reduces psychomotor agitation with distinct neurobiologic mechanisms. *Transl Psychiatry.* 2018;8(1):126.
77. Deng ZD, Wesley L, Haugen L, Port JD, Croarkin PE. Electric field induced by reeptitive transcranial magnetic stimulation in adolescents with major depressive disorders: Comparision of coil localization approaches Paper presented at: American College of Neuropsychopharmacology 55th Annual Meeting; 1 December 2016, 2016.
78. Wall CA, Croarkin PE, Maroney-Smith MJ, Haugen LM, Baruth JM, Frye MA, Sampson SM, Port JD. Magnetic Resonance Imaging-Guided, Open-Label, High-Frequency Repetitive Transcranial Magnetic Stimulation for Adolescents with Major Depressive Disorder. *J Child Adolesc Psychopharmacol.* 2016;26(7):582-589.
79. Chirita-Emandi A, Doros G, Simina IJ, Gafencu M, Puiu M. Head Circumference References for School Age Children in Western Romania. *Rev Med Chir Soc Med Nat Iasi.* 2015;119(4):1083-1091.
80. Danborno B, Nuhu P, Yandev K. Relationship between growth pattern and head dimensions in Nigerian children (5-15 years). *Internet Journal of Biological Anthropology.* 2008;2.
81. Sun Y, Farzan F, Mulsant BH, Rajji TK, Fitzgerald PB, Barr MS, Downar J, Wong W, Blumberger DM, Daskalakis ZJ. Indicators for Remission of Suicidal Ideation Following Magnetic Seizure Therapy in Patients With Treatment-Resistant Depression. *JAMA Psychiatry.* 2016;73(4):337-345.
82. Krnjevic K, Randic M, Straughan DW. Nature of a cortical inhibitory process. *J Physiol.* 1966;184(1):49-77.
83. Kujirai T, Caramia MD, Rothwell JC, Day BL, Thompson PD, Ferbert A, Wroe S, Asselman P, Marsden CD. Corticocortical inhibition in human motor cortex. *J Physiol.* 1993;471:501-519.
84. Thase ME. Bipolar depression: diagnostic and treatment considerations. *Dev Psychopathol.* 2006;18(4):1213-1230.
85. Allen CH, Kluger BM, Buard I. Safety of Transcranial Magnetic Stimulation in Children: A Systematic Review of the Literature. *Pediatr Neurol.* 2017;68:3-17.
86. Chiramberro M, Lindberg N, Isometsa E, Kahkonen S, Appelberg B. Repetitive transcranial magnetic stimulation induced seizures in an adolescent patient with major depression: a case report. *Brain Stimul.* 2013;6(5):830-831.
87. Wu SW, Maloney T, Gilbert DL, Dixon SG, Horn PS, Huddleston DA, Eaton K, Vannest J. Functional MRI-navigated repetitive transcranial magnetic stimulation over supplementary motor area in chronic tic disorders. *Brain Stimul.* 2014;7(2):212-218.
88. Tse NY, Goldsworthy MR, Ridding MC, Coxon JP, Fitzgerald PB, Fornito A, Rogasch NC. The effect of stimulation interval on plasticity following repeated blocks of intermittent theta burst stimulation. *Sci Rep.* 2018;8(1):8526.
89. Lerner AJ, Wassermann EM, Tamir DI. Seizures from transcranial magnetic stimulation 2012-2016: Results of a survey of active laboratories and clinics. *Clin Neurophysiol.* 2019;130(8):1409-1416.

90. Purushotham A, Sinha VK, Goyal N, Tikka SK. Intermittent theta burst stimulation induced seizure in a child with schizophrenia: A case report. *Brain Stimul.* 2018;11(6):1415-1416.

91. Wu SW, Shahana N, Huddleston DA, Lewis AN, Gilbert DL. Safety and tolerability of theta-burst transcranial magnetic stimulation in children. *Dev Med Child Neurol.* 2012;54(7):636-639.

92. Wu SW, Gilbert DL. Altered neurophysiologic response to intermittent theta burst stimulation in Tourette syndrome. *Brain Stimul.* 2012;5(3):315-319.

93. Oberman LM, Pascual-Leone A, Rotenberg A. Modulation of corticospinal excitability by transcranial magnetic stimulation in children and adolescents with autism spectrum disorder. *Front Hum Neurosci.* 2014;8:627.

94. Pedapati EV, Gilbert DL, Horn PS, Huddleston DA, Laue CS, Shahana N, Wu SW. Effect of 30 Hz theta burst transcranial magnetic stimulation on the primary motor cortex in children and adolescents. *Front Hum Neurosci.* 2015;9:91.

95. Pedapati EV, Gilbert DL, Erickson CA, Horn PS, Shaffer RC, Wink LK, Laue CS, Wu SW. Abnormal Cortical Plasticity in Youth with Autism Spectrum Disorder: A Transcranial Magnetic Stimulation Case-Control Pilot Study. *J Child Adolesc Psychopharmacol.* 2016;26(7):625-631.

96. Dileone M, Ranieri F, Florio L, Capone F, Musumeci G, Leoni C, Mordillo-Mateos L, Tartaglia M, Zampino G, Di Lazzaro V. Differential Effects of HRAS Mutation on LTP-Like Activity Induced by Different Protocols of Repetitive Transcranial Magnetic Stimulation. *Brain Stimul.* 2016;9(1):33-38.

97. Oberman LM, Ifert-Miller F, Najib U, Bashir S, Heydrich JG, Picker J, Rotenberg A, Pascual-Leone A. Abnormal Mechanisms of Plasticity and Metaplasticity in Autism Spectrum Disorders and Fragile X Syndrome. *J Child Adolesc Psychopharmacol.* 2016;26(7):617-624.

98. Hendriks S, Grady C, Ramos KM, Chiong W, Fins JJ, Ford P, Goering S, Greely HT, Hutchison K, Kelly ML, Kim SYH, Klein E, Lisanby SH, Mayberg H, Maslen H, Miller FG, Rommelfanger K, Sheth SA, Wexler A. Ethical Challenges of Risk, Informed Consent, and Posttrial Responsibilities in Human Research With Neural Devices: A Review. *JAMA Neurol.* 2019.

99. Hu SH, Wang SS, Zhang MM, Wang JW, Hu JB, Huang ML, Wei N, Zhou WH, Qi HL, Xu WJ, Xu Y. Repetitive transcranial magnetic stimulation-induced seizure of a patient with adolescent-onset depression: a case report and literature review. *J Int Med Res.* 2011;39(5):2039-2044.

100. George MS, Lisanby SH, Avery D, McDonald WM, Durkalski V, Pavlicova M, Anderson B, Nahas Z, Bulow P, Zarkowski P, Holtzheimer PE, 3rd, Schwartz T, Sackeim HA. Daily left prefrontal transcranial magnetic stimulation therapy for major depressive disorder: a sham-controlled randomized trial. *Arch Gen Psychiatry.* 2010;67(5):507-516.

101. O'Reardon JP, Solvason HB, Janicak PG, Sampson S, Isenberg KE, Nahas Z, McDonald WM, Avery D, Fitzgerald PB, Loo C, Demitrack MA, George MS, Sackeim HA. Efficacy and safety of transcranial magnetic stimulation in the acute treatment of major depression: a multisite randomized controlled trial. *Biol Psychiatry.* 2007;62(11):1208-1216.

102. Rossi S, Hallett M, Rossini PM, Pascual-Leone A, Safety of TMSCG. Safety, ethical considerations, and application guidelines for the use of transcranial magnetic stimulation in clinical practice and research. *Clin Neurophysiol.* 2009;120(12):2008-2039.

103. Cullen KR, Jasberg S, Nelson B, Klimes-Dougan B, Lim KO, Croarkin PE. Seizure Induced by Deep Transcranial Magnetic Stimulation in an Adolescent with Depression. *J Child Adolesc Psychopharmacol.* 2016;26(7):637-641.

104. Keel JC, Smith MJ, Wassermann EM. A safety screening questionnaire for transcranial magnetic stimulation. *Clin Neurophysiol.* 2001;112(4):720.

105. Kavanaugh BC, Aaronson ST, Clarke GN, Holtzheimer PE, Johnson CW, McDonald WM, Schneider MB, Carpenter LL. Neurocognitive Effects of Repetitive Transcranial Magnetic Stimulation With a 2-Coil Device in Treatment-Resistant Major Depressive Disorder. *J ECT.* 2018;34(4):258-265.

106. Wall CA, Croarkin PE, McClintock SM, Murphy LL, Bandel LA, Sim LA, Sampson SM. Neurocognitive effects of repetitive transcranial magnetic stimulation in adolescents with major depressive disorder. *Front Psychiatry.* 2013;4:165.

107. Young RC, Biggs JT, Ziegler VE, Meyer DA. A rating scale for mania: reliability, validity and sensitivity. *Br J Psychiatry.* 1978;133:429-435.

108. Guy W. "Clinical Global Impressions" ECDEU Assessment Manual for Psychopharmacology-Revised In: U.S. Department of Health E, and Welfare; Public Health Service, Alcohol, Drug Abuse, and Mental Health Administration; National Instiute of Mental Health ; Psychopharmacology Research Branch; Division of Extramural Research Program ed. Rockville, MD; 1976:218-222.

109. Poznanski EO, Grossman JA, Buchsbaum Y, Banegas M, Freeman L, Gibbons R. Preliminary studies of the reliability and validity of the children's depression rating scale. *J Am Acad Child Psychiatry.* 1984;23(2):191-197.

110. Posner K, Brown GK, Stanley B, Brent DA, Yershova KV, Oquendo MA, Currier GW, Melvin GA, Greenhill L, Shen S, Mann JJ. The Columbia-Suicide Severity Rating Scale: initial validity and internal consistency findings from three multisite studies with adolescents and adults. *Am J Psychiatry.* 2011;168(12):1266-1277.

111. Kennard B, Mayes T, King J, Moorehead A, Wolfe K, Hughes J, Castillo B, Smith M, Matney J, Oscarson B, Stewart S, Nakonezny P, Foxwell A, Emslie G. The Development and Feasibility Outcomes of a Youth Suicide Prevention Intensive Outpatient Program. *J Adolesc Health.* 2019;64(3):362-369.

112. Kennard BD, Silva SG, Mayes TL, Rohde P, Hughes JL, Vitiello B, Kratochvil CJ, Curry JF, Emslie GJ, Reinecke MA, March JS, Tads. Assessment of safety and long-term outcomes of initial treatment with placebo in TADS. *Am J Psychiatry.* 2009;166(3):337-344.

113. Sackeim HA. The definition and meaning of treatment-resistant depression. *J Clin Psychiatry.* 2001;62 Suppl 16:10-17.

114. Campbell G. Design Considerations for Pivotal Clinical Investigations for Medical Devices Guidance for Industry, Clinical Investigators, Institutional Review Boards and Food and Drug Administration Staff. https://www.fda.gov/media/87363/download. Accessed November 1, 2019, 2019.

115. Ignaszewski MJ, Waslick B. Update on Randomized Placebo-Controlled Trials in the Past Decade for Treatment of Major Depressive Disorder in Child and Adolescent Patients: A Systematic Review. *J Child Adolesc Psychopharmacol.* 2018.

116. Beck AT, Steer RA, Ball R, Ranieri W. Comparison of Beck Depression Inventories -IA and -II in psychiatric outpatients. *J Pers Assess.* 1996;67(3):588-597.

117. Sheehan DV, Lecrubier Y, Sheehan KH, Amorim P, Janavs J, Weiller E, Hergueta T, Baker R, Dunbar GC. The Mini-International Neuropsychiatric Interview (M.I.N.I.): the development and validation of a structured diagnostic psychiatric interview for DSM-IV and ICD-10. *J Clin Psychiatry.* 1998;59 Suppl 20:22-33;quiz 34-57.

118. Sheehan DV, Sheehan KH, Shytle RD, Janavs J, Bannon Y, Rogers JE, Milo KM, Stock SL, Wilkinson B. Reliability and validity of the Mini International Neuropsychiatric Interview for Children and Adolescents (MINI-KID). *J Clin Psychiatry.* 2010;71(3):313-326.

119. Gershon RC, Wagster MV, Hendrie HC, Fox NA, Cook KF, Nowinski CJ. NIH toolbox for assessment of neurological and behavioral function. *Neurology.* 2013;80(11 Suppl 3):S2-6.

120. Carskadon MA, Acebo C. A self-administered rating scale for pubertal development. *J Adolesc Health.* 1993;14(3):190-195.

121. Chen L, Chung SW, Hoy KE, Fitzgerald PB. Is theta burst stimulation ready as a clinical treatment for depression? *Expert Rev Neurother.* 2019;19(11):1089-1102.

122. Association AP. *Diagnostic and Statistical Manual of Mental Disorders*. Fifth ed. Arlington, VA: American Psychiatric Association 2013.

123. Slosson RL, Erford BT, Larson SL, Slosson SW. (SIT-4) Slosson Intelligence Test-4th Edition. 2017.

124. *SAS Softward Vesion 9.4* [computer program]. Version: SAS Institute, Inc, Cary, NC; 2013.

125. Beam W, Borckardt JJ, Reeves ST, George MS. An efficient and accurate new method for locating the F3 position for prefrontal TMS applications. *Brain Stimul.* 2009;2(1):50-54.

126. Mir-Moghtadaei A, Caballero R, Fried P, Fox MD, Lee K, Giacobbe P, Daskalakis ZJ, Blumberger DM, Downar J. Concordance Between BeamF3 and MRI-neuronavigated Target Sites for Repetitive Transcranial Magnetic Stimulation of the Left Dorsolateral Prefrontal Cortex. *Brain Stimul.* 2015;8(5):965-973.

127. Philip NS, Barredo J, Aiken E, Larson V, Jones RN, Shea MT, Greenberg BD, van 't Wout-Frank M. Theta-Burst Transcranial Magnetic Stimulation for Posttraumatic Stress Disorder. *Am J Psychiatry.* 2019;176(11):939-948.

128. Daskalakis ZJ, Farzan F, Barr MS, Maller JJ, Chen R, Fitzgerald PB. Long-interval cortical inhibition from the dorsolateral prefrontal cortex: a TMS-EEG study. *Neuropsychopharmacology.* 2008;33(12):2860-2869.

129. Farzan F, Barr MS, Levinson AJ, Chen R, Wong W, Fitzgerald PB, Daskalakis ZJ. Reliability of long-interval cortical inhibition in healthy human subjects: a TMS-EEG study. *J Neurophysiol.* 2010;104(3):1339-1346.

130. Lefaucheur JP, Andre-Obadia N, Antal A, Ayache SS, Baeken C, Benninger DH, Cantello RM, Cincotta M, de Carvalho M, De Ridder D, Devanne H, Di Lazzaro V, Filipovic SR, Hummel FC, Jaaskelainen SK, Kimiskidis VK, Koch G, Langguth B, Nyffeler T, Oliviero A, Padberg F, Poulet E, Rossi S, Rossini PM, Rothwell JC,

Schonfeldt-Lecuona C, Siebner HR, Slotema CW, Stagg CJ, Valls-Sole J, Ziemann U, Paulus W, Garcia-Larrea L. Evidence-based guidelines on the therapeutic use of repetitive transcranial magnetic stimulation (rTMS). *Clin Neurophysiol.* 2014;125(11):2150-2206.

131. Wall CA, Croarkin PE, Sim LA, Husain MM, Janicak PG, Kozel FA, Emslie GJ, Dowd SM, Sampson SM. Adjunctive use of repetitive transcranial magnetic stimulation in depressed adolescents: a prospective, open pilot study. *J Clin Psychiatry.* 2011;72(9):1263-1269.

132. *PASS 14 Power Analysis and Sample Size Software* [computer program]. Version. Kaysville, Utah, USA: NCSS, LLC.; 2019.

133. Benjamini Y, Hochberg Y. Controlling the False Discovery Rate - a Practical and Powerful Approach to Multiple Testing. *Journal of the Royal Statistical Society Series B-Statistical Methodology.* 1995;57(1):289-300.

134. Diggle P, Kenward M. Informative Drop-Out in Longitudinal Data Analaysis. *Journal of the Royal Statistical Society. Series C (Applied Statistics).* 1994;43(1):49-93.

135. Carpenter J, Pocock S, Lamm CJ. Coping with missing data in clinical trials: a model-based approach applied to asthma trials. *Stat Med.* 2002;21(8):1043-1066.

136. Tsonaka R, Verbeke G, Lesaffre E. A semi-parametric shared parameter model to handle nonmonotone nonignorable missingness. *Biometrics.* 2009;65(1):81-87.
